# Scoria: a Python module for manipulating 3D molecular data

**DOI:** 10.1186/s13321-017-0237-8

**Published:** 2017-09-18

**Authors:** Patrick Ropp, Aaron Friedman, Jacob D. Durrant

**Affiliations:** 1 Department of Biological Sciences, University of Pittsburgh, Pittsburgh, PA 15260 USA; 2 Biomedical Sciences Graduate Program, University of California San Diego, La Jolla, CA 92093 USA

**Keywords:** Molecular modeling, Structural biology, Computational biology, Python

## Abstract

Third-party packages have transformed the Python programming language into a powerful computational-biology tool. Package installation is easy for experienced users, but novices sometimes struggle with dependencies and compilers. This presents a barrier that can hinder the otherwise broad adoption of new tools. We present Scoria, a Python package for manipulating three-dimensional molecular data. Unlike similar packages, Scoria requires no dependencies, compilation, or system-wide installation. One can incorporate the Scoria source code directly into their own programs. But Scoria is not designed to compete with other similar packages. Rather, it complements them. Our package leverages others (e.g. NumPy, SciPy), if present, to speed and extend its own functionality. To show its utility, we use Scoria to analyze a molecular dynamics trajectory. Our FootPrint script colors the atoms of one chain by the frequency of their contacts with a second chain. We are hopeful that Scoria will be a useful tool for the computational-biology community. A copy is available for download free of charge (Apache License 2.0) at http://durrantlab.com/scoria/.

**Electronic supplementary material:**

The online version of this article (doi:10.1186/s13321-017-0237-8) contains supplementary material, which is available to authorized users.

**Graphical abstract:**
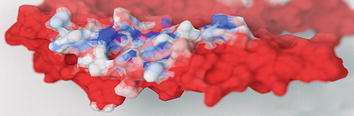
.

## Background

Even small usability barriers hinder the widespread adoption of new computational tools. When an experienced computational biologist wishes to analyze 3D molecular data, she might turn to popular Python packages such as MDAnalysis [1]. These packages have dependencies that experts can easily install from the command line. But when creating software for broader consumption (e.g., by structural biologists with less computational experience), the required dependencies sometimes complicate installation. Novice end users using Python-powered tools via graphical user interfaces may not even be familiar with the command line. To address this challenge, we present Scoria, a simpler package for manipulating 3D molecular data. Scoria has no required dependencies, so programmers can easily insert the Scoria source code directly into their existing scripts.

Python, a popular and powerful programming language for scientific computing, is an interpreted language with easy-to-read, intuitive syntax. The real power of Python comes from its extendibility. Rich Python-package ecosystems (e.g., the Python Package Index and Anaconda Cloud) extend its functionality. New Python classes and functions expose libraries written in faster languages such as C. Unfortunately, this extendibility can impact usability. Many computationalists use packages that depend on NumPy and SciPy, add-ons that speed mathematical operations. These in turn often require the installation of a C compiler. NumPy is incompatible with PyPy, a popular just-in-time Python-2.7 compiler that is faster than the standard Python executable. If a program uses a NumPy-dependent package only lightly, any speed NumPy provides may be offset by the slowness of standard Python. And third-party packages are not universally supported on all versions of Python and on all operating systems.

These challenges limit many existing molecular-modeling packages. The excellent package MDAnalysis is a good example. It depends on NumPy and so is incompatible with PyPy, it does not support the Windows operating system, and it is only partially compatible with Python 3 (“highly experimental… mostly nonfunctional and dramatically untested,” per a humorous user warning on import). Packages like MDAnalysis are also complex because of their many powerful features. While well suited for many projects, some projects need only more basic operations.

We have developed a set of functions over the last several years in service of our own published software projects [2–14]. We have integrated these functions into a single package called Scoria. Scoria has several advantages over other packages. (1) It is well suited for projects that need only basic features (e.g., loading, saving, simple structural manipulation, etc.). (2) It requires no system-wide installation, compilation, or dependencies; one need only copy the *scoria* directory into their own Python project to use it. (3) It is compatible with Python 2, Python 3, and PyPy. (4) It runs on all major operating systems (macOS, Linux, and Windows).

Though it has no *required* dependencies, Scoria does use NumPy and SciPy installations, if present, to speed and extend its own functionality. A separate, derivative version of Scoria, called Scoria MDA, can additionally leverage MDAnalysis. Scoria thus provides the best of both worlds. It is a simple, independent package for those who need basic functionality, but it provides compatibility with external libraries for those who need speed.

Scoria is useful for both analyzing molecular dynamics (MD) trajectories and molecular modeling. For example, we have used beta-version Scoria functions to create large-scale lipid-bilayer models [13], to construct small-molecules models with improved predicted binding affinities [2, 11], to measure MD-sampled binding-pocket shapes and volumes [4, 14], and to develop neural-network docking scoring functions [3, 7, 15], among other applications [5, 6, 8–10]. As an additional example, in this manuscript we describe a trajectory-analysis Scoria script that colors the atoms of one protein chain by the frequency of their contacts with a second chain.

We hope that Scoria will be a useful open-source tool for the community. Users can copy the *scoria* directory directly into their projects; alternatively, they can install Scoria via popular package managers (i.e., pip and conda). Docstrings describe the key functions, show proper usage, and present informative tables. The package itself, as well as an HTML help file generated from the docstrings, can be found online at http://durrantlab.com/scoria/.

## Implementation

### Basic features

Scoria provides functions that read and write several file types. It natively supports PDBQT as well as single and multi-frame PDB formats. Scoria also has its own single-frame molecular file format, the PYM format. PYM files have optimized read/write speeds for use in high-throughput computational projects. Finally, Scoria MDA can import other file types via MDAnalysis, both as file lists and from MDAnalysis Universe objects.

Scoria stores molecular and atomic information. It tracks individual atom names, residues, chain ids, residue sequences, occupancies, temperature factors, elements, charges, atomic coordinates, and trajectories. The user can perform calculations on the entire molecule or on subsets of atoms. Selections by atomic properties, molecular affiliations, and spatial properties define these subsets. The user can also specify custom selection criteria based on any stored atom, residue, or chain data. The user can translate and rotate molecular coordinates. Rotations about single points and lines are possible. Aside from using 3D coordinates to define these points and lines, the user can also specify atoms. In the latter case, Scoria uses the atomic coordinates. The user can also compare, contrast, align, and merge two *Molecule* objects. Scoria includes functions for calculating distances, steric clashes, and root-mean-square deviations between molecules.

For ease of use, the above functions are all accessible from the main *Molecule* class. After creating a molecule object (e.g., *mol*), the user accesses the functions via the *mol.fileio, mol.information, mol.selections, mol.manipulation*, and *mol.other_molecule* namespaces. For convenience, users can also access these functions directly from the *Molecule *object, e.g., *mol.save_pdb()* is identical to *mol.fileio.save_pdb()*.

### Rock1/shroom2 simulation: technical details

To show how Scoria can be used to analyze a molecular dynamics (MD) simulation, we ran a simulation of the rock1-dimer/shroom2 complex using Amber [16, 17] on the Frank cluster (University of Pittsburgh’s Center for Research Computing). In brief, we downloaded a model of the rock1 dimer bound to shroom2 from the Protein Data Bank (PDB ID: 5F5P) [18, 19] and retained chain A (shroom2) and chains C/D (the rock1 dimer). Visual Molecular Dynamics (VMD) [20] was next used to add sufficient Na+ cations to bring the system to electrical neutrality. We then added extra Na+ and Cl– ions to reach a concentration of 150 mmol. VMD was also used to create a water box that encompassed the entire protein complex.

The AmberTools *tleap* program [17] parameterized the system according to the *ff14SB* [21] and *ionsjc_tip3p* force fields [22]. We minimized the parameterized system in five rounds. First, only the hydrogen atoms were allowed to move. Second, most of the protein was held fixed, and the geometry of the waters was optimized. Next, most of the protein backbone was held fixed, and the side-chain geometry was optimized. Finally, all components of the system were further minimized, without any constraints.

We then restrained most of the protein backbone and heated the system. We first heated from 0 to 100 K at constant volume, and then from 100 to 300 K at constant pressure. The simulation continued at 300 K, constant pressure, during a third step of the heating phase. Following heating, the system was further equilibrated with the same backbone restraints. We then removed all restraints for a second round of equilibration. 50 ns of productive simulation followed, which were used for analysis.

## Results and discussion

Scoria is a Python package that can load, save, analyze, and manipulate 3D molecular models without requiring compilation, dependencies, or system-wide installation. Scoria’s use of NumPy and SciPy is optional; most of the basic features of the package do not require any dependencies. Extra features are available if NumPy or SciPy are present. A separate, derivative version (Scoria MDA) similarly integrates MDAnalysis support.

### Compatibility

We have tested Scoria’s basic functionality on all major operating systems using both Python and PyPy. Table 1 presents a list of all compatibility tests.

**Table 1:** Compatibility tests

OS	Python	NumPy	SciPy	MDAnalysis
macOS Sierra (10.12.3)	Python 2.7.13/Anaconda 4.2.13	1.11.2	0.18.1	0.15.0
macOS Sierra (10.12.3)	PyPy 1.8.0	N/A	N/A	N/A
Ubuntu 16.04.1 LTS	Python 2.7.13/Anaconda 4.3.14	1.11.3	0.18.1	0.15.0
Windows 10 Pro Version 1607	Python 2.7.13/Anaconda 4.3.14	1.11.3	0.18.1	N/A
Ubuntu 16.04.1 LTS	Python 3.6.0/Anaconda 4.3.14	1.11.3	0.18.1	N/A

We tested Scoria’s basic functionality on macOS 10, Ubuntu Linux, and Windows 10, using Python 2.7, Python 3.6, and PyPy 1.8. PyPy does not support NumPy, SciPy, or MDAnalysis; MDAnalysis does not support Windows; and MDAnalysis was error prone when installed under Python 3. These limitations did not prevent Scoria from passing all basic-functionality tests, though it ran slower

### Optional dependencies

Scoria’s basic features are written in pure Python. But when the user needs more intensive calculations (e.g., calculating the pairwise distances between many atoms, molecular alignments, etc.), she can install NumPy and SciPy. When she wishes to use binary formats (e.g., DCD files), she can use Scoria’s MDAnalysis-enabled version. Table 2 lists select Scoria features that are available if these other packages are installed. A more complete list is given in the Additional file 1: Table S1.

**Table 2:** Select optional Scoria functions available when third-party libraries are installed

Features			Optional dependencies		
Module	Definition	Notes	NumPy	SciPy	MDAnalysis
*fileio*	*load_pym_into*	Load PYM file	✓		
*fileio*	*load_via_MDAnalysis*	Load from file(s) via MDAnalysis	✓		✓
*fileio*	*load_MDAnalysis_into*	Load from MDAnalysis Universe object	✓		✓
*fileio*	*save_pym*	–	✓		
*selections*	*select_all_atoms_bound_to_selection*	Select atoms bound to user-specified selection	✓		
*selections*	*select_branch*	Selects an individual branch of a molecule	✓		
*selections*	*select_atoms_from_same_molecule*	Select atoms belonging to same molecule	✓		
*selections*	*selections_of_constituent_molecules*	Gets a list of all selections based on their molecule	✓		
*selections*	*select_atoms_near_other_selection*	–	✓	✓	
*selections*	*select_close_atoms_from_different_molecules*	–	✓	✓	
*manipulation*	*rotate_molecule_around_pivot_point*	–	✓		
*manipulation*	*rotate_molecule_around_pivot_atom*	–	✓		
*other_molecules*	*get_other_molecule_aligned_to_this*		✓		
*other_molecules*	*steric_clash_with_another_molecule*	–	✓	✓	
*other_molecules*	*get_distance_to_another_molecule*	–	✓	✓	
*other_molecules*	*get_rmsd_heuristic*	Calculate RMSD between two sets of atoms	✓	✓	
*atoms_and_bonds*	*create_bonds_by_distance*	Determine which atoms are bonded based on distance between them	✓	✓	

### Benchmarks

File input/output is often the greatest bottleneck when analyzing large numbers of molecular models. Scoria supports reading and writing to several formats, so we performed several benchmarks. As a test PDB file, we used 51 consecutive residues (401 atoms) taken from the 1XDN structure [23]. To calculate the average execution time, we ran each operation 100 times on a MacBook Pro (Retina, 15-inch, Mid 2015, 2.8 GHz Intel Core i7). Table 3 summarizes the results.

**Table 3:** Average Scoria execution times for various file I/O tasks, running in three different Python environments

Action	Python with NumPy/SciPy	Python without dependencies	PyPy (incompatible with dependencies)
Save PDB	0.0055 ± 0.0010	0.0085 ± 0.0016	0.0036 ± 0.0043
Load PDB (without calculating bonds by distance)	0.0104 ± 0.0010	0.0643 ± 0.0028	0.0198 ± 0.0163
Save PYM	0.0024 ± 0.0013	N/A	N/A
Load PYM	0.0008 ± 0.0004	N/A	N/A

Saving and loading PYM files requires NumPy and so could only be tested in environments with that module installed (“N/A” otherwise)

Scoria saved to a binary PYM file 2.3 times faster than to the equivalent text-formatted PDB file, and it loaded the PYM file 12.7 times faster than the PDB. PYM files are thus well suited to high-throughput projects when fast file I/O is critical.

The Python executable and installed dependencies also affected load and save times. We tested two different executables: the standard Python interpreter (sometimes called CPython) and PyPy, which uses just-in-time compilation to speed run times. We also tested CPython installations with and without dependencies such as NumPy. Loading and saving to the PYM and MDAnalysis formats requires NumPy, so only the PDB format is useful for comparing these three Python setups.

When saving the PDB file, PyPy was the fastest, despite lacking dependencies such as NumPy and SciPy. PyPy saved the test file 1.5 times faster than CPython with dependencies installed, which was in turn 1.5 times faster than CPython when those dependencies were absent. When loading the test PDB file, dependencies such as NumPy allowed CPython to load 6.2 faster than CPython without dependencies installed. PyPy support for NumPy is incomplete, but even so CPython/NumPy was only 1.9 times faster than PyPy (without NumPy).

It is easy to imagine scenarios in which PyPy would be the fastest choice. For example, we loaded the test file without calculating bonds by distance and saved 500 copies to disk (each 1 Å apart along the X axis). PyPy finished 3.1 times faster than CPython with NumPy (2.2 vs. 7.1 s) because this test script greatly favors output (saving, where PyPy excels) over input (loading).

PyPy is also faster when code includes time-consuming features that are NumPy independent. To illustrate, we wrote a script that performed 50 million variable assignments before using Scoria to load the test PDB file. CPython/NumPy loaded the molecular model faster, but PyPy accelerated variable assignment and so ran 8.3 times quicker overall (0.3 vs. 2.8 s).

### Practical demonstration

To show Scoria’s utility, we created a simple script that calculates molecular “footprints.” When simulating multi-domain systems, users may wish to identify inter-domain contacts. Surfaces with high contact residence times are likely to participate in critical inter-domain interactions. These regions may be good targets for mutagenesis studies aiming at disrupting protein–protein interfaces.

**Fig. 1:**
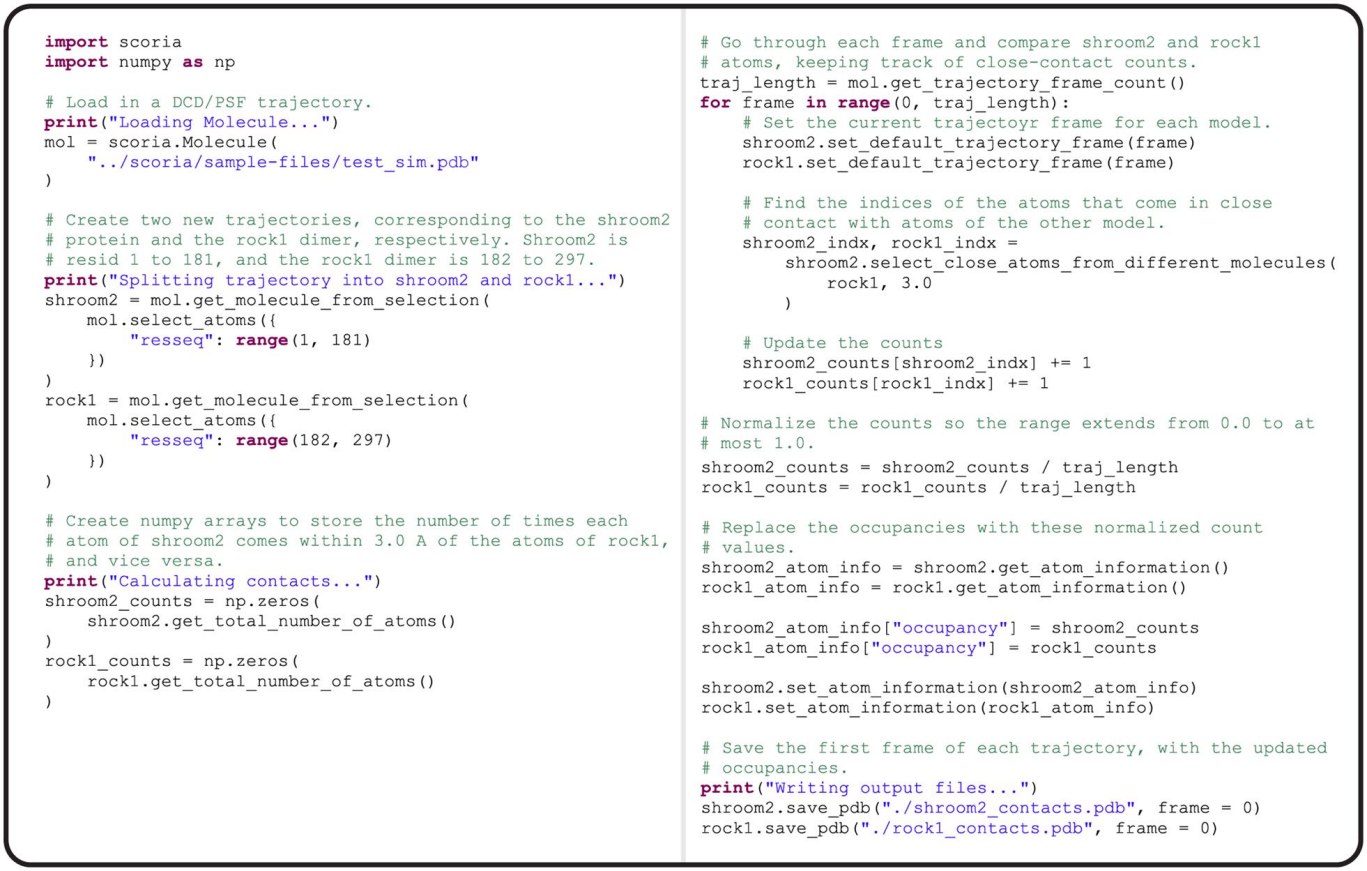
The footprint-analysis code, based on Scoria. This Python code is included with the Scoria download, in the *demo* directory

The script first loads a simulated trajectory and divides it into two parts (i.e., the two domains of interest). For each atom, it calculates a contact residence time by considering how often that atom comes within 3 Å of the other domain. Scoria stores these residence times in the occupancy fields of each atom and saves the molecular data to a PDB file. VMD [20] is then used to color the protein surface by occupancy, from red (0%) to blue (100%). The complete Scoria footprint-analysis script is shown in Fig. 1, with comments.

We applied this script to a brief MD simulation of the rock1-dimer/shroom2 complex. Previous work has shown that shroom proteins bind rho kinase (rock), regulating rock distribution and activity [19, 24, 25]. Active rock activates the actomyosin network, thereby defining cellular morphology and driving tissue morphogenesis [26]. In humans and vertebrate model systems, shroom mutations produce neural tube defects [27, 28], chronic kidney disease [29, 30], X-linked intellectual disability [31, 32], and cancer [33, 34]. In mice, a single shroom3 amino-acid change that disrupts the interaction with rock phenocopies a shroom3 null mutation [35]. These data show that the shroom-rock module is a vital, potentially druggable signaling nexus.

**Fig. 2:**
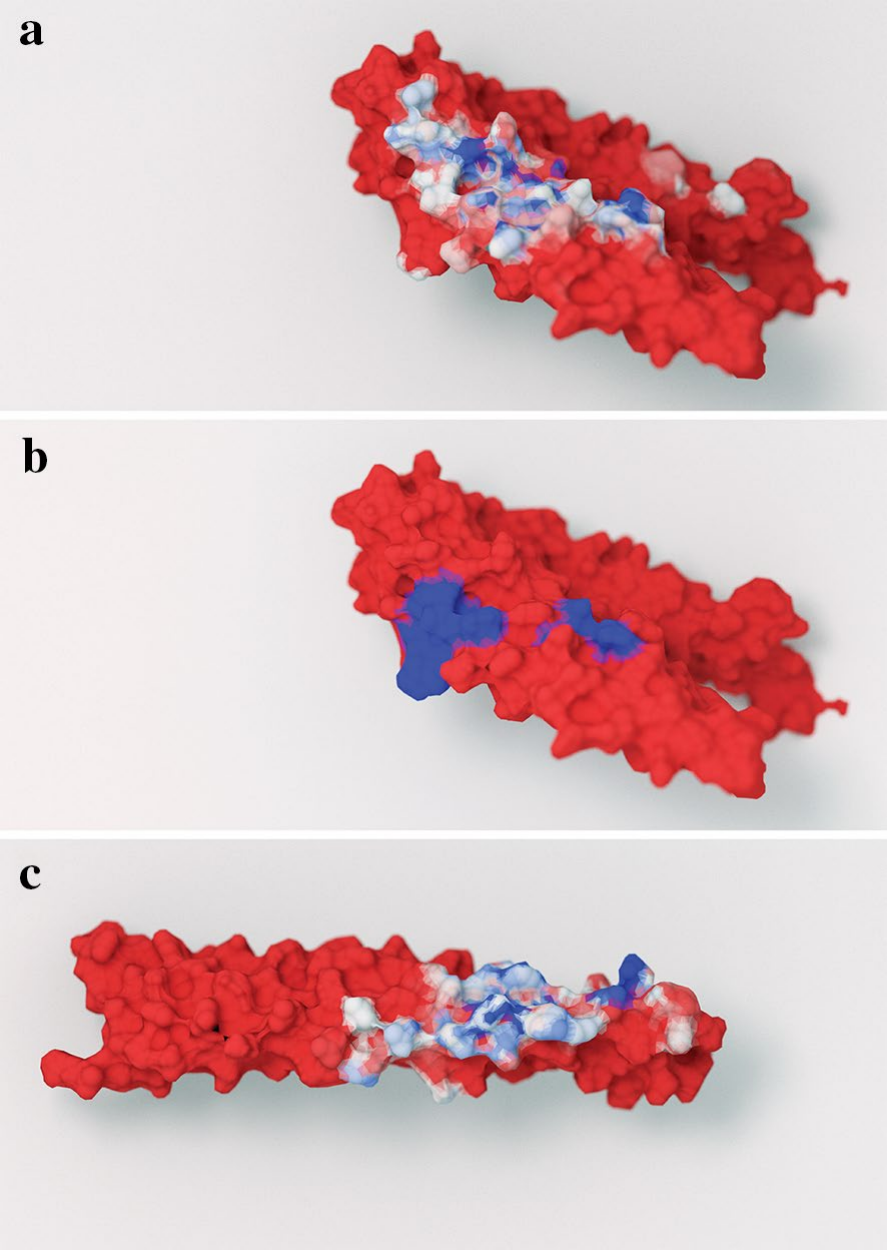
The shroom2-rock1 contact footprint. **a** The shroom2 protein. Atoms that frequently come in contact with the rock1 dimer are shown in blue. Atoms that have no contacts with rock1 are shown in red. **b** The shroom2 protein, with residues known to participate in rock1-dimer/shroom3 binding shown in blue. **c** The rho-associated protein kinase 1 (rock1) dimer. Atoms are colored by contact residence times, as in part **a**

Figure 2a, c illustrates the atomic-contact footprints of shroom2 and the rock1 dimer. For the purposes of this study, two atoms are said to be in contact if they are within 3.0 Å of each other. Colors range from red (no contact, 0%) to blue (full contact in every trajectory frame, 100%). The script considered an entire trajectory rather than a single static structure, so it is possible to distinguish between atoms that are in persistent versus transient contact with those of the neighboring chain.

Experimental evidence supports the hypothesis that the most persistent contacts are among those most critical for binding. Recent mutagenesis experiments [25] identified two regions of mouse shroom3 that are critical for rock1 binding: ^1834^SLSGRLA^1840^ and ^1878^LKENLDRR^1885^. These two regions are highly homologous to two human shroom2 regions (Fig. 2b, in blue). Both regions include residues that were persistently in contact with rock1.

A second mutagenesis study published in 2016 identified shroom2 and rock1 residues that are critical for binding [19]. Rock1 F852A and L855A mutations prevent complex formation, but Y851A, Q859A, and E862A do not. In the 5F5P structure (chain C), all these residues come within 3.0 Å of shroom2 (chain A), making it impossible to identify the most critical rock1 residues using crystallographic proximity alone.

Predicting residues critical for complex formation by considering dynamic contacts is more effective. Atoms from the critical residues F852 and L855 came within 3.0 Å of shroom2 atoms in 100 and 99% of the simulation frames. Persistent contacts show stability that promotes shroom2/rock1 binding. In contrast, Q859 and E862, which are not critical for binding, had 73 and 87% contact persistence. The analysis would have misidentified Y851 as critical (100% contact), yielding 80% accuracy overall.

The shroom2 mutations L1501A, L1548A, and K1487A are also critical [19]. Our analysis revealed 98% L1501 and 93% L1548 contact persistence. K1487, with 79% persistence, was a false negative (i.e., it is critical for binding despite having a lower contact persistence). Zalewski et al. themselves were surprised that K1487A abolished complex formation given how peripheral it is to the core binding interface [19].

While imperfect, this contact-footprint method may be useful in prospective studies aimed at prioritizing residues for mutagenesis. Regardless, that such a tool could be constructed in a few lines of code demonstrates Scoria’s utility.

## Conclusion

Scoria is a Python package for simple molecular modeling and data-collection tasks that need a light overhead. It can improve the usability of new analysis tools, encouraging broader adoption among end users who are uncomfortable installing Python dependencies.

Unlike some similar packages, Scoria is compatible with the Windows operating system as well as PyPy. If a script performs only limited molecular manipulation, the speed benefits of PyPy make Scoria an attractive option.

We will continue to add new functionality to Scoria per our ongoing projects’ needs. Additions will be broadly useful; simple (to prevent codebase bloating); and, to the extent possible, free of any required dependencies. As Scoria is an open-source project, we will make future changes publically available. Contributions from other users that meet these same inclusion criteria are welcome.

We have used Scoria components in other published software packages [2–14] under the name PyMolecule. Scoria updates these components and brings them together in a single Python package. This easy-to-use module is now available to aid other computational biologists and chemists.

## Additional files

**Additional file 1: Table S1.** Main Scoria functions, with associated dependencies (if any).

**Additional file 2.** An archived version of Scoria, without MDAnalysis support.

**Additional file 3.** An archived version of Scoria, derived from the main Scoria branch, that includes MDAnalysis support.
