# Supplementary material for: Scoria: a Python module for manipulating 3D molecular data
Source: J Cheminform. 2017 Sep 18;9:52. doi: 10.1186/s13321-017-0237-8 (PMC5603467; doi:10.1186/s13321-017-0237-8)
Supplement: Supplementary file 1 — Additional file 1: Table S1. Main Scoria functions, with associated dependencies (if any). [file 13321_2017_237_MOESM1_ESM.docx]

**Table S1.** **Main Scoria functions, with associated dependencies (if any).**

| **Features** | | | **Optional Dependencies** | | |
| --- | --- | --- | --- | --- | --- |
| **Module** | **Definition** | **Notes** | **NumPy** | **SciPy** | **MDAnalysis** |
| fileio | load_pym_into | Load PYM file | ✓ |  |  |
| fileio | load_pdbqt_trajectory_into | Load PDBQT trajectory |  |  |  |
| fileio | load_pdbqt_into | Load PDBQT file |  |  |  |
| fileio | load_pdb_trajectory_into | Load PDB trajectory |  |  |  |
| fileio | load_via_MDAnalysis | Load from file(s) via MDAnalysis | ✓ |  | ✓ |
| fileio | load_MDAnalysis_into | Load from MDAnalysis Universe object | ✓ |  | ✓ |
| fileio | get_next_frame | Divides file objects into trajectory frames |  |  |  |
| fileio | load_pdb_into | Load PDB file |  |  |  |
| fileio | save_pym | --- | ✓ |  |  |
| fileio | save_pdb | --- |  |  |  |
| information | get_filename | Get input filename |  |  |  |
| information | get_remarks | Get remarks from PDB file |  |  |  |
| information | get_atom_information | Get atom-information matrix |  |  |  |
| information | get_trajectory_coordinates | Get atomic coordinates of entire (multi-frame) trajectory |  |  |  |
| information | get_coordinates | Get atomic coordinates, single frame |  |  |  |
| information | get_coordinates_undo_point | Get previous atomic coordinates (when undo point set) |  |  |  |
| information | get_bonds | Get bond matrix |  |  |  |
| information | get_hierarchy | Returns spatial hierarchy objects |  |  |  |
| information | get_constants | Gets the constants used by the molecule |  |  |  |
| information | set_filename | Set filename |  |  |  |
| information | set_remarks | Set remarks for PDB file |  |  |  |
| information | set_atom_information | Set (updated) atom-information matrix |  |  |  |
| information | set_trajectory_coordinates | Set atomic coordinates of entire (multi-frame) trajectory |  |  |  |
| information | set_coordinates | Set atomic coordinates, single frame |  |  |  |
| information | set_coordinates_undo_point | Saves current atomic coordinates as undo point |  |  |  |
| information | set_bonds | Set bond matrix |  |  |  |
| information | set_hierarchy | Sets the spatial hierarchy objects |  |  |  |
| information | belongs_to_protein | Determines if atom belongs to protein |  |  |  |
| information | belongs_to_dna | Determines if atom belongs to DNA |  |  |  |
| information | belongs_to_rna | Determines if atom belongs to RNA |  |  |  |
| information | assign_masses | Assign atomic masses |  |  |  |
| information | assign_elements_from_atom_names | Determine atomic element from atom name |  |  |  |
| information | get_center_of_mass | --- |  |  |  |
| information | get_geometric_center | --- |  |  |  |
| information | get_total_mass | --- |  |  |  |
| information | get_total_number_of_atoms | --- |  |  |  |
| information | get_total_number_of_heavy_atoms | --- |  |  |  |
| information | get_bounding_box | Get box that surrounds system |  |  |  |
| information | get_bounding_sphere | Get sphere that surrounds system | ✓ | ✓ |  |
| information | define_molecule_chain_residue_spherical_boundaries | Finds spheres that bound the entire molecule, its chains and residues | ✓ | ✓ |  |
| information | serial_reindex | Reindex serial field |  |  |  |
| information | resseq_reindex | Reindex residue-index field |  |  |  |
| information | insert_trajectory_frame | Adds a set of coordinates as an additional timestep |  |  |  |
| information | delete_trajectory_frame | Removes a timestep from the trajectory |  |  |  |
| information | get_trajectory_frame_count | --- |  |  |  |
| information | get_default_trajectory_frame | Returns the index of the assumed frame |  |  |  |
| information | set_default_trajectory_frame | Sets the assumed time step of the trajectory |  |  |  |
| selections | select_atoms | Select atoms by properties |  |  |  |
| selections | select_atoms_in_bounding_box | Select atoms within user-specified box | ✓ |  |  |
| selections | select_all_atoms_bound_to_selection | Select atoms bound to user-specified selection | ✓ |  |  |
| selections | select_branch | Selects an individual branch of a molecule | ✓ |  |  |
| selections | select_atoms_from_same_molecule | Select atoms belonging to same molecule | ✓ |  |  |
| selections | selections_of_constituent_molecules | Gets a list of all selections based on their molecule | ✓ |  |  |
| selections | select_atoms_near_other_selection | --- | ✓ | ✓ |  |
| selections | select_atoms_in_same_residue | --- |  |  |  |
| selections | invert_selection | --- |  |  |  |
| selections | select_all | --- |  |  |  |
| selections | select_close_atoms_from_different_molecules | --- | ✓ | ✓ |  |
| selections | get_molecule_from_selection | Create new molecule from selected atoms |  |  |  |
| selections | selections_of_chains | Gets a list of all selections based on their chains | ✓ |  |  |
| selections | selections_of_residues | Gets a list of all selections based on their residues | ✓ |  |  |
| manipulation | coordinate_undo | Restores the coordinates to a previously saved state |  |  |  |
| manipulation | set_atom_location | Set location of given atom |  |  |  |
| manipulation | translate_molecule | --- |  |  |  |
| manipulation | rotate_molecule_around_a_line_between_points | --- |  |  |  |
| manipulation | rotate_molecule_around_a_line_between_atoms | --- |  |  |  |
| manipulation | rotate_molecule_around_pivot_point | --- | ✓ |  |  |
| manipulation | rotate_molecule_around_pivot_atom | --- | ✓ |  |  |
| other_molecules | get_other_molecule_aligned_to_this |  | ✓ |  |  |
| other_molecules | steric_clash_with_another_molecule | --- | ✓ | ✓ |  |
| other_molecules | merge_with_another_molecule | --- |  |  |  |
| other_molecules | get_distance_to_another_molecule | --- | ✓ | ✓ |  |
| other_molecules | get_rmsd_equivalent_atoms_specified | Calculate RMSD between two sets of atoms |  |  |  |
| other_molecules | get_rmsd_order_dependent | Calculate RMSD between two sets of atoms |  |  |  |
| other_molecules | get_rmsd_heuristic | Calculate RMSD between two sets of atoms | ✓ | ✓ |  |
| atoms_and_bonds | create_bonds_by_distance | Determine which atoms are bonded based on distance between them | ✓ | ✓ |  |
| atoms_and_bonds | get_number_of_bond_partners_of_element | For given atom, count number of atoms bonded to it that are of given element | ✓ |  |  |
| atoms_and_bonds | get_index_of_first_bond_partner_of_element | For given atom, return index of first atom bonded to it that is of given element | ✓ | ✓ |  |
| atoms_and_bonds | delete_bond | --- |  |  |  |
| atoms_and_bonds | add_bond | --- |  |  |  |
| atoms_and_bonds | delete_atom | --- |  |  |  |
| atoms_and_bonds | add_atom | --- |  |  |  |
