# Supplementary material for: Scoria: a Python module for manipulating 3D molecular data
Source: J Cheminform. 2017 Sep 18;9:52. doi: 10.1186/s13321-017-0237-8 (PMC5603467; doi:10.1186/s13321-017-0237-8)
Supplement: Supplementary file 2 — Additional file 2. An archived version of Scoria, without MDAnalysis support. [file 13321_2017_237_MOESM2_ESM.zip › scoria-1.0.0/docs/build/html/Demo.html]

2. Scoria Demo — scoria 2.0 documentation


### Navigation

- index
- modules |
- next |
- previous |
- scoria 2.0 documentation »

# 2. Scoria Demo¶

This is a quick introduction to a sample use case of the scoria library.
We’ll be looking at the points of contact between Shroom2 and Rock1.

We’ll be using scoria and numpy.

```
import scoria
import numpy as np
```

First, we’ll be importing the simulation files. (Note, that our Python 3
implementation does not handle import of DCD format files, but should in the
near future.)

```
print("Loading Molecule...")
mol = scoria.Molecule()
mol.load_via_MDAnalysis(
    "../scoria/sample-files/test_sim.psf",
    "../scoria/sample-files/test_sim.dcd"
)
```

First, we’ll split the trajectories, corresponding to the shroom2 protein
and the rock1 dimer, respectively. Shroom2 is resid 1 to 181, and the rock1
dimer is 182 to 297.

```
print("Splitting trajectory into shroom2 and",)
print("rock1...")
shroom2 = mol.get_molecule_from_selection(
    mol.select_atoms({
        "resseq": range(1, 181)
    })
)
rock1 = mol.get_molecule_from_selection(
    mol.select_atoms({
        "resseq": range(182, 297)
    })
)
```

We’ll next create numpy arrays to store the number of times each atom of
shroom2 comes within 3.0 A of the atoms of rock1, and vice versa.

```
print("Calculating contacts...")
shroom2_counts = np.zeros(
    shroom2.get_total_number_of_atoms()
)
rock1_counts = np.zeros(
    rock1.get_total_number_of_atoms()
)
```

Next, we’ll iterate through each frame and compare shroom2 and rock1 atoms,
keeping track of close-contact counts.

```
traj_length = mol.get_trajectory_frame_count()
for frame in range(0, traj_length):
    # Set the current trajectoyr frame for each model.
    shroom2.set_default_trajectory_frame(frame)
    rock1.set_default_trajectory_frame(frame)

    # Find the indices of the atoms that come in close
    # contact with atoms of the other model.
    shroom2_indx, rock1_indx =
        shroom2.select_close_atoms_from_different_molecules(
            rock1, 3.0
        )

    # Update the counts
    shroom2_counts[shroom2_indx] += 1
    rock1_counts[rock1_indx] += 1
```

Once we have these counts for the trajectory, we’ll normalize them so the range
extends from 0.0 to 1.0. Then, we’ll replace the occupancy field with the newly
normalized contact counts.

```
shroom2_counts = shroom2_counts / traj_length
rock1_counts = rock1_counts / traj_length

shroom2_atom_info = shroom2.get_atom_information()
rock1_atom_info = rock1.get_atom_information()

shroom2_atom_info["occupancy"] = shroom2_counts
rock1_atom_info["occupancy"] = rock1_counts

shroom2.set_atom_information(shroom2_atom_info)
rock1.set_atom_information(rock1_atom_info)
```

Finally, we’ll save the outputs as two PDB files for later analysis.

```
print("Writing output files...")
shroom2.save_pdb("./shroom2_contacts.pdb", frame = 0)
rock1.save_pdb("./rock1_contacts.pdb", frame = 0)
```

#### Previous topic

1. The Molecule Class

#### Next topic

3. The AtomsAndBonds Class

### This Page

- Show Source

### Quick search

### Navigation

- index
- modules |
- next |
- previous |
- scoria 2.0 documentation »

© Copyright 2016, Jacob Durrant.
Created using Sphinx 1.4.6.
