# Supplementary material for: Scoria: a Python module for manipulating 3D molecular data
Source: J Cheminform. 2017 Sep 18;9:52. doi: 10.1186/s13321-017-0237-8 (PMC5603467; doi:10.1186/s13321-017-0237-8)
Supplement: Supplementary file 2 — Additional file 2. An archived version of Scoria, without MDAnalysis support. [file 13321_2017_237_MOESM2_ESM.zip › scoria-1.0.0/docs/build/html/FileIO.html]

4. The FileIO Class — scoria 2.0 documentation


### Navigation

- index
- modules |
- next |
- previous |
- scoria 2.0 documentation »

# 4. The FileIO Class¶

## 4.1. File Types and Formats¶

Scoria can naitvely read and write PDB, PDBQT, and PYM files. It
can also read multiframe PDB and PDBQT files. It also has the ability
to import information from `MDAnalysis` objects.

## 4.2. Function Definitions¶

*class* `scoria.FileIO.``FileIO`(*parent\_molecule\_object*)¶
:   A class for saving and loading molecular data into a scoria.Molecule
    object.

    `load_MDAnalysis_into`(*\*args*)¶
    :   Allows import of molecular structure with MDAnalysis.

        Requires the `MDAnalysis` library.

        Should be called via the wrapper function
        `load_MDAnalysis_into()`

        |  |  |
        | --- | --- |
        | Params \*args: | Filename, filenames, or list of file names. Used to inizalize a MDAnalysis.Universe object. |

    `load_MDAnalysis_into_using_universe_object`(*universe*)¶
    :   Allows import of molecular structure from an MDAnalysis object.

        Requires the `MDAnalysis` library.

        Should be called via the wrapper function
        `load_MDAnalysis_into()`

        |  |  |
        | --- | --- |
        | Parameters: | **universe** (*mdanalysis.universe*) – An MDAnalysis universe object to import. |

    `load_pdb_into`(*filename*, *bonds\_by\_distance=False*, *serial\_reindex=True*, *resseq\_reindex=False*, *is\_trajectory=False*)¶
    :   Loads the molecular data contained in a pdb file into the current
        scoria.Molecule object.

        Should be called via the wrapper function `load_pdb_into()`

        |  |  |
        | --- | --- |
        | Parameters: | - **filename** (*str*) – A string, the filename of the pdb file. - **bonds\_by\_distance** (*bool*) – An optional boolean, whether or not to   determine atomic bonds based on atom proximity. True by   default. - **serial\_reindex** (*bool*) – An optional boolean, whether or not to   reindex the pdb serial field. True by default. - **resseq\_reindex** (*bool*) – An optional boolean, whether or not to   reindex the pdb resseq field. False by default. - **is\_trajectory** (*bool*) – An optional boolean, whether or not the PDB   is multi-frame. |

    `load_pdb_into_using_file_object`(*file\_obj*, *bonds\_by\_distance=False*, *serial\_reindex=True*, *resseq\_reindex=False*, *is\_trajectory=False*)¶
    :   Loads molecular data from a python file object (pdb formatted) into
        the current scoria.Molecule object. Note that most users will want
        to use the load\_pdb\_into() function instead, which is identical except
        that it accepts a filename string instead of a python file object.

        Should be called via the wrapper function
        `load_pdb_into_using_file_object()`

        |  |  |
        | --- | --- |
        | Parameters: | - **file\_obj** (*file*) – A python file object, containing pdb-formatted   data. - **bonds\_by\_distance** (*bool*) – An optional boolean, whether or not to   determine atomic bonds based on atom proximity. True by   default. - **serial\_reindex** (*bool*) – An optional boolean, whether or not to   reindex the pdb serial field. True by default. - **resseq\_reindex** (*bool*) – An optional boolean, whether or not to   reindex the pdb resseq field. False by default. - **is\_trajectory** (*bool*) – An optional boolean, whether or not the PDB   is multi-frame. |

    `load_pdb_trajectory_into`(*filename*, *bonds\_by\_distance=False*, *serial\_reindex=True*, *resseq\_reindex=False*)¶
    :   Loads the molecular data contained in a pdb trajectory file into the
        current scoria.Molecule object.

        Should be called via the wrapper function `scoria.Molecule.Molecule.load_pdb_trajectory_into()`

        |  |  |
        | --- | --- |
        | Parameters: | - **filename** (*str*) – A string, the filename of the pdb trajectory   file. - **bonds\_by\_distance** (*bool*) – An optional boolean, whether or not to   determine atomic bonds based on atom proximity. True by   default. - **serial\_reindex** (*bool*) – An optional boolean, whether or not to   reindex the pdb serial field. True by default. - **resseq\_reindex** (*bool*) – An optional boolean, whether or not to   reindex the pdb resseq field. False by default. |

    `load_pdb_trajectory_into_using_file_object`(*file\_obj*, *bonds\_by\_distance=False*, *serial\_reindex=True*, *resseq\_reindex=False*)¶
    :   Loads molecular data from a python file object (pdb trajectory
        formatted) into the current scoria.Molecule object. Note that most
        users will want to use the load\_pdb\_trajectory\_into() function
        instead, which is identical except that it accepts a filename string
        instead of a python file object.

        Should be called via the wrapper function `scoria.Molecule.Molecule.load_pdb_trajectory_into_using_file_object()`

        |  |  |
        | --- | --- |
        | Parameters: | - **file\_obj** (*file*) – A python file object, containing pdb-formatted   trajectory data. - **bonds\_by\_distance** (*bool*) – An optional boolean, whether or not to   determine atomic bonds based on atom proximity. True by   default. - **serial\_reindex** (*bool*) – An optional boolean, whether or not to   reindex the pdb serial field. True by default. - **resseq\_reindex** (*bool*) – An optional boolean, whether or not to   reindex the pdb resseq field. False by default. |

    `load_pdbqt_into`(*filename*, *bonds\_by\_distance=False*, *serial\_reindex=True*, *resseq\_reindex=False*, *is\_trajectory=False*)¶
    :   Loads the molecular data contained in a pdbqt file into the current
        scoria.Molecule object. Note that this implementation is
        incomplete. It doesn’t save atomic charges, for example. The atom
        types are stored in the “element\_padded” and “element” columns.

        Should be called via the wrapper function
        `load_pdbqt_into()`

        |  |  |
        | --- | --- |
        | Parameters: | - **filename** (*str*) – A string, the filename of the pdbqt file. - **bonds\_by\_distance** (*bool*) – An optional boolean, whether or not to   determine atomic bonds based on atom proximity. False by   default, unlike for PDB. - **serial\_reindex** (*bool*) – An optional boolean, whether or not to   reindex the pdb serial field. True by default. - **resseq\_reindex** (*bool*) – An optional boolean, whether or not to   reindex the pdbqt resseq field. False by default. - **is\_trajectory** (*bool*) – An optional boolean, whether or not the PDB   is multi-frame. Defaults of False. |

    `load_pdbqt_into_using_file_object`(*file\_obj*, *bonds\_by\_distance=False*, *serial\_reindex=True*, *resseq\_reindex=False*, *is\_trajectory=False*)¶
    :   Loads molecular data from a python file object (pdbqt formatted)
        into the current scoria.Molecule object. Note that most users will
        want to use the load\_pdb\_into() function instead, which is identical
        except that it accepts a filename string instead of a python file
        object.

        Should be called via the wrapper function
        `load_pdbqt_into_using_file_object()`

        |  |  |
        | --- | --- |
        | Parameters: | - **file\_obj** (*file*) – A python file object, containing pdb-formatted   data. - **bonds\_by\_distance** (*bool*) – An optional boolean, whether or not to   determine atomic bonds based on atom proximity. False by   default, unlike for PDB. - **serial\_reindex** (*bool*) – An optional boolean, whether or not to   reindex the pdb serial field. True by default. - **resseq\_reindex** (*bool*) – An optional boolean, whether or not to   reindex the pdb resseq field. False by default. - **is\_trajectory** (*bool*) – An optional boolean, whether or not the PDB   is multi-frame. |

    `load_pdbqt_trajectory_into`(*filename*, *bonds\_by\_distance=False*, *serial\_reindex=True*, *resseq\_reindex=False*)¶
    :   Loads the molecular data contained in a pdbqt trajectoy file (e.g., an
        AutoDock Vina output file) into the current scoria.Molecule
        object.

        Should be called via the wrapper function `scoria.Molecule.Molecule.load_pdbqt_trajectory_into()`

        |  |  |
        | --- | --- |
        | Parameters: | - **filename** (*str*) – A string, the filename of the pdbqt file. - **bonds\_by\_distance** (*bool*) – An optional boolean, whether or not to   determine atomic bonds based on atom proximity. True by   default. - **serial\_reindex** (*bool*) – An optional boolean, whether or not to   reindex the pdb serial field. True by default. - **resseq\_reindex** (*bool*) – An optional boolean, whether or not to   reindex the pdb resseq field. False by default. |

    `load_pdbqt_trajectory_into_using_file_object`(*file\_obj*, *bonds\_by\_distance=False*, *serial\_reindex=True*, *resseq\_reindex=False*)¶
    :   Loads molecular data from a python file object (pdbqt trajectory
        formatted) into the current scoria.Molecule object. Note that most
        users will want to use the load\_pdbqt\_trajectory\_into() function
        instead, which is identical except that it accepts a filename string
        instead of a python file object.

        Should be called via the wrapper function
        `scoria.Molecule.Molecule.load_pdbqt_trajectory_into_using_file_object()`

        |  |  |
        | --- | --- |
        | Parameters: | - **file\_obj** (*file*) – A python file object, containing pdbqt-formatted   trajectory data. - **bonds\_by\_distance** (*bool*) – An optional boolean, whether or not to   determine atomic bonds based on atom proximity. True by   default. - **serial\_reindex** (*bool*) – An optional boolean, whether or not to   reindex the pdb serial field. True by default. - **resseq\_reindex** (*bool*) – An optional boolean, whether or not to   reindex the pdb resseq field. False by default. |

    `load_pym_into`(*filename*)¶
    :   Loads the molecular data contained in a pym file into the current
        scoria.Molecule object.

        Requires the `numpy` library.

        Should be called via the wrapper function `load_pym_into()`

        |  |  |
        | --- | --- |
        | Parameters: | **filename** (*str*) – A string, the filename of the pym file. |

    `save_pdb`(*filename=''*, *serial\_reindex=True*, *resseq\_reindex=False*, *return\_text=False*, *frame=None*)¶
    :   Saves the molecular data contained in a scoria.Molecule object
        to a pdb file.

        Should be called via the wrapper function `save_pdb()`

        |  |  |
        | --- | --- |
        | Parameters: | - **filename** (*str*) – An string, the filename to use for saving. - **serial\_reindex** (*bool*) – An optional boolean, whether or not to   reindex the pdb serial field. True by default. - **resseq\_reindex** (*bool*) – An optional boolean, whether or not to   reindex the pdb resseq field. False by default. - **return\_text** (*bool*) – An optional boolean, whether or not to return   text instead of writing to a file. If True, the filename   variable is ignored. - **frame** (*int*) – If specified, a single-frame PDB will be generated.   If not specified, a multi-frame PDB will be generated if   the Molecule has multiple frames. Otherwise, the single   existing frame will be used. |
        | Returns: | If return\_text is True, a PDB-formatted string. Otherwise, returns nothing. |
        | Return type: | *str* or *None* |

    `save_pym`(*filename*, *save\_bonds=False*, *save\_filename=False*, *save\_remarks=False*, *save\_hierarchy=False*, *save\_coordinates\_undo\_point=False*)¶
    :   Saves the molecular data contained in a scoria.Molecule object
        to a pym file.

        Requires the `numpy` library.

        Should be called via the wrapper function
        `save_pym()`

        |  |  |
        | --- | --- |
        | Parameters: | - **filename** (*str*) – An string, the filename to use for saving. (Note   that this is actually a directory, not a file.) - **save\_bonds** (*bool*) – An optional boolean, whether or not to save   information about atomic bonds. False by default. - **save\_filename** (*bool*) – An optional boolean, whether or not to save   the original (pdb) filename. False by default. - **save\_remarks** (*bool*) – An optional boolean, whether or not to save   remarks associated with the molecule. False by default. - **save\_hierarchy** (*bool*) – An optional boolean, whether or not to save   information about spheres the bound (encompass) the whole   molecule, the chains, and the residues. False by default. - **save\_coordinates\_undo\_point** (*bool*) – An optional boolean, whether or   not to save the last coordinate undo point. False by   default. |

### Table Of Contents

- 4. The FileIO Class
  - 4.1. File Types and Formats
  - 4.2. Function Definitions

#### Previous topic

3. The AtomsAndBonds Class

#### Next topic

5. The Geometry class

### This Page

- Show Source

### Quick search

### Navigation

- index
- modules |
- next |
- previous |
- scoria 2.0 documentation »

© Copyright 2016, Jacob Durrant.
Created using Sphinx 1.4.6.
