# Supplementary material for: Scoria: a Python module for manipulating 3D molecular data
Source: J Cheminform. 2017 Sep 18;9:52. doi: 10.1186/s13321-017-0237-8 (PMC5603467; doi:10.1186/s13321-017-0237-8)
Supplement: Supplementary file 2 — Additional file 2. An archived version of Scoria, without MDAnalysis support. [file 13321_2017_237_MOESM2_ESM.zip › scoria-1.0.0/docs/build/html/index.html]

Welcome to Scoria’s documentation! — scoria 2.0 documentation


### Navigation

- index
- modules |
- next |
- scoria 2.0 documentation »

# Welcome to Scoria’s documentation!¶

Scoria is a lightweight molecular dynamics library

- 1. The Molecule Class
  - 1.1. Initiating and using the object
  - 1.2. Function Definitions
- 2. Scoria Demo
- 3. The AtomsAndBonds Class
  - 3.1. Rationale of the AtomsAndBonds functions
  - 3.2. Function Definitions
- 4. The FileIO Class
  - 4.1. File Types and Formats
  - 4.2. Function Definitions
- 5. The Geometry class
  - 5.1. Rationale of the Geometry functions
  - 5.2. Function Definitions
- 6. The Information class
  - 6.1. Rationale of the Information functions
  - 6.2. Function Definitions
- 7. The Manipulation class
  - 7.1. Rationale of the Manipulation functions
  - 7.2. Function Definitions
- 8. The OtherMolecules class
  - 8.1. Using the OtherMolecules functions
  - 8.2. Function Definitions
- 9. The Quaternion object
  - 9.1. Using Quternion object
  - 9.2. Function Definitions
- 10. The Selections class
  - 10.1. Using the Selection functions
  - 10.2. Function Definitions

# Indices and tables¶

- Index
- Module Index
- Search Page

### Table Of Contents

- Welcome to Scoria’s documentation!
- Indices and tables

#### Next topic

1. The Molecule Class

### This Page

- Show Source

### Quick search

### Navigation

- index
- modules |
- next |
- scoria 2.0 documentation »

© Copyright 2016, Jacob Durrant.
Created using Sphinx 1.4.6.
