# Supplementary material for: Scoria: a Python module for manipulating 3D molecular data
Source: J Cheminform. 2017 Sep 18;9:52. doi: 10.1186/s13321-017-0237-8 (PMC5603467; doi:10.1186/s13321-017-0237-8)
Supplement: Supplementary file 2 — Additional file 2. An archived version of Scoria, without MDAnalysis support. [file 13321_2017_237_MOESM2_ESM.zip › scoria-1.0.0/docs/build/html/Molecule.html]

1. The Molecule Class — scoria 2.0 documentation


### Navigation

- index
- modules |
- next |
- previous |
- scoria 2.0 documentation »

# 1. The Molecule Class¶

## 1.1. Initiating and using the object¶

The Molecule object is the main object class within the scoria
module. All other classes (with the exception of the
`Quarterion`) are initiated with, and hold the
relevant functions and variables for, the Molecule object. This class
contains wrapper functions to *nearly* every function belonging to
it’s children classes.

## 1.2. Function Definitions¶

*class* `scoria.Molecule.``Molecule`(*\*args*)¶
:   Loads, saves, and manupulates molecuar models. The main scoria
    class. Contains Wrapper functions for subclasses.

    Examples assume:

    ```
    >>> import scoria
    >>> PSF = "./test_file.psf"
    >>> DCD = "./test_file.dcd"
    >>> mol = scoria.Molecule()
    >>> mol.load_MDAnalysis_into(PSF, DCD)
    ```

    `add_atom`(*record\_name='ATOM'*, *serial=1*, *name='X'*, *resname='XXX'*, *chainid='X'*, *resseq=1*, *occupancy=0.0*, *tempfactor=0.0*, *charge=''*, *element='X'*, *coordinates=array([ 0.*, *0.*, *0.])*, *autoindex=True*)¶
    :   Adds an atom.

        Wrapper function for `add_atom()`

        |  |  |
        | --- | --- |
        | Parameters: | - **record\_name** (*str*) – An optional string, the record name of the atom.   “ATOM” is the default. - **serial** (*int*) – An optional int, the serial field of the atom. 1 is   the default. - **name** (*str*) – An optional string, the name of the atom. ‘X’ is the   default. - **resname** (*str*) – An optional string, the resname of the atom. ‘XXX’   is the default. - **chainid** (*str*) – An optional string, chainid of the atom. ‘X’ is the   default. - **resseq** (*int*) – An optional int, the resseq field of the atom. 1 is   the default. - **occupancy** (*float*) – An optional float, the occupancy of the atom. 0.0   is the default. - **tempfactor** (*float*) – An optional float, the tempfactor of the atom.   0.0 is the default. - **charge** (*str*) – An optional string, the charge of the atom. ‘’ is the   default. - **element** (*str*) – An optional string, the element of the atom. ‘X’ is   the default. - **coordinates** (*numpy.array*) – An optional numpy.array, the (x, y, z)   coordinates of the atom. numpy.array([0.0, 0.0, 0.0]) is   the default. |

    `add_bond`(*index1*, *index2*, *order=1*)¶
    :   Adds a bond.

        Wrapper function for `add_bond()`

        |  |  |
        | --- | --- |
        | Parameters: | - **index1** (*int*) – An int, the index of the first atom of the bonded   pair. - **index2** (*int*) – An int, the index of the second atom of the bonded   pair. - **order** (*int*) – An optional int, the order of the bond. 1 by default. |

    `assign_elements_from_atom_names`(*selection=None*)¶
    :   Determines the elements of all atoms from the atom names. Note that
        this will overwrite any existing element assignments, including those
        explicitly specified in loaded files. Note that this doesn’t populate
        elements\_stripped.

        Wrapper function for `assign_elements_from_atom_names()`

        |  |  |
        | --- | --- |
        | Parameters: | **selection** (*numpy.array*) – An optional numpy.array containing the indices of the atoms to consider when calculating the center of mass. If ommitted, all atoms of the scoria.Molecule object will be considered. |

    `assign_masses`()¶
    :   Assigns masses to the atoms of the scoria.Molecule object.

        Wrapper function for `assign_masses()`

        `Note`:
        This will autopopulate the masses according to their element
        identification and takes no input.

    `belongs_to_dna`(*atom\_index*)¶
    :   Checks if the atom is part of DNA.

        Wrapper function for `belongs_to_dna()`

        |  |  |
        | --- | --- |
        | Parameters: | **atom\_index** (*int*) – An int, the index of the atom to consider. |
        | Returns: | A boolean. True if part of dna, False if not. |

    `belongs_to_protein`(*atom\_index*)¶
    :   Checks if the atom is part of a protein. Taken primarily from Amber
        residue names.

        Wrapper function for `belongs_to_protein()`

        |  |  |
        | --- | --- |
        | Parameters: | **atom\_index** (*int*) – An int, the index of the atom to consider. |
        | Returns: | A boolean. True if part of protein, False if not. |

    `belongs_to_rna`(*atom\_index*)¶
    :   Checks if the atom is part of RNA.

        Wrapper function for `belongs_to_rna()`

        |  |  |
        | --- | --- |
        | Parameters: | **atom\_index** (*int*) – An int, the index of the atom to consider. |
        | Returns: | A boolean. True if part of rna, False if not. |

    `coordinate_undo`()¶
    :   Resets the coordinates of all atoms to those saved using the
        set\_coordinate\_undo\_point function.

        Wrapper function for `coordinate_undo()`

    `copy`()¶
    :   Returns an exact copy (scoria.Molecule) of this Molecule object.
        Undo points are NOT copied.

        |  |  |
        | --- | --- |
        | Returns: | A scoria.Molecule, containing to the same atomic information as this scoria.Molecule object. |

    `create_bonds_by_distance`(*remove\_old\_bond\_data=True*, *delete\_excessive\_bonds=True*)¶
    :   Determines which atoms are bound to each other based on their
        proximity.

        Requires the `numpy` and `scipy` libraries.

        Wrapper function for
        `create_bonds_by_distance()`

        |  |  |
        | --- | --- |
        | Parameters: | - **remove\_old\_bond\_data** (*bool*) – An optional boolean, whether or not to   discard old bond data before adding in bonds determined by   distance. True by default. - **delete\_excessive\_bonds** (*bool*) – An optional boolean, whether or not   to check for and delete excessive bonds. True by default. |

    `define_molecule_chain_residue_spherical_boundaries`()¶
    :   Identifies spheres that bound (encompass) the entire molecule, the
        chains, and the residues. This information is stored in
        scoria.Information.Information.hierarchy.

        Requires the `numpy` and `scipy` libraries.

        Wrapper function for
        `define_molecule_chain_residue_spherical_boundaries()`

    `delete_atom`(*index*)¶
    :   Deletes an atom.

        Wrapper function for `delete_atom()`

        |  |  |
        | --- | --- |
        | Parameters: | **index** (*int*) – An int, the index of the atom to delete. |

    `delete_bond`(*index1*, *index2*)¶
    :   Deletes a bond.

        Wrapper function for `delete_bond()`

        |  |  |
        | --- | --- |
        | Parameters: | - **index1** (*int*) – An int, the index of the first atom of the bonded   pair. - **index2** (*int*) – An int, the index of the second atom of the bonded   pair. |

    `delete_trajectory_frame`(*index*)¶
    :   Removes a given frame from the trajectory.

        Wrapper function for `delete_trajectory_frame()`

        |  |  |
        | --- | --- |
        | Parameters: | **index** (*int*) – Integer of the frame to remove. |

    `get_angle_between_three_points`(*pt1*, *pt2*, *pt3*)¶
    :   Computes the angle (in radians) formed by three points (numpy.array
        objects).

        Wrapper function for `get_angle_between_three_points()`

        |  |  |
        | --- | --- |
        | Parameters: | - **pt1** (*numpy.array*) – A numpy.array (x, y, z) representing the first of the   three 3D points. - **pt2** (*numpy.array*) – A numpy.array (x, y, z) representing the second of the   three 3D points. - **pt3** (*numpy.array*) – A numpy.array (x, y, z) representing the third of the   three 3D points. |
        | Returns: | A float containing the angle between the three points, in radians. |

    `get_atom_information`()¶
    :   Retreives the atomic information for the molecule.

        Wrapper function for `get_atom_information()`

        |  |  |
        | --- | --- |
        | Returns: | A masked array containing the atom information. |
        | Return type: | `numpy.ma.MaskedArray` |

        The contents of the array are as follows:

        | member name | dtype | Full Type | Description |
        | --- | --- | --- | --- |
        | record\_name | S6 | six char string | What the atom belongs to |
        | serial | <i8 | 64-bit integer | The index of the atom |
        | name | S5 | five char string | The atom name |
        | resname | S5 | five char string | The residue name |
        | chainid | S1 | one char string | The chain identifier |
        | resseq | <i8 | 64-bit integer | The Residue sequence number |
        | occupancy | <f8 | 64-bit float | Occupancy of atom |
        | tempfactor | <f8 | 64-bit float | Tempature Factor |
        | element | S2 | two char string | The element symbol |
        | charge | S3 | three char string | Charge on the atom |
        | name\_stripped | S5 | five char string | Atom name without space |
        | resname\_stripped | S5 | five char string | Residue name without space |
        | chainid\_stripped | S1 | one char string | Chain identifier without space |
        | element\_stripped | S2 | two char string | Element symbol without space |

        An example for printing the elemental symbols of the first five atoms:

        ```
        >>> atom_info = mol.get_atom_information()
        >>> print(atom_info['element'][0:5])
        ['N' 'C' 'C' 'O' 'C']
        ```

    `get_bonds`()¶
    :   Retreives the bonds beteween atoms as a n x n matrix.

        Wrapper function for `get_bonds()`

        |  |  |
        | --- | --- |
        | Returns: | A binary n x n matrix, where bonds are represented by 1. |
        | Return type: | `numpy.array` |

        An example for finding all atoms bonded with atom 153:

        ```
        >>> bonds = mol.get_bonds()
        >>> for i in range(0,len(bonds)):
        ...     if bonds[153][i] == 1:
        ...             print(153,"-",i)
        153 - 152
        153 - 154
        153 - 155
        ```

    `get_bounding_box`(*selection=None*, *padding=0.0*, *frame=None*)¶
    :   Calculates a box that bounds (encompasses) a set of atoms.

        Wrapper function for `get_bounding_box()`

        |  |  |
        | --- | --- |
        | Parameters: | - **selection** (*numpy.array*) – An optional numpy.array containing the indices of   the atoms to consider. If ommitted, all atoms of the   scoria.Molecule object will be considered. - **padding** (*float*) – An optional float. The bounding box will extend this   many angstroms beyond the atoms being considered. - **frame** (*int*) – An integer indicating at which timestep the center of   mass should be calculated. If ommitted, it defaults to the   first frame of the trajectory. |
        | Returns: | A numpy array representing two 3D points, (min\_x, min\_y, min\_z) and (max\_x, max\_y, max\_z), that bound the molecule. |
        | Return type: | `numpy.array` |

    `get_bounding_sphere`(*selection=None*, *padding=0.0*, *frame=None*)¶
    :   Calculates a sphere that bounds (encompasses) a set of atoms.

        Requires the `numpy` and `scipy` libraries.

        Wrapper function for `get_bounding_sphere()`

        |  |  |
        | --- | --- |
        | Parameters: | - **selection** (*numpy.array*) – An optional numpy.array containing the indices of   the atoms to consider. If ommitted, all atoms of the   scoria.Molecule object will be considered. - **padding** (*float*) – An optional float. The bounding sphere will extend   this many angstroms beyond the atoms being considered. - **frame** (*int*) – An integer indicating at which timestep the center of   mass should be calculated. If ommitted, it defaults to the   first frame of the trajectory. |
        | Returns: | A tuple containing two elements. The first is a numpy.array representing a 3D point, the (x, y, z) center of the sphere. The second is a float, the radius of the sphere. |
        | Return type: | `tuple` (`numpy.array`, `float`) |

    `get_center_of_mass`(*selection=None*, *frame=None*)¶
    :   Determines the center of mass.

        Wrapper function for `get_center_of_mass()`

        |  |  |
        | --- | --- |
        | Parameters: | - **selection** (*numpy.array*) – The indices of   the atoms to consider when calculating the center of mass.   If ommitted, all atoms of the scoria.Molecule object   will be considered. - **frame** (*int*) – The timestep at which the center of mass   should be calculated. If ommitted, it defaults to the first   frame of the trajectory. |
        | Returns: | The x, y, and z coordinates of the center of mass. |
        | Return type: | `numpy.ma.MaskedArray` |

        ```
        >>> mol = scoria.Molecule()
        >>> mol.load_pdb_into("single_frame.pdb")
        >>> print(mol.get_center_of_mass())
        [33.0643089093134 19.135747088722564 16.05629867850796]
        ```

    `get_constants`()¶
    :   Returns a dictionary containing the constants assumed for the molecular model.

        Wrapper function for `get_constants()`

        |  |  |
        | --- | --- |
        | Returns: | The constants assumed by the model. |
        | Return type: | `Dictionary displays` |

        | Dictionary Keys | Value Type | Contains |
        | --- | --- | --- |
        | mass\_dict | dict{str:float} | The mass of elements |
        | rna\_residues | list(str) | RNA residue names |
        | f8\_fields | list(str) | Atom Information floats |
        | vdw\_dict | dict{str:float} | Van der Waals force of elements |
        | i8\_fields | list(str) | Atom Information integers |
        | protein\_residues | list(str) | Protein residue names |
        | bond\_length\_dict | dict{str:float} | Element-pair bond length |
        | element\_names\_with\_two\_letters | list(str) | Element symbols with 2 letters |
        | max\_number\_of\_bonds\_permitted | dict{str:int} | Max bonds per element |
        | dna\_residues | list(str) | DNA reside names |

    `get_coordinates`(*frame=None*)¶
    :   Returns the set of coordinates from the specified frame.

        Wrapper function for `get_coordinates()`

        |  |  |
        | --- | --- |
        | Parameters: | **frame** (*int*) – The timestep from which the coordinates shoule be returned. If ommitted, it defaults to the first frame of the trajectory. |
        | Returns: | The set of coordinates from the specified frame. ``` [[x1, y1, z1], ... [xn, yn, zn]] ``` |
        | Return type: | `numpy.array` |

        ```
        >>> print(mol.get_coordinates())
        [[ -30.85199928  -81.45800018  365.05499268]
         [ -31.99500084  -80.69300079  365.66900635]
         [ -32.0530014   -81.13200378  367.18200684]
         ...,
         [ -27.54199982  -96.25099945  402.83700562]
         [ -23.54199982  -94.7539978   400.41900635]
         [ -22.86100006  -93.72499847  400.55300903]]

        >>> print(mol.get_coordinates(2))
        [[ -28.88899994  -80.45700073  365.51699829]
         [ -30.20000076  -79.73699951  365.99700928]
         [ -30.90699959  -80.5510025   367.13000488]
         ...,
         [ -26.0189991   -97.28099823  403.52600098]
         [ -23.2140007   -94.73999786  400.94699097]
         [ -22.52899933  -93.73300171  400.81399536]]
        ```

    `get_coordinates_undo_point`()¶
    :   NEEDS CLARIFICATION.
        Retreives a previously save set of coordinates to revert to.

        Wrapper function for `get_coordinates_undo_point()`

        |  |  |
        | --- | --- |
        | Returns: | A set of coordinates from which to return to. |
        | Return type: | `numpy.array` or `None` |

    `get_default_trajectory_frame`()¶
    :   Retreives the default trajectory frame index.

        Wrapper function for `get_default_trajectory_frame()`

        |  |  |
        | --- | --- |
        | Returns: | An *int* representing the index of the default trajectory frame. |

    `get_dihedral_angle`(*pt1*, *pt2*, *pt3*, *pt4*)¶
    :   Calculates the dihedral angle formed by four points (numpy.array
        objects).

        Wrapper function for `get_dihedral_angle()`

        |  |  |
        | --- | --- |
        | Parameters: | - **pt1** (*numpy.array*) – A numpy.array (x, y, z) representing the first 3D   point. - **pt2** (*numpy.array*) – A numpy.array (x, y, z) representing the second 3D   point. - **pt3** (*numpy.array*) – A numpy.array (x, y, z) representing the third 3D   point. - **pt4** (*numpy.array*) – A numpy.array (x, y, z) representing the fourth 3D   point. |
        | Returns: | A float containing the dihedral angle between the four points, in radians. |

    `get_distance_to_another_molecules`(*other\_molecules*, *pairwise\_comparison=True*)¶
    :   Computes the minimum distance between any of the atoms of this
        molecular model and any of the atoms of a second specified model.

        Requires the `numpy` and `scipy` libraries.

        Wrapper function for `get_distance_to_another_molecules()`

        |  |  |
        | --- | --- |
        | Parameters: | - **other\_molecules** (*scoria.Molecule*) – a scoria.Molecule, the other molecular   model. - **pairwise\_comparison** (*bool*) – An optional boolean, whether or not to   perform a simple pairwise distance comparison (if True) or   to use a more sophisitcated method (if False). True by   default. |
        | Returns: | A float, the minimum distance between any two atoms of the two specified molecular models (self and other\_molecules). |

    `get_filename`()¶
    :   Returns the filename that the molecule was originally loaded from.

        Wrapper function for `get_filename()`

        |  |  |
        | --- | --- |
        | Returns: | The name of the file. |
        | Return type: | `str` |

        ```
        >>> mol = scoria.Molecule()
        >>> mol.load_pdb_into("single_frame.pdb")
        >>> print(mol.get_filename())
        single_frame.pdb
        ```

    `get_geometric_center`(*selection=None*, *frame=None*)¶
    :   Determines the geometric center of the molecule.

        Wrapper function for `get_geometric_center()`

        |  |  |
        | --- | --- |
        | Parameters: | - **selection** (*numpy.array*) – The indices of   the atoms to consider when calculating the geometric.   If ommitted, all atoms of the scoria.Molecule object   will be considered. - **frame** (*int*) – The timestep at which the geometric center   should be calculated. If ommitted, it defaults to the first   frame of the trajectory. |
        | Returns: | The x, y, and z coordinates of the geometric center. |
        | Return type: | `numpy.array` |

        ```
        >>> mol = scoria.Molecule()
        >>> mol.load_pdb_into("single_frame.pdb")
        >>> print(mol.get_geometric_center())
        [ 33.09860848  19.1221197   16.0426808 ]
        ```

    `get_hierarchy`()¶
    :   NEEDS CLARIFICATION.

        Wrapper function for `get_hierarchy()`

        |  |  |
        | --- | --- |
        | Returns: | A dictionary? |
        | Return type: | `Dictionary displays` |

    `get_index_of_first_bond_partner_of_element`(*atom\_index*, *the\_element*)¶
    :   For a given atom of interest, returns the index of the first
        neighbor of a specified element.

        Wrapper function for `get_index_of_first_bond_partner_of_element()`

        |  |  |
        | --- | --- |
        | Parameters: | - **atom\_index** (*int*) – An int, the index of the atom of interest. - **the\_element** (*str*) – A string specifying the desired element of the   neighbor. |
        | Returns: | An int, the index of the first neighbor atom of the specified element. If no such neighbor exists, returns -1. |
        | Return type: | `int` |

    `get_molecule_from_selection`(*selection*, *serial\_reindex=True*, *resseq\_reindex=False*)¶
    :   Creates a scoria.Molecule from a user-defined atom selection.

        Wrapper function for `get_molecule_from_selection()`

        |  |  |
        | --- | --- |
        | Parameters: | - **selection** (*numpy.array*) – A numpy.array containing the indices of the atoms   in the user-defined selection. - **serial\_reindex** (*bool*) – An optional boolean, whether or not to   reindex the atom serial fields. Default is True. - **resseq\_reindex** (*bool*) – An optional boolean, whether or not to   reindex the atom resseq fields. Default is False. |
        | Returns: | A scoria.Molecule object containing the atoms of the user-defined selection. |

    `get_number_of_bond_partners_of_element`(*atom\_index*, *the\_element*)¶
    :   Counts the number of atoms of a given element bonded to a specified
        atom of interest.

        Requires the `numpy` library.

        Wrapper function for
        `get_number_of_bond_partners_of_element()`

        |  |  |
        | --- | --- |
        | Parameters: | - **atom\_index** (*int*) – An int, the index of the atom of interest. - **the\_element** (*str*) – A string describing the element of the neighbors   to be counted. |
        | Returns: | An int, the number of neighboring atoms of the specified element. |
        | Return type: | `int` |

    `get_other_molecules_aligned_to_this`(*other\_mol*, *tethers*)¶
    :   Aligns a molecule to self (this scoria.Molecule object) using a
        quaternion RMSD alignment.

        Requires the `numpy` library.

        Wrapper function for `get_other_molecules_aligned_to_this()`

        |  |  |
        | --- | --- |
        | Parameters: | - **other\_mol** (*scoria.Molecule*) – A scoria.Molecule that is to be aligned to   this one. - **tethers** (*tuple*) – A tuple of two numpy.array objects, where each array   contains the indices of self and other\_mol, respectively,   such that equivalent atoms are listed in the same order.   So, for example, if (atom 1, self = atom 3, other) and   (atom2, self = atom6, other) than the tethers would be   (numpy.array([1, 2]), numpy.array([3, 6])). |
        | Returns: | The new molecule. |

    `get_planarity_deviation`(*pt1*, *pt2*, *pt3*, *pt4*)¶
    :   Determines how close four points (numpy.array objects) come to lying
        in a common plane.

        Wrapper function for `get_planarity_deviation()`

        |  |  |
        | --- | --- |
        | Parameters: | - **pt1** (*numpy.array*) – A numpy.array (x, y, z) representing a 3D point. - **pt2** (*numpy.array*) – A numpy.array (x, y, z) representing a 3D point. - **pt3** (*numpy.array*) – A numpy.array (x, y, z) representing a 3D point. - **pt4** (*numpy.array*) – A numpy.array (x, y, z) representing a 3D point. |
        | Returns: | A float, the minimum distance between one point and the plane formed by the other three. |

    `get_remarks`()¶
    :   Returns the remarks from the file the molecule was loaded from.

        Wrapper function for `get_remarks()`

        |  |  |
        | --- | --- |
        | Returns: | The remarks from the file an a list of strings. |
        | Return type: | `list` |

        ```
        >>> mol = scoria.Molecule()
        >>> mol.load_pdb_into("single_frame.pdb")
        >>> print(mol.get_remarks())
        [' This is a remark.']
        ```

    `get_rmsd_equivalent_atoms_specified`(*other\_mol*, *tethers*)¶
    :   Calculates the RMSD between this scoria.Molecle object and
        another, where equivalent atoms are explicitly specified.

        Wrapper function for `get_rmsd_equivalent_atoms_specified()`

        |  |  |
        | --- | --- |
        | Parameters: | - **other\_mol** (*scoria.Molecule*) – The other scoria.Molecule object. - **tethers** (*tuple*) – A tuple of two numpy.array objects, where each array   contains the indices of self and other\_mol, respectively,   such that equivalent atoms are listed in the same order.   So, for example, if (atom 1, self = atom 3, other) and   (atom2, self = atom6, other) than the tethers would be   (numpy.array([1, 2]), numpy.array([3, 6])). |
        | Returns: | A float, the RMSD between self and other\_mol. |

    `get_rmsd_heuristic`(*other\_mol*)¶
    :   Caluclates the RMSD between two identical molecules with different
        conformations, per the definition given in “AutoDock Vina: Improving
        the speed and accuracy of docking with a new scoring function,
        efficient optimization, and multithreading,”” by Oleg Trott and Arthur
        J. Olson. Note: Identical means the order of the atoms is the same as
        well.

        Requires the `numpy` library.

        Wrapper function for `get_rmsd_heuristic()`

        |  |  |
        | --- | --- |
        | Parameters: | **other\_mol** (*scoria.Molecule*) – The other scoria.Molecule object. |
        | Returns: | A float, the RMSD between self and other\_mol. |

    `get_rmsd_order_dependent`(*other\_mol*)¶
    :   Calculates the RMSD between two structures, where equivalent atoms
        are listed in the same order.

        Wrapper function for `get_rmsd_order_dependent()`

        |  |  |
        | --- | --- |
        | Parameters: | **other\_mol** (*scoria.Molecule*) – The other scoria.Molecule object. |
        | Returns: | A float, the RMSD between self and other\_mol. |

    `get_total_mass`(*selection=None*)¶
    :   Returns the total mass of all atoms within the molecule, or of a given
        selection.

        Wrapper function for `get_total_mass()`

        |  |  |
        | --- | --- |
        | Parameters: | **selection** (*numpy.array*) – The indices of the atoms to consider when calculating the geometric. If ommitted, all atoms of the scoria.Molecule object will be considered. |
        | Returns: | The total mass of the atom or selection |
        | Return type: | `float` |

        ```
        >>> print(mol.get_total_mass())
        5289.1729999999998
        ```

    `get_total_number_of_atoms`(*selection=None*)¶
    :   Counts the number of atoms.

        Wrapper function for
        `get_total_number_of_atoms()`

        |  |  |
        | --- | --- |
        | Parameters: | - **selection** (*numpy.array*) – An optional numpy.array containing the indices of   the atoms to count. If ommitted, all atoms of the   scoria.Molecule object will be considered. - **frame** (*int*) – An integer indicating at which timestep the center of   mass should be calculated. If ommitted, it defaults to the   first frame of the trajectory. |
        | Returns: | The total number of atoms. |
        | Return type: | `int` |

    `get_total_number_of_heavy_atoms`(*selection=None*)¶
    :   Counts the number of heavy atoms (i.e., atoms that are not
        hydrogens).

        Wrapper function for
        `get_total_number_of_heavy_atoms()`

        |  |  |
        | --- | --- |
        | Parameters: | **selection** (*numpy.array*) – An optional numpy.array containing the indices of the atoms to count. If ommitted, all atoms of the scoria.Molecule object will be considered. |
        | Returns: | The total number of heavy (non-hydrogen) atoms. |
        | Return type: | `int` |

    `get_trajectory_coordinates`()¶
    :   Returns the trajectory for the molecule.

        Wrapper function for `get_trajectory_coordinates()`

        |  |  |
        | --- | --- |
        | Returns: | The set of all coordinates. ``` [[[x11, y11, z11], ... [x1n, y1n, z1n]],  ...,  [[xm1, ym1, zm1], ... [xmn, ymn, zmn]]] ``` |
        | Return type: | `numpy.array` |

        ```
        >>> for coord in mol.get_trajectory_coordinates():
        >>>     print(coord)
        >>>     print()
        [[ -30.85199928  -81.45800018  365.05499268]
         [ -31.99500084  -80.69300079  365.66900635]
         [ -32.0530014   -81.13200378  367.18200684]
         ..., 
         [ -27.54199982  -96.25099945  402.83700562]
         [ -23.54199982  -94.7539978   400.41900635]
         [ -22.86100006  -93.72499847  400.55300903]]

        [[ -30.6779995   -81.32499695  365.73199463]
         [ -31.88100052  -80.38600159  366.0289917 ]
         [ -32.40399933  -80.62799835  367.45700073]
         ..., 
         [ -27.44400024  -96.71099854  402.64700317]
         [ -23.79199982  -94.58899689  400.63598633]
         [ -23.10700035  -93.56300354  400.79598999]]
         <more>
        ```

    `get_trajectory_frame_count`()¶
    :   Returns the number of frames in \_\_trajectory.

        Wrapper function for `get_trajectory_frame_count()`

        |  |  |
        | --- | --- |
        | Returns: | The number of frames in the trajectory. |
        | Return type: | `int` |

    `insert_trajectory_frame`(*index*, *coordinates*)¶
    :   Inserts a new coordinate frame at the end of the trajectory.

        Wrapper function for `insert_trajectory_frame()`

        |  |  |
        | --- | --- |
        | Parameters: | - **coordinates** (*numpy.array*) – A single frame of coordinates to append. - **index** (*int*) – The location where the frame should be added. |

    `invert_selection`(*selection*)¶
    :   Inverts a user-defined selection (i.e., identifies all atoms that
        are not in the seleciton).

        Wrapper function for `invert_selection()`

        |  |  |
        | --- | --- |
        | Parameters: | **selection** (*numpy.array*) – A numpy.array containing the indices of the user-defined selection. |
        | Returns: | A numpy.array containing the indices of all atoms that are not in the user-defined seleciton. |

    `is_planar`(*pt1*, *pt2*, *pt3*, *pt4*, *planarity\_cutoff=0.2*)¶
    :   Checks whether four points (numpy.array) lie in a common plane.

        Wrapper function for `is_planar()`

        |  |  |
        | --- | --- |
        | Parameters: | - **pt1** (*numpy.array*) – A numpy.array (x, y, z) representing a 3D point. - **pt2** (*numpy.array*) – A numpy.array (x, y, z) representing a 3D point. - **pt3** (*numpy.array*) – A numpy.array (x, y, z) representing a 3D point. - **pt4** (*numpy.array*) – A numpy.array (x, y, z) representing a 3D point. - **planarity\_cutoff** (*float*) – An optional float. How much the points can   deviate (in Angstroms) and still be considered planar. The   default is 0.2. |
        | Returns: | A boolean, whether the 4 points can be considered planar. |

    `load_MDAnalysis_into`(*\*args*)¶
    :   Allows import of molecular structure with MDAnalysis

        Requires the `MDAnalysis` library.

        Wrapper function for
        `load_MDAnalysis_into()`

        |  |  |
        | --- | --- |
        | Parameters: | **\*args** – Filename, filenames, or list of file names. Used to inizalize a MDAnalysis.Universe object. |

    `load_MDAnalysis_into_using_universe_object`(*universe*)¶
    :   Allows import of molecular structure with MDAnalysis

        Requires the `MDAnalysis` library.

        Wrapper function for
        `load_MDAnalysis_into_using_universe_object()`

        |  |  |
        | --- | --- |
        | Parameters: | **universe** (*MDAnalysis.core.Universe*) – MDAnalysis Universe object. |

    `load_pdb_into`(*filename*, *bonds\_by\_distance=True*, *serial\_reindex=True*, *resseq\_reindex=False*, *is\_trajectory=False*)¶
    :   Loads the molecular data contained in a pdb file into the current
        scoria.Molecule object.

        Wrapper function for `load_pdb_into()`

        |  |  |
        | --- | --- |
        | Parameters: | - **filename** (*str*) – A string, the filename of the pdb file. - **bonds\_by\_distance** (*bool*) – An optional boolean, whether or not to   determine atomic bonds based on atom proximity. True by   default. - **serial\_reindex** (*bool*) – An optional boolean, whether or not to   reindex the pdb serial field. True by default. - **resseq\_reindex** (*bool*) – An optional boolean, whether or not to   reindex the pdb resseq field. False by default. - **is\_trajectory** (*bool*) – An optional boolean, whether or not the PDB   is multi-frame. |

    `load_pdb_into_using_file_object`(*file\_obj*, *bonds\_by\_distance=True*, *serial\_reindex=True*, *resseq\_reindex=False*, *is\_trajectory=False*)¶
    :   Loads molecular data from a python file object (pdb formatted) into
        the current scoria.Molecule object. Note that most users will want
        to use the load\_pdb\_into() function instead, which is identical except
        that it accepts a filename string instead of a python file object.

        Requires the `numpy` library.

        Wrapper function for `load_pdb_into_using_file_object()`

        |  |  |
        | --- | --- |
        | Parameters: | - **file\_obj** (*file*) – A python file object, containing pdb-formatted   data. - **bonds\_by\_distance** (*bool*) – An optional boolean, whether or not to   determine atomic bonds based on atom proximity. True by   default. - **serial\_reindex** (*bool*) – An optional boolean, whether or not to   reindex the pdb serial field. True by default. - **resseq\_reindex** (*bool*) – An optional boolean, whether or not to   reindex the pdb resseq field. False by default. - **is\_trajectory** (*bool*) – An optional boolean, whether or not the PDB   is multi-frame. |

    `load_pdb_trajectory_into`(*filename*, *bonds\_by\_distance=True*, *serial\_reindex=True*, *resseq\_reindex=False*)¶
    :   Loads the molecular data contained in a pdb trajectory file into the
        current scoria.Molecule object.

        Should be called via the wrapper function `scoria.Molecule.Molecule.load_pdb_trajectory_into()`

        |  |  |
        | --- | --- |
        | Parameters: | - **filename** (*str*) – A string, the filename of the pdb trajectory   file. - **bonds\_by\_distance** (*bool*) – An optional boolean, whether or not to   determine atomic bonds based on atom proximity. True by   default. - **serial\_reindex** (*bool*) – An optional boolean, whether or not to   reindex the pdb serial field. True by default. - **resseq\_reindex** (*bool*) – An optional boolean, whether or not to   reindex the pdb resseq field. False by default. |

    `load_pdb_trajectory_into_using_file_object`(*file\_obj*, *bonds\_by\_distance=True*, *serial\_reindex=True*, *resseq\_reindex=False*)¶
    :   Loads molecular data from a python file object (pdb trajectory
        formatted) into the current scoria.Molecule object. Note that most
        users will want to use the load\_pdb\_trajectory\_into() function
        instead, which is identical except that it accepts a filename string
        instead of a python file object.

        Should be called via the wrapper function `scoria.Molecule.Molecule.load_pdb_trajectory_into_using_file_object()`

        |  |  |
        | --- | --- |
        | Parameters: | - **file\_obj** (*file*) – A python file object, containing pdb-formatted   trajectory data. - **bonds\_by\_distance** (*bool*) – An optional boolean, whether or not to   determine atomic bonds based on atom proximity. True by   default. - **serial\_reindex** (*bool*) – An optional boolean, whether or not to   reindex the pdb serial field. True by default. - **resseq\_reindex** (*bool*) – An optional boolean, whether or not to   reindex the pdb resseq field. False by default. |

    `load_pdbqt_into`(*filename*, *bonds\_by\_distance=False*, *serial\_reindex=True*, *resseq\_reindex=False*, *is\_trajectory=False*)¶
    :   Loads the molecular data contained in a pdbqt file into the current
        scoria.Molecule object. Note that this implementation is
        incomplete. It doesn’t save atomic charges, for example. The atom
        types are stored in the “element\_padded” and “element” columns.

        Wrapper function for `load_pdbqt_into()`

        |  |  |
        | --- | --- |
        | Parameters: | - **filename** (*str*) – A string, the filename of the pdbqt file. - **bonds\_by\_distance** (*bool*) – An optional boolean, whether or not to   determine atomic bonds based on atom proximity. False by   default, unlike for PDB. - **serial\_reindex** (*bool*) – An optional boolean, whether or not to   reindex the pdb serial field. True by default. - **resseq\_reindex** (*bool*) – An optional boolean, whether or not to   reindex the pdbqt resseq field. False by default. - **is\_trajectory** (*bool*) – An optional boolean, whether or not the PDB   is multi-frame. Defaults of False. |

    `load_pdbqt_into_using_file_object`(*file\_obj*, *bonds\_by\_distance=False*, *serial\_reindex=True*, *resseq\_reindex=False*, *is\_trajectory=False*)¶
    :   Loads molecular data from a python file object (pdbqt formatted)
        into the current scoria.Molecule object. Note that most users will
        want to use the load\_pdb\_into() function instead, which is identical
        except that it accepts a filename string instead of a python file
        object.

        Requires the `numpy` library.

        Wrapper function for `load_pdbqt_into_using_file_object()`

        |  |  |
        | --- | --- |
        | Parameters: | - **file\_obj** (*file*) – A python file object, containing pdb-formatted   data. - **bonds\_by\_distance** (*bool*) – An optional boolean, whether or not to   determine atomic bonds based on atom proximity. False by   default, unlike for PDB. - **serial\_reindex** (*bool*) – An optional boolean, whether or not to   reindex the pdb serial field. True by default. - **resseq\_reindex** (*bool*) – An optional boolean, whether or not to   reindex the pdb resseq field. False by default. - **is\_trajectory** (*bool*) – An optional boolean, whether or not the PDB   is multi-frame. Defaults of False. |

    `load_pdbqt_trajectory_into`(*filename*, *bonds\_by\_distance=True*, *serial\_reindex=True*, *resseq\_reindex=False*)¶
    :   Loads the molecular data contained in a pdbqt trajectoy file (e.g., an
        AutoDock Vina output file) into the current scoria.Molecule
        object.

        Should be called via the wrapper function `scoria.Molecule.Molecule.load_pdbqt_trajectory_into()`

        |  |  |
        | --- | --- |
        | Parameters: | - **filename** (*str*) – A string, the filename of the pdbqt file. - **bonds\_by\_distance** (*bool*) – An optional boolean, whether or not to   determine atomic bonds based on atom proximity. True by   default. - **serial\_reindex** (*bool*) – An optional boolean, whether or not to   reindex the pdb serial field. True by default. - **resseq\_reindex** (*bool*) – An optional boolean, whether or not to   reindex the pdb resseq field. False by default. |

    `load_pdbqt_trajectory_into_using_file_object`(*file\_obj*, *bonds\_by\_distance=True*, *serial\_reindex=True*, *resseq\_reindex=False*)¶
    :   Loads molecular data from a python file object (pdbqt trajectory
        formatted) into the current scoria.Molecule object. Note that most
        users will want to use the load\_pdbqt\_trajectory\_into() function
        instead, which is identical except that it accepts a filename string
        instead of a python file object.

        Wrapper function for
        `load_pdbqt_trajectory_into_using_file_object()`

        |  |  |
        | --- | --- |
        | Parameters: | - **file\_obj** (*file*) – A python file object, containing pdbqt-formatted   trajectory data. - **bonds\_by\_distance** (*bool*) – An optional boolean, whether or not to   determine atomic bonds based on atom proximity. True by   default. - **serial\_reindex** (*bool*) – An optional boolean, whether or not to   reindex the pdb serial field. True by default. - **resseq\_reindex** (*bool*) – An optional boolean, whether or not to   reindex the pdb resseq field. False by default. |

    `load_pym_into`(*filename*)¶
    :   Loads the molecular data contained in a pym file into the current
        scoria.Molecule object.

        Requires the `numpy` library.

        Wrapper function for `load_pym_into()`

        |  |  |
        | --- | --- |
        | Parameters: | **filename** (*str*) – A string, the filename of the pym file. |

    `merge_with_another_molecules`(*other\_molecules*)¶
    :   Merges two molecular models into a single model.

        Wrapper function for `merge_with_another_molecules()`

        |  |  |
        | --- | --- |
        | Parameters: | **other\_molecules** (*scoria.Molecule*) – A molecular model (scoria.Molecule object). |
        | Returns: | A single scoria.Molecule object containing the atoms of this model combined with the atoms of other\_molecules. |

    `numpy_structured_array_remove_field`(*narray*, *field\_names*)¶
    :   Removes a specific field name from a structured numpy array.

        |  |  |
        | --- | --- |
        | Parameters: | - **narray** (*numpy.array*) – A structured numpy array. - **field\_names** (*list(str)*) – A list of strings, where each string is one of   the field names of narray. |
        | Returns: | A structured numpy array identical to narray, but with the field names in field\_names removed. |

    `resseq_reindex`()¶
    :   Reindexes the resseq field of the atoms in the molecule, starting
        with 1.

        Wrapper function for `resseq_reindex()`

    `rotate_molecule_around_a_line_between_atoms`(*line\_point1\_index*, *line\_point2\_index*, *rotate*)¶
    :   Rotate the molecular model about a line segment. The end points of
        the line segment are atoms of specified indices.

        Wrapper function for `rotate_molecule_around_a_line_between_atoms()`

        |  |  |
        | --- | --- |
        | Parameters: | - **line\_point1\_index** (*int*) – An int, the index of the first atom at one   end of the line segment. - **line\_point2\_index** (*int*) – An int, the index of the second atom at   the other end of the line segment. - **rotate** (*float*) – A float, the angle of rotation, in radians. |

    `rotate_molecule_around_a_line_between_points`(*line\_point1*, *line\_point2*, *rotate*)¶
    :   Rotate the molecular model about a line segment. The end points of
        the line segment are explicitly specified coordinates.

        Wrapper function for `rotate_molecule_around_a_line_between_points()`

        |  |  |
        | --- | --- |
        | Parameters: | - **line\_point1** (*numpy.array*) – A numpy.array (x, y, z) corresponding to one end   of the line segment. - **line\_point2** (*numpy.array*) – A numpy.array (x, y, z) corresponding to the   other end of the line segment. - **rotate** (*float*) – A float, the angle of rotation, in radians. |

    `rotate_molecule_around_pivot_atom`(*pivot\_index*, *thetax*, *thetay*, *thetaz*)¶
    :   Rotate the molecular model around a specified atom.

        Requires the `numpy` library.

        Wrapper function for `rotate_molecule_around_pivot_atom()`

        |  |  |
        | --- | --- |
        | Parameters: | - **pivot\_index** (*int*) – An int, the index of the atom about which the   molecular model will be rotated. - **thetax** (*float*) – A float, the angle to rotate relative to the x axis,   in radians. - **thetay** (*float*) – A float, the angle to rotate relative to the y axis,   in radians. - **thetaz** (*float*) – A float, the angle to rotate relative to the z axis,   in radians. |

    `rotate_molecule_around_pivot_point`(*pivot*, *thetax*, *thetay*, *thetaz*)¶
    :   Rotate the molecular model around a specified atom.

        Requires the `numpy` library.

        Wrapper function for `rotate_molecule_around_pivot_point()`

        |  |  |
        | --- | --- |
        | Parameters: | - **pivot** (*numpy.array*) – A numpy.array, the (x, y, z) coordinate about which   the molecular model will be rotated. - **thetax** (*float*) – A float, the angle to rotate relative to the x axis,   in radians. - **thetay** (*float*) – A float, the angle to rotate relative to the y axis,   in radians. - **thetaz** (*float*) – A float, the angle to rotate relative to the z axis,   in radians. |

    `save_pdb`(*filename=''*, *serial\_reindex=True*, *resseq\_reindex=False*, *return\_text=False*, *frame=None*)¶
    :   Saves the molecular data contained in a scoria.Molecule object
        to a pdb file.

        Wrapper function for `save_pdb()`

        |  |  |
        | --- | --- |
        | Parameters: | - **filename** (*str*) – An string, the filename to use for saving. - **serial\_reindex** (*bool*) – An optional boolean, whether or not to   reindex the pdb serial field. True by default. - **resseq\_reindex** (*bool*) – An optional boolean, whether or not to   reindex the pdb resseq field. False by default. - **return\_text** (*bool*) – An optional boolean, whether or not to return   text instead of writing to a file. If True, the filename   variable is ignored. - **frame** (*int*) – If specified, a single-frame PDB will be generated.   If not specified, a multi-frame PDB will be generated if   the Molecule has multiple frames. Otherwise, the single   existing frame will be used. |
        | Returns: | If return\_text is True, a PDB-formatted string. Otherwise, returns nothing. |
        | Return type: | `str` or `None` |

    `save_pym`(*filename*, *save\_bonds=False*, *save\_filename=False*, *save\_remarks=False*, *save\_hierarchy=False*, *save\_coordinates\_undo\_point=False*)¶
    :   Saves the molecular data contained in a scoria.Molecule object
        to a pym file.

        Requires the `numpy` library.

        Wrapper function for `save_pym()`

        |  |  |
        | --- | --- |
        | Parameters: | - **filename** (*str*) – An string, the filename to use for saving. (Note   that this is actually a directory, not a file.) - **save\_bonds** (*bool*) – An optional boolean, whether or not to save   information about atomic bonds. False by default. - **save\_filename** (*bool*) – An optional boolean, whether or not to save   the original (pdb) filename. False by default. - **save\_remarks** (*bool*) – An optional boolean, whether or not to save   remarks associated with the molecule. False by default. - **save\_hierarchy** (*bool*) – An optional boolean, whether or not to save   information about spheres the bound (encompass) the whole   molecule, the chains, and the residues. False by default. - **save\_coordinates\_undo\_point** (*bool*) – An optional boolean, whether or   not to save the last coordinate undo point. False by   default. |

    `select_all`()¶
    :   Selects all the atoms in a scoria.Molecule object.

        Wrapper function for `select_all()`

        |  |  |
        | --- | --- |
        | Returns: | A numpy.array containing the indices of all atoms in the scoria.Molecule object. |

    `select_all_atoms_bound_to_selection`(*selections*)¶
    :   Selects all the atoms that are bound to a user-specified selection.

        Requires the `numpy` library.

        Wrapper function for `select_all_atoms_bound_to_selection()`

        |  |  |
        | --- | --- |
        | Parameters: | **selection** (*numpy.array*) – A numpy.array containing the indices of the user-specified selection. |
        | Returns: | A numpy.array containing the indices of the atoms that are bound to the user-specified selection. Note that this new selection does not necessarily include the indices of the original user-specified selection. |

    `select_atoms`(*selection\_criteria*)¶
    :   Select a set of atoms based on user-specified criteria.

        Wrapper function for `select_atoms()`

        |  |  |
        | --- | --- |
        | Parameters: | **selection\_criteria** (*dict*) – A dictionary, where the keys correspond to keys in the self.\_\_parent\_Information.Information.get\_atom\_information() structured numpy array, and the values are lists of acceptable matches. The selection is a logical “AND” between dictionary entries, but “OR” within the value lists themselves. For example: {‘atom’:[‘CA’, ‘O’], ‘chain’:’A’, ‘resname’:’PRO’} would select all atoms with the names CA or O that are located in the PRO residues of chain A. |
        | Returns: | A numpy.array containing the indices of the atoms of the selection. |

    `select_atoms_from_same_molecule`(*selection*)¶
    :   Selects all the atoms that belong to the same molecule as a
        user-defined selection, assuming that the scoria.Molecule object
        actually contains multiple physically distinct molecules that are not
        bound to each other via covalent bonds.

        Requires the `numpy` library.

        Wrapper function for `select_atoms_from_same_molecule()`

        |  |  |
        | --- | --- |
        | Parameters: | **selection** (*numpy.array*) – A numpy.array containing the indices of the user-defined selection. |
        | Returns: | A numpy.array containing the indices of the atoms belonging to the same molecules as the atoms of the user-defined selection. |

    `select_atoms_in_bounding_box`(*bounding\_box*)¶
    :   Selects all the atoms that are within a bounding box.

        Requires the `numpy` library.

        Wrapper function for `select_atoms_in_bounding_box()`

        |  |  |
        | --- | --- |
        | Parameters: | **bounding\_box** (*numpy.array*) – A 2x3 numpy.array containing the minimum and maximum points of the bounding box. Example: numpy.array( [[min\_x, min\_y, min\_z], [max\_x, max\_y, max\_z]] ). |
        | Returns: | A numpy.array containing the indices of the atoms that are within the bounding box. |

    `select_atoms_in_same_residue`(*selection*)¶
    :   Selects all atoms that are in the same residue as any of the atoms
        of a user-defined seleciton. Residues are considered unique if they
        have a unique combination of resname, resseq, and chainid fields.

        Wrapper function for `select_atoms_in_same_residue()`

        |  |  |
        | --- | --- |
        | Parameters: | **selection** (*numpy.array*) – A numpy.array containing the indices of the user-defined selection. |
        | Returns: | A numpy.array containing the indices of all atoms in the same residue as any of the atoms of the user-defined selection. |

    `select_atoms_near_other_selection`(*selection*, *cutoff*)¶
    :   Selects all atoms that are near the atoms of a user-defined
        selection.

        Requires the `numpy` and `scipy` libraries.

        Wrapper function for `select_atoms_near_other_selection()`

        |  |  |
        | --- | --- |
        | Parameters: | - **selection** (*numpy.array*) – A numpy.array containing the indices of the   user-defined selection. - **cutoff** (*float*) – A float, the distance cutoff (in Angstroms). |
        | Returns: | A numpy.array containing the indices of all atoms near the user-defined selection, not including the atoms of the user-defined selection themselves. |

    `select_branch`(*root\_atom\_index*, *directionality\_atom\_index*)¶
    :   Identify an isolated “branch” of a molecular model. Assumes the
        atoms with indices root\_atom\_index and directionality\_atom\_index are
        bound to one another and that the branch starts at root\_atom\_index one
        and “points” in the direction of directionality\_atom\_index.

        Requires the `numpy` library.

        Wrapper function for `select_branch()`

        |  |  |
        | --- | --- |
        | Parameters: | - **root\_atom\_index** (*int*) – An int, the index of the first atom in the   branch (the “root”). - **directionality\_atom\_index** (*int*) – An int, the index of the second atom   in the branch, used to establish directionality |
        | Returns: | A numpy array containing the indices of the atoms of the branch. |

    `select_close_atoms_from_different_molecules`(*other\_mol*, *cutoff*, *pairwise\_comparison=True*, *terminate\_early=False*)¶
    :   Effectively detects steric clashes between self and another
        scoria.Molecule.

        Requires the `numpy` and `scipy` libraries.

        Wrapper function for `select_close_atoms_from_different_molecules()`

        |  |  |
        | --- | --- |
        | Parameters: | - **other\_mol** (*scoria.Molecule*) – A scoria.Molecule object of the other   molecule. - **cutoff** (*float*) – A float, the user-defined distance cutoff in   Angstroms. - **pairwise\_comparison** (*bool*) – An optional boolean, whether or not to   perform a simple pairwise distance comparison (if True) or   to use a more sophisitcated method (if False). True by   default. - **terminate\_early** (*bool*) – An optional boolean, whether or not to stop   looking for steric clashes once one is found. False by   default. |
        | Returns: | A tuple containing two elements. The first is a numpy.array containing the indices of all nearby atoms from this scoria.Molecule object (self). The second is a numpy.array containing the indices of all nearby atoms from the other molecule. |

    `selections_of_chains`()¶
    :   Identifies the atom selections of each chain.

        Requires the `numpy` library.

        Wrapper function for `selections_of_chains()`

        |  |  |
        | --- | --- |
        | Returns: | A dictionary. The keys of the dictionary correspond to the chainids, and the values are numpy.array objects containing the indices of the associated chain atoms. |

    `selections_of_constituent_molecules`()¶
    :   Identifies the indices of atoms belonging to separate molecules,
        assuming that the scoria.Molecule object actually contains multiple
        physically distinct molecules that are not bound to each other via
        covalent bonds.

        Requires the `numpy` library.

        Wrapper function for `selections_of_constituent_molecules()`

        |  |  |
        | --- | --- |
        | Returns: | A python list of numpy.array objects containing the indices of the atoms belonging to each molecule of the composite scoria.Molecule object. |

    `selections_of_residues`()¶
    :   Identifies the atom selections of each residue.

        Requires the `numpy` library.

        Wrapper function for `selections_of_residues()`

        |  |  |
        | --- | --- |
        | Returns: | A dictionary. The keys of this dictionary correspond to the unique resname-resseq-chainid residue identifiers, and the values are numpy.array objects containing the indices of the associated residue atoms. |

    `serial_reindex`()¶
    :   Reindexes the serial field of the atoms in the molecule, starting
        with 1.

        Wrapper function for `serial_reindex()`

    `set_atom_information`(*atom\_information*)¶
    :   Sets the \_\_atom\_information variable. See
        `get_atom_information()` for
        information on the numpy.array structure.

        Wrapper function for `set_atom_information()`

        |  |  |
        | --- | --- |
        | Parameters: | **atom\_information** (*numpy.array*) – An array containing details on the constituent atoms. |

    `set_atom_location`(*atom\_index*, *new\_location*)¶
    :   Translates the entire molecular model (without rotating) so that the
        atom with the specified index is located at the specified coordinate.

        Wrapper function for `set_atom_location()`

        |  |  |
        | --- | --- |
        | Parameters: | - **atom\_index** (*int*) – An int, the index of the target atom. - **new\_location** (*numpy.array*) – A numpy.array specifying the new (x, y, z)   coordinate of the specified atom. |
        | Returns: | A numpy.array specifying the (delta\_x, delta\_y, delta\_z) vector by which the pmolecule.Molecule was translated. |

    `set_bonds`(*bonds*)¶
    :   Sets the \_\_bonds variable. See
        `get_bonds()` for additional
        information.

        Wrapper function for `set_bonds()`

        |  |  |
        | --- | --- |
        | Parameters: | **bonds** (*numpy.array*) – A binary n x n matrix containing bonding information. |

    `set_coordinate_undo_point`()¶
    :   Sets (“saves”) the undo point of the atom coordinates. Any
        subsequent manipulations of atomic coordinates can be “undone” by
        reseting to this configuration via the coordinate\_undo function.

        Wrapper function for `set_coordinate_undo_point()`

    `set_coordinates`(*coordinates*, *frame=None*)¶
    :   Sets a specified frame of the \_\_trajectory variable.

        Wrapper function for `set_coordinates()`

        |  |  |
        | --- | --- |
        | Parameters: | - **coordinates** (*numpy.array*) – An array of atomic coordinates. - **frame** (*int*) – An integer represeting the frame of the trajectory to be modified |

    `set_coordinates_undo_point`(*coordinates\_undo\_point*)¶
    :   Sets the \_\_coordinates\_undo\_point variable.

        Wrapper function for `set_coordinates_undo_point()`

        |  |  |
        | --- | --- |
        | Parameters: | **coordinates\_undo\_point** (*numpy.array*) – A coordinate set to revert to after modification. |

    `set_default_trajectory_frame`(*frame*)¶
    :   Set’s the default trajectory frame for various calculations.

        Wrapper function for `set_default_trajectory_frame()`

        |  |  |
        | --- | --- |
        | Parameters: | **frame** (*int*) – The default frame for coordinate selection. |

    `set_filename`(*filename*)¶
    :   Sets the \_\_filename variable. Note: this does not reload or modify the
        molecule in anyway.

        Wrapper function for `set_filename()`

        |  |  |
        | --- | --- |
        | Parameters: | **filename** (*str*) – String representation of the filename. |

    `set_hierarchy`(*hierarchy*)¶
    :   DEPRECIATED?

        Wrapper function for `set_hierarchy()`

    `set_remarks`(*remarks*)¶
    :   Sets the \_\_remarks variable.

        Wrapper function for `set_remarks()`

        |  |  |
        | --- | --- |
        | Parameters: | **remarks** (*list(str)*) – List containing remarks. |

    `set_trajectory_coordinates`(*trajectory*)¶
    :   Sets the \_\_trajectory variable.

        Wrapper function for `set_trajectory_coordinates()`

        |  |  |
        | --- | --- |
        | Parameters: | **trajectory** (*numpy.array*) – An array of atomic coordinates. |

    `steric_clash_with_another_molecules`(*other\_mol*, *cutoff*, *pairwise\_comparison=True*)¶
    :   Detects steric clashes between the scoria.Molecule (self) and
        another scoria.Molecule.

        Requires the `numpy` and `scipy` libraries.

        Wrapper function for `steric_clash_with_another_molecules()`

        |  |  |
        | --- | --- |
        | Parameters: | - **other\_mol** (*scoria.Molecule*) – The scoria.Molecule object that will be   evaluated for steric clashes. - **cutoff** (*float*) – A float, the user-defined distance cutoff in   Angstroms. - **pairwise\_comparison** (*bool*) – An optional boolean, whether or not to   perform a simple pairwise distance comparison (if True) or   to use a more sophisitcated method (if False). True by   default. |
        | Returns: | A boolean. True if steric clashes are present, False if they are not. |

    `translate_molecule`(*delta*)¶
    :   Translate all the atoms of the molecular model by a specified
        vector.

        Wrapper function for `translate_molecule()`

        |  |  |
        | --- | --- |
        | Parameters: | **delta** (*numpy.array*) – A numpy.array (delta\_x, delta\_y, delta\_z) specifying the amount to move each atom along the x, y, and z coordinates. |

### Table Of Contents

- 1. The Molecule Class
  - 1.1. Initiating and using the object
  - 1.2. Function Definitions

#### Previous topic

Welcome to Scoria’s documentation!

#### Next topic

2. Scoria Demo

### This Page

- Show Source

### Quick search

### Navigation

- index
- modules |
- next |
- previous |
- scoria 2.0 documentation »

© Copyright 2016, Jacob Durrant.
Created using Sphinx 1.4.6.
