# Supplementary material for: Scoria: a Python module for manipulating 3D molecular data
Source: J Cheminform. 2017 Sep 18;9:52. doi: 10.1186/s13321-017-0237-8 (PMC5603467; doi:10.1186/s13321-017-0237-8)
Supplement: Supplementary file 2 — Additional file 2. An archived version of Scoria, without MDAnalysis support. [file 13321_2017_237_MOESM2_ESM.zip › scoria-1.0.0/docs/build/html/OtherMolecules.html]

8. The OtherMolecules class — scoria 2.0 documentation


### Navigation

- index
- modules |
- next |
- previous |
- scoria 2.0 documentation »

# 8. The OtherMolecules class¶

## 8.1. Using the OtherMolecules functions¶

The functions within OtherMolecules allow the Molecule class to interact with
other Molecules objects. The major functions are to compare coordinate sets
and add other molecules.

## 8.2. Function Definitions¶

*class* `scoria.OtherMolecules.``OtherMolecules`(*parent\_molecule\_object*)¶
:   A class for characterizing the relationships between multiple
    scoria.Molecule objects.

    `get_distance_to_another_molecules`(*other\_molecules*, *pairwise\_comparison=True*)¶
    :   Computes the minimum distance between any of the atoms of this
        molecular model and any of the atoms of a second specified model.

        Requires the `numpy` and `scipy` libraries.

        Wrapper function for `get_distance_to_another_molecules()`

        |  |  |
        | --- | --- |
        | Parameters: | - **other\_molecules** (*scoria.Molecule*) – a scoria.Molecule, the other molecular   model. - **pairwise\_comparison** (*bool*) – An optional boolean, whether or not to   perform a simple pairwise distance comparison (if True) or   to use a more sophisitcated method (if False). True by   default. |
        | Returns: | A float, the minimum distance between any two atoms of the two specified molecular models (self and other\_molecules). |

    `get_other_molecules_aligned_to_this`(*other\_mol*, *tethers*, *weight\_mat=None*)¶
    :   Aligns a molecule to self (this scoria.Molecule object) using a
        quaternion RMSD alignment.

        Requires the `numpy` library.

        Wrapper function for `get_other_molecules_aligned_to_this()`

        |  |  |
        | --- | --- |
        | Parameters: | - **other\_mol** (*scoria.Molecule*) – A scoria.Molecule that is to be aligned to   this one. - **tethers** (*list[list]*) – A list of lists, where the inner list is   (tether1\_index, tether2\_index). That inner list can   also be a numpy array. Each inner array contains the   indices of self and other\_mol, respectively, such   that equivalent atoms are listed in the same order.   So, for example, if (atom 1, self = atom 3, other)   and (atom2, self = atom6, other) than the tethers   would be (numpy.array([1, 2]), numpy.array([3, 6])),   or [(1, 2), (3, 6)]. |
        | Returns: | The new molecule. |

    `get_rmsd_equivalent_atoms_specified`(*other\_mol*, *tethers*)¶
    :   Calculates the RMSD between this scoria.Molecle object and
        another, where equivalent atoms are explicitly specified.

        Wrapper function for `get_rmsd_equivalent_atoms_specified()`

        |  |  |
        | --- | --- |
        | Parameters: | - **other\_mol** (*scoria.Molecule*) – The other scoria.Molecule object. - **tethers** (*tuple*) – A tuple of two numpy.array objects, where each array   contains the indices of self and other\_mol, respectively,   such that equivalent atoms are listed in the same order.   So, for example, if (atom 1, self = atom 3, other) and   (atom2, self = atom6, other) than the tethers would be   (numpy.array([1, 2]), numpy.array([3, 6])). |
        | Returns: | A float, the RMSD between self and other\_mol. |

    `get_rmsd_heuristic`(*other\_mol*)¶
    :   Caluclates the RMSD between two identical molecules with different
        conformations, per the definition given in “AutoDock Vina: Improving
        the speed and accuracy of docking with a new scoring function,
        efficient optimization, and multithreading,”” by Oleg Trott and Arthur
        J. Olson. Note: Identical means the order of the atoms is the same as
        well.

        Requires the `numpy` and `scipy` libraries.

        Wrapper function for `get_rmsd_heuristic()`

        |  |  |
        | --- | --- |
        | Parameters: | **other\_mol** (*scoria.Molecule*) – The other scoria.Molecule object. |
        | Returns: | A float, the RMSD between self and other\_mol. |

    `get_rmsd_order_dependent`(*other\_mol*)¶
    :   Calculates the RMSD between two structures, where equivalent atoms
        are listed in the same order.

        Wrapper function for `get_rmsd_order_dependent()`

        |  |  |
        | --- | --- |
        | Parameters: | **other\_mol** (*scoria.Molecule*) – The other scoria.Molecule object. |
        | Returns: | A float, the RMSD between self and other\_mol. |

    `merge_with_another_molecules`(*other\_molecules*)¶
    :   Merges two molecular models into a single model.

        Wrapper function for `merge_with_another_molecules()`

        |  |  |
        | --- | --- |
        | Parameters: | **other\_molecules** (*scoria.Molecule*) – A molecular model (scoria.Molecule object). |
        | Returns: | A single scoria.Molecule object containing the atoms of this model combined with the atoms of other\_molecules. |

    `steric_clash_with_another_molecules`(*other\_mol*, *cutoff*, *pairwise\_comparison=True*)¶
    :   Detects steric clashes between the scoria.Molecule (self) and
        another scoria.Molecule.

        Requires the `numpy` and `scipy` libraries.

        Wrapper function for `steric_clash_with_another_molecules()`

        |  |  |
        | --- | --- |
        | Parameters: | - **other\_mol** (*scoria.Molecule*) – The scoria.Molecule object that will be   evaluated for steric clashes. - **cutoff** (*float*) – A float, the user-defined distance cutoff in   Angstroms. - **pairwise\_comparison** (*bool*) – An optional boolean, whether or not to   perform a simple pairwise distance comparison (if True) or   to use a more sophisitcated method (if False). True by   default. |
        | Returns: | A boolean. True if steric clashes are present, False if they are not. |

### Table Of Contents

- 8. The OtherMolecules class
  - 8.1. Using the OtherMolecules functions
  - 8.2. Function Definitions

#### Previous topic

7. The Manipulation class

#### Next topic

9. The Quaternion object

### This Page

- Show Source

### Quick search

### Navigation

- index
- modules |
- next |
- previous |
- scoria 2.0 documentation »

© Copyright 2016, Jacob Durrant.
Created using Sphinx 1.4.6.
