# Supplementary material for: Scoria: a Python module for manipulating 3D molecular data
Source: J Cheminform. 2017 Sep 18;9:52. doi: 10.1186/s13321-017-0237-8 (PMC5603467; doi:10.1186/s13321-017-0237-8)
Supplement: Supplementary file 2 — Additional file 2. An archived version of Scoria, without MDAnalysis support. [file 13321_2017_237_MOESM2_ESM.zip › scoria-1.0.0/docs/build/html/Selections.html]

10. The Selections class — scoria 2.0 documentation


### Navigation

- index
- modules |
- previous |
- scoria 2.0 documentation »

# 10. The Selections class¶

## 10.1. Using the Selection functions¶

Functions within the Selection module search the molecule given specific
criteria and return a list of indexes that match that criteria.

## 10.2. Function Definitions¶

*class* `scoria.Selections.``Selections`(*parent\_molecule\_object*)¶
:   A class for selecting atoms. Subclass to the
    `scoria.Molecule` class.

    `get_molecule_from_selection`(*selection*, *serial\_reindex=True*, *resseq\_reindex=False*)¶
    :   Creates a scoria.Molecule from a user-defined atom selection.

        Should be called via the wrapper function `get_molecule_from_selection()`.

        |  |  |
        | --- | --- |
        | Parameters: | - **selection** (*numpy.array*) – A numpy.array containing the indices of the atoms   in the user-defined selection. - **serial\_reindex** (*bool*) – An optional boolean, whether or not to   reindex the atom serial fields. Default is True. - **resseq\_reindex** (*bool*) – An optional boolean, whether or not to   reindex the atom resseq fields. Default is False. |
        | Returns: | A scoria.Molecule object containing the atoms of the user-defined selection. |

    `invert_selection`(*selection*)¶
    :   Inverts a user-defined selection (i.e., identifies all atoms that
        are not in the seleciton).

        Should be called via the wrapper function
        `invert_selection()`.

        |  |  |
        | --- | --- |
        | Parameters: | **selection** (*numpy.array*) – A numpy.array containing the indices of the user-defined selection. |
        | Returns: | A numpy.array containing the indices of all atoms that are not in the user-defined seleciton. |

    `select_all`()¶
    :   Selects all the atoms in a scoria.Molecule object.

        Should be called via the wrapper function `select_all()`.

        |  |  |
        | --- | --- |
        | Returns: | A numpy.array containing the indices of all atoms in the scoria.Molecule object. |

    `select_all_atoms_bound_to_selection`(*selection*)¶
    :   Selects all the atoms that are bound to a user-specified selection.

        Requires the `numpy` library.

        Should be called via the wrapper function
        `select_all_atoms_bound_to_selection()`.

        |  |  |
        | --- | --- |
        | Parameters: | **selection** (*numpy.array*) – A numpy.array containing the indices of the user-specified selection. |
        | Returns: | A numpy.array containing the indices of the atoms that are bound to the user-specified selection. Note that this new selection does not necessarily include the indices of the original user-specified selection. |

    `select_atoms`(*selection\_criteria*)¶
    :   Select a set of atoms based on user-specified criteria.

        Should be called via the wrapper function `select_atoms()`.

        |  |  |
        | --- | --- |
        | Parameters: | **selection\_criteria** (*dict*) – A dictionary, where the keys correspond to keys in the self.\_\_parent\_Information.Information.get\_atom\_information() structured numpy array, and the values are lists of acceptable matches. The selection is a logical “AND” between dictionary entries, but “OR” within the value lists themselves. For example: {‘atom’:[‘CA’, ‘O’], ‘chain’:’A’, ‘resname\_padded’:’PRO’} would select all atoms with the names CA or O that are located in the PRO residues of chain A. |
        | Returns: | A numpy.array containing the indices of the atoms of the selection. |

    `select_atoms_from_same_molecule`(*selection*)¶
    :   Selects all the atoms that belong to the same molecule as a
        user-defined selection, assuming that the scoria.Molecule object
        actually contains multiple physically distinct molecules that are not
        bound to each other via covalent bonds.

        Requires the `numpy` library.

        Should be called via the wrapper function
        `select_atoms_from_same_molecule()`.

        |  |  |
        | --- | --- |
        | Parameters: | **selection** (*numpy.array*) – A numpy.array containing the indices of the user-defined selection. |
        | Returns: | A numpy.array containing the indices of the atoms belonging to the same molecules as the atoms of the user-defined selection. |

    `select_atoms_in_bounding_box`(*bounding\_box*)¶
    :   Selects all the atoms that are within a bounding box.

        Requires the `numpy` library.

        Should be called via the wrapper function
        `select_atoms_in_bounding_box()`.

        |  |  |
        | --- | --- |
        | Parameters: | **bounding\_box** (*numpy.array*) – A 2x3 numpy.array containing the minimum and maximum points of the bounding box. Example: numpy.array( [[min\_x, min\_y, min\_z], [max\_x, max\_y, max\_z]] ). |
        | Returns: | A numpy.array containing the indices of the atoms that are within the bounding box. |

    `select_atoms_in_same_residue`(*selection*)¶
    :   Selects all atoms that are in the same residue as any of the atoms
        of a user-defined seleciton. Residues are considered unique if they
        have a unique combination of resname, resseq, and chainid fields.

        Should be called via the wrapper function
        `select_atoms_in_same_residue()`.

        |  |  |
        | --- | --- |
        | Parameters: | **selection** (*numpy.array*) – A numpy.array containing the indices of the user-defined selection. |
        | Returns: | A numpy.array containing the indices of all atoms in the same residue as any of the atoms of the user-defined selection. |

    `select_atoms_near_other_selection`(*selection*, *cutoff*)¶
    :   Selects all atoms that are near the atoms of a user-defined
        selection.

        Requires the `numpy` and `scipy` libraries.

        Should be called via the wrapper function
        `select_atoms_near_other_selection()`.

        |  |  |
        | --- | --- |
        | Parameters: | - **selection** (*numpy.array*) – A numpy.array containing the indices of the   user-defined selection. - **cutoff** (*float*) – A float, the distance cutoff (in Angstroms). |
        | Returns: | A numpy.array containing the indices of all atoms near the user-defined selection, not including the atoms of the user-defined selection themselves. |

    `select_branch`(*root\_atom\_index*, *directionality\_atom\_index*)¶
    :   Identify an isolated “branch” of a molecular model. Assumes the
        atoms with indices root\_atom\_index and directionality\_atom\_index are
        bound to one another and that the branch starts at root\_atom\_index one
        and “points” in the direction of directionality\_atom\_index.

        Requires the `numpy` library.

        Should be called via the wrapper function
        `select_branch()`.

        |  |  |
        | --- | --- |
        | Parameters: | - **root\_atom\_index** (*int*) – An int, the index of the first atom in the   branch (the “root”). - **directionality\_atom\_index** (*int*) – An int, the index of the second atom   in the branch, used to establish directionality |
        | Returns: | A numpy array containing the indices of the atoms of the branch. |

    `select_close_atoms_from_different_molecules`(*other\_mol*, *cutoff*, *pairwise\_comparison=True*, *terminate\_early=False*)¶
    :   Effectively detects steric clashes between self and another
        scoria.Molecule.

        Requires the `numpy` and `scipy` libraries.

        Should be called via the wrapper function
        `select_close_atoms_from_different_molecules()`.

        |  |  |
        | --- | --- |
        | Parameters: | - **other\_mol** (*scoria.Molecule*) – A scoria.Molecule object of the other   molecule. - **cutoff** (*float*) – A float, the user-defined distance cutoff in   Angstroms. - **pairwise\_comparison** (*bool*) – An optional boolean, whether or not to   perform a simple pairwise distance comparison (if True) or   to use a more sophisitcated method (if False). True by   default. - **terminate\_early** (*bool*) – An optional boolean, whether or not to stop   looking for steric clashes once one is found. False by   default. |
        | Returns: | A tuple containing two elements. The first is a numpy.array containing the indices of all nearby atoms from this scoria.Molecule object (self). The second is a numpy.array containing the indices of all nearby atoms from the other molecule. |

    `selections_of_chains`()¶
    :   Identifies the atom selections of each chain.

        Requires the `numpy` library.

        Should be called via the wrapper function `selections_of_chains()`.

        |  |  |
        | --- | --- |
        | Returns: | A dictionary. The keys of the dictionary correspond to the chainids, and the values are numpy.array objects containing the indices of the associated chain atoms. |

    `selections_of_constituent_molecules`()¶
    :   Identifies the indices of atoms belonging to separate molecules,
        assuming that the scoria.Molecule object actually contains multiple
        physically distinct molecules that are not bound to each other via
        covalent bonds.

        Requires the `numpy` library.

        Should be called via the wrapper function
        `selections_of_constituent_molecules()`.

        |  |  |
        | --- | --- |
        | Returns: | A python list of numpy.array objects containing the indices of the atoms belonging to each molecule of the composite scoria.Molecule object. |

    `selections_of_residues`()¶
    :   Identifies the atom selections of each residue.

        Requires the `numpy` library.

        Should be called via the wrapper function
        `selections_of_residues()`.

        |  |  |
        | --- | --- |
        | Returns: | A dictionary. The keys of this dictionary correspond to the unique resname-resseq-chainid residue identifiers, and the values are numpy.array objects containing the indices of the associated residue atoms. |

### Table Of Contents

- 10. The Selections class
  - 10.1. Using the Selection functions
  - 10.2. Function Definitions

#### Previous topic

9. The Quaternion object

### This Page

- Show Source

### Quick search

### Navigation

- index
- modules |
- previous |
- scoria 2.0 documentation »

© Copyright 2016, Jacob Durrant.
Created using Sphinx 1.4.6.
