# Supplementary material for: Scoria: a Python module for manipulating 3D molecular data
Source: J Cheminform. 2017 Sep 18;9:52. doi: 10.1186/s13321-017-0237-8 (PMC5603467; doi:10.1186/s13321-017-0237-8)
Supplement: Supplementary file 2 — Additional file 2. An archived version of Scoria, without MDAnalysis support. [file 13321_2017_237_MOESM2_ESM.zip › scoria-1.0.0/docs/docs/html/AtomsAndBonds.html]

2. The AtomsAndBonds Class — scoria 2.0 documentation


### Navigation

- index
- modules |
- next |
- previous |
- scoria 2.0 documentation »

# 2. The AtomsAndBonds Class¶

## 2.1. Rationale of the AtomsAndBonds functions¶

The functions within the AtomsAndBonds framework all involve the
addition, deletion, and manipulation of individual atoms and their
bonds within the molecular framework.

### 2.1.1. Other Classes¶

To make modifications to the coordinate set of all atoms, use the
functions within the `Manipulation` class. To look
at groups of atoms with similar properties, use the functions within
the `Selections` class.

## 2.2. Function Definitions¶

*class* `scoria.AtomsAndBonds.``AtomsAndBonds`(*parent\_molecule\_object*)¶
:   A class for adding and deleting atoms and bonds. Subclass to the
    `scoria.Molecule` class.

    `add_atom`(*record\_name='ATOM'*, *serial=1*, *name='X'*, *resname='XXX'*, *chainid='X'*, *resseq=1*, *occupancy=0.0*, *tempfactor=0.0*, *charge=''*, *element='X'*, *coordinates=array([ 0.*, *0.*, *0.])*, *autoindex=True*)¶
    :   Adds an atom.

        Should be called via the wrapper function `add_atom()`.

        |  |  |
        | --- | --- |
        | Parameters: | - **record\_name** (*str*) – An optional string, the record name of the atom.   “ATOM” is the default. - **serial** (*int*) – An optional int, the serial field of the atom. 1 is   the default. - **name** (*str*) – An optional string, the name of the atom. ‘X’ is the   default. - **resname** (*str*) – An optional string, the resname of the atom. ‘XXX’   is the default. - **chainid** (*str*) – An optional string, chainid of the atom. ‘X’ is the   default. - **resseq** (*int*) – An optional int, the resseq field of the atom. 1 is   the default. - **occupancy** (*float*) – An optional float, the occupancy of the atom. 0.0   is the default. - **tempfactor** (*float*) – An optional float, the tempfactor of the atom.   0.0 is the default. - **charge** (*str*) – An optional string, the charge of the atom. ‘’ is the   default. - **element** (*str*) – An optional string, the element of the atom. ‘X’ is   the default. - **coordinates** (*numpy.array*) – An optional numpy.array, the (x, y, z)   coordinates of the atom. numpy.array([0.0, 0.0, 0.0]) is   the default. |

    `add_bond`(*index1*, *index2*, *order=1*)¶
    :   Adds a bond.

        Should be called via the wrapper function `add_bond()`.

        |  |  |
        | --- | --- |
        | Parameters: | - **index1** (*int*) – An int, the index of the first atom of the bonded   pair. - **index2** (*int*) – An int, the index of the second atom of the bonded   pair. - **order** (*int*) – An optional int, the order of the bond. 1 by default. |

    `create_bonds_by_distance`(*remove\_old\_bond\_data=True*, *delete\_excessive\_bonds=True*)¶
    :   Determines which atoms are bound to each other based on their
        proximity.

        Requires the `numpy` and `scipy` libraries.

        Should be called via the wrapper function
        `create_bonds_by_distance()`.

        |  |  |
        | --- | --- |
        | Parameters: | - **remove\_old\_bond\_data** (*bool*) – An optional boolean, whether or not to   discard old bond data before adding in bonds determined by   distance. True by default. - **delete\_excessive\_bonds** (*bool*) – An optional boolean, whether or not   to check for and delete excessive bonds. True by default. |

    `delete_atom`(*index*)¶
    :   Deletes an atom.

        Should be called via the wrapper function `delete_atom()`.

        |  |  |
        | --- | --- |
        | Parameters: | **index** (*int*) – An int, the index of the atom to delete. |

    `delete_bond`(*index1*, *index2*)¶
    :   Deletes a bond.

        Should be called via the wrapper function `delete_bond()`.

        |  |  |
        | --- | --- |
        | Parameters: | - **index1** (*int*) – An int, the index of the first atom of the bonded   pair. - **index2** (*int*) – An int, the index of the second atom of the bonded   pair. |

    `get_index_of_first_bond_partner_of_element`(*atom\_index*, *the\_element*)¶
    :   For a given atom of interest, returns the index of the first
        neighbor of a specified element.

        Requires the `numpy` and `scipy.spatial` libraries.

        Should be called via the wrapper function
        `get_index_of_first_bond_partner_of_element()`.

        |  |  |
        | --- | --- |
        | Parameters: | - **atom\_index** (*int*) – An int, the index of the atom of interest. - **the\_element** (*str*) – A string specifying the desired element of the   neighbor. |
        | Returns: | An int, the index of the first neighbor atom of the specified element. If no such neighbor exists, returns -1. |
        | Return type: | *int* |

    `get_number_of_bond_partners_of_element`(*atom\_index*, *the\_element*)¶
    :   Counts the number of atoms of a given element bonded to a specified
        atom of interest.

        Requires the `numpy` library.

        Should be called via the wrapper function
        `get_number_of_bond_partners_of_element()`.

        |  |  |
        | --- | --- |
        | Parameters: | - **atom\_index** (*int*) – An int, the index of the atom of interest. - **the\_element** (*str*) – A string describing the element of the neighbors   to be counted. |
        | Returns: | An int, the number of neighboring atoms of the specified element. |
        | Return type: | *int* |

### Table Of Contents

- 2. The AtomsAndBonds Class
  - 2.1. Rationale of the AtomsAndBonds functions
    - 2.1.1. Other Classes
  - 2.2. Function Definitions

#### Previous topic

1. The Molecule Class

#### Next topic

3. scoria.FileIO module

### This Page

- Show Source

### Quick search

### Navigation

- index
- modules |
- next |
- previous |
- scoria 2.0 documentation »

© Copyright 2016, Jacob Durrant.
Created using Sphinx 1.4.6.
