# Supplementary material for: Scoria: a Python module for manipulating 3D molecular data
Source: J Cheminform. 2017 Sep 18;9:52. doi: 10.1186/s13321-017-0237-8 (PMC5603467; doi:10.1186/s13321-017-0237-8)
Supplement: Supplementary file 2 — Additional file 2. An archived version of Scoria, without MDAnalysis support. [file 13321_2017_237_MOESM2_ESM.zip › scoria-1.0.0/docs/docs/html/md_analysis_unit_tests.html]

md\_analysis\_unit\_tests module — scoria 2.0 documentation


### Navigation

- index
- modules |
- scoria 2.0 documentation »

# md\_analysis\_unit\_tests module¶

### This Page

- Show Source

### Quick search

### Navigation

- index
- modules |
- scoria 2.0 documentation »

© Copyright 2016, Jacob Durrant.
Created using Sphinx 1.4.6.
