# Supplementary material for: Scoria: a Python module for manipulating 3D molecular data
Source: J Cheminform. 2017 Sep 18;9:52. doi: 10.1186/s13321-017-0237-8 (PMC5603467; doi:10.1186/s13321-017-0237-8)
Supplement: Supplementary file 2 — Additional file 2. An archived version of Scoria, without MDAnalysis support. [file 13321_2017_237_MOESM2_ESM.zip › scoria-1.0.0/docs/docs/html/py-modindex.html]

Python Module Index — scoria 2.0 documentation


### Navigation

- index
- modules |
- scoria 2.0 documentation »

# Python Module Index

**p**

|  |  |  |
| --- | --- | --- |
|  |  |  |
|  | **p** |  |
|  | `scoria` |  |
|  | `scoria.AtomsAndBonds` |  |
|  | `scoria.FileIO` |  |
|  | `scoria.Geometry` |  |
|  | `scoria.Information` |  |
|  | `scoria.Manipulation` |  |
|  | `scoria.Molecule` |  |
|  | `scoria.OtherMolecules` |  |
|  | `scoria.Quaternion` |  |
|  | `scoria.Selections` |  |

### Quick search

### Navigation

- index
- modules |
- scoria 2.0 documentation »

© Copyright 2016, Jacob Durrant.
Created using Sphinx 1.4.6.
