# Supplementary material for: Scoria: a Python module for manipulating 3D molecular data
Source: J Cheminform. 2017 Sep 18;9:52. doi: 10.1186/s13321-017-0237-8 (PMC5603467; doi:10.1186/s13321-017-0237-8)
Supplement: Supplementary file 2 — Additional file 2. An archived version of Scoria, without MDAnalysis support. [file 13321_2017_237_MOESM2_ESM.zip › scoria-1.0.0/docs/docs/html/REST_tutorial.html]

10. reST Documentation Guide — scoria 2.0 documentation


### Navigation

- index
- modules |
- previous |
- scoria 2.0 documentation »

# 10. reST Documentation Guide¶

## 10.1. Intro¶

This is a quick guide to get you up to speed for documenting our
python projects. We use a specific type of documenting style called
‘reST’ or reStructuredText, which allows us to quickly autopopulate
html pages from our in-code documentation. It’s light enough to put
directly into our code as comments, but has enough heft that we can
create polished web pages. (This page was also created in reST, and
can be viewed with ‘Show Source’ on the sidebar.)

These documetation stubs are only read from a string at the very
beginning of a function or class definition. Further, notes or
comments from later in the code are ignored, so any important
information should be included in the documentation string, and any
code-only relevant comments are left out of the ‘official’ documents.

The documentation style, like python itself, is whitespace and
punctuation sensitive. (It’s a markup language!) Often strangness in
the formatting can be attributed to missing spaces, unaligned
paragraphs, or a comma in place of a period. Further reading can be
done with the Sphinx Quick Intro and the reStructuredText User
Guide.

## 10.2. Basics¶

We will want every class and function to have a string documenting
what it is and how it works. A base template to work off of will look
like this:

```
def foo(bar, var):
   """
   This is a fuction that foo-ifies a variable, with regards to bar.

   Please note that this is only functioning with version foobar 4.2
   and that earlier versions are not supported.

   :param int bar: This represents the level of bar we want to foo.

   :param str var: This is the string that we will be foo-ifying.

   :returns: foo-ified var with regards to bar.
   :rtype: :class:`str`

   ::

      >>> bar = 4
      >>> var = "Hello World!"
      >>> print foo(bar, var)
      Hello World!Hello World!Hello World!Hello World!
   """

   foovar = foobar.fooify(var * bar)
   return foovar
```

The script will generate a stub like so.

`foo`(*bar*, *var*)¶
:   This is a fuction that foo-ifies a variable, with regards to bar.

    Please note that this is only functioning with version foobar 4.2
    and that earlier versions are not supported.

    |  |  |
    | --- | --- |
    | Parameters: | - **bar** (*int*) – This represents the level of bar we want to foo. - **var** (*str*) – This is the string that we will be foo-ifying. |
    | Returns: | foo-ified var with regards to bar. |
    | Return type: | `str` |

    ```
    >>> bar = 4
    >>> var = "Hello World!"
    >>> print foo(bar, var)
    Hello World!Hello World!Hello World!Hello World!
    ```

There are a few things to include with a good documentation string.

- A short description of the function in plain english, with any
  caveats or non-standard behaviours.
- A note of dependancies or relationships between this code and other
  functions or librarie, if any.
- A list of any parameters that the function takes. These should be
  prefaced with `:param type name:` which will be autopopulated when
  the documentation is generated.
- A description of the return value of the function. This should be
  prefaced with `:returns:`. The variable type that is returned
  should be prefaced with `:rtype:` and a link to the object that it
  should return.
- Finally an example is sometimes a good way to end a documentation
  section. A literal section of code is created with the `::` as
  either a stand alone will allow you to enter a quick example of the
  function.

These segments are almost all optional. If a function doesn’t take
parameters, or give a return value, then the documentation shouldn’t
cover it. Likewise, if there are no dependancies or necessary
examples, then those sections should be omitted.

An example of an alternate layout would be a setter function as
follows:

```
def set_namespace(self, namespace):
   """
   Sets the namespace for the function.

   :param str namespace: A string containing the new namespace.
   """

   self.__namespace = namespace
```

## 10.3. Advanced¶

### 10.3.1. Linking¶

Inter- and intra-document links can be made with the :any:,
:class:, or :func: preface. This allows us to connect related
segments of the documentation together. Connections can be made to
classes, `scoria.Molecule`, or to specific functions,
`load_pdb_into()`. They can also be used
to link to definitions outside the documentation base, such as linking
to the `scipy.spatial` module. Note: to link to outside
documentation libraries, the url of the library must be added to the
sphinx configuration file. Additionally, adding a tilde before a long
function definition will truncate to show just the last section.

| Type | Preface | Example |
| --- | --- | --- |
| Unknown | :any: | `int` |
| Module | :mod: | `numpy` |
| Class | :class: | `scoria.Information` |
| Function | :func: | `scoria.Information.Information.get_trajectory()` |
| Function | :func: + ~ | `get_trajectory()` |

### Table Of Contents

- 10. reST Documentation Guide
  - 10.1. Intro
  - 10.2. Basics
  - 10.3. Advanced
    - 10.3.1. Linking

#### Previous topic

9. scoria.Selections module

### This Page

- Show Source

### Quick search

### Navigation

- index
- modules |
- previous |
- scoria 2.0 documentation »

© Copyright 2016, Jacob Durrant.
Created using Sphinx 1.4.6.
