# Supplementary figures and images for: Scoria: a Python module for manipulating 3D molecular data
Source: J Cheminform. 2017 Sep 18;9:52. doi: 10.1186/s13321-017-0237-8 (PMC5603467; doi:10.1186/s13321-017-0237-8)

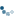

Supplement: Supplementary file 2 — Additional file 2. An archived version of Scoria, without MDAnalysis support. [file 13321_2017_237_MOESM2_ESM.zip › scoria-1.0.0/docs/build/html/_static/ajax-loader.gif]

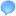

Supplement: Supplementary file 2 — Additional file 2. An archived version of Scoria, without MDAnalysis support. [file 13321_2017_237_MOESM2_ESM.zip › scoria-1.0.0/docs/build/html/_static/comment-bright.png]

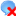

Supplement: Supplementary file 2 — Additional file 2. An archived version of Scoria, without MDAnalysis support. [file 13321_2017_237_MOESM2_ESM.zip › scoria-1.0.0/docs/build/html/_static/comment-close.png]

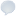

Supplement: Supplementary file 2 — Additional file 2. An archived version of Scoria, without MDAnalysis support. [file 13321_2017_237_MOESM2_ESM.zip › scoria-1.0.0/docs/build/html/_static/comment.png]

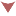

Supplement: Supplementary file 2 — Additional file 2. An archived version of Scoria, without MDAnalysis support. [file 13321_2017_237_MOESM2_ESM.zip › scoria-1.0.0/docs/build/html/_static/down-pressed.png]

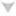

Supplement: Supplementary file 2 — Additional file 2. An archived version of Scoria, without MDAnalysis support. [file 13321_2017_237_MOESM2_ESM.zip › scoria-1.0.0/docs/build/html/_static/down.png]

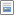

Supplement: Supplementary file 2 — Additional file 2. An archived version of Scoria, without MDAnalysis support. [file 13321_2017_237_MOESM2_ESM.zip › scoria-1.0.0/docs/build/html/_static/file.png]

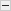

Supplement: Supplementary file 2 — Additional file 2. An archived version of Scoria, without MDAnalysis support. [file 13321_2017_237_MOESM2_ESM.zip › scoria-1.0.0/docs/build/html/_static/minus.png]

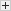

Supplement: Supplementary file 2 — Additional file 2. An archived version of Scoria, without MDAnalysis support. [file 13321_2017_237_MOESM2_ESM.zip › scoria-1.0.0/docs/build/html/_static/plus.png]

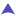

Supplement: Supplementary file 2 — Additional file 2. An archived version of Scoria, without MDAnalysis support. [file 13321_2017_237_MOESM2_ESM.zip › scoria-1.0.0/docs/build/html/_static/up-pressed.png]

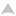

Supplement: Supplementary file 2 — Additional file 2. An archived version of Scoria, without MDAnalysis support. [file 13321_2017_237_MOESM2_ESM.zip › scoria-1.0.0/docs/build/html/_static/up.png]
