# Supplementary material for: Scoria: a Python module for manipulating 3D molecular data
Source: J Cheminform. 2017 Sep 18;9:52. doi: 10.1186/s13321-017-0237-8 (PMC5603467; doi:10.1186/s13321-017-0237-8)
Supplement: Supplementary file 3 — Additional file 3. An archived version of Scoria, derived from the main Scoria branch, that includes MDAnalysis support. [file 13321_2017_237_MOESM3_ESM.zip › scoria-1.0.0_mda/docs/build/html/Manipulation.html]

7. The Manipulation class — scoria 2.0 documentation


### Navigation

- index
- modules |
- next |
- previous |
- scoria 2.0 documentation »

# 7. The Manipulation class¶

## 7.1. Rationale of the Manipulation functions¶

The functions in the Manipulation class modify the full coordinate set of the
Molecule. The coordinate set can be shifted and rotated based on several
criteria.

## 7.2. Function Definitions¶

*class* `scoria_mda.Manipulation.``Manipulation`(*parent\_molecule\_object*)¶
:   A class for translating and rotating the atomic coordinates of a
    scoria\_mda.Molecule object

    `coordinate_undo`()¶
    :   Resets the coordinates of all atoms to those saved using the
        set\_coordinate\_undo\_point function.

    `rotate_molecule_around_a_line_between_atoms`(*line\_point1\_index*, *line\_point2\_index*, *rotate*)¶
    :   Rotate the molecular model about a line segment. The end points of
        the line segment are atoms of specified indices.

        Wrapper function for
        `rotate_molecule_around_a_line_between_atoms()`

        |  |  |
        | --- | --- |
        | Parameters: | - **line\_point1\_index** (*int*) – An int, the index of the first atom at one   end of the line segment. - **line\_point2\_index** (*int*) – An int, the index of the second atom at   the other end of the line segment. - **rotate** (*float*) – A float, the angle of rotation, in radians. |

    `rotate_molecule_around_a_line_between_points`(*line\_point1*, *line\_point2*, *rotate*)¶
    :   Rotate the molecular model about a line segment. The end points of
        the line segment are explicitly specified coordinates.

        Wrapper function for
        `rotate_molecule_around_a_line_between_points()`

        |  |  |
        | --- | --- |
        | Parameters: | - **line\_point1** (*numpy.array*) – A numpy.array (x, y, z) corresponding to one end   of the line segment. - **line\_point2** (*numpy.array*) – A numpy.array (x, y, z) corresponding to the   other end of the line segment. - **rotate** (*float*) – A float, the angle of rotation, in radians. |

    `rotate_molecule_around_pivot_atom`(*pivot\_index*, *thetax*, *thetay*, *thetaz*)¶
    :   Rotate the molecular model around a specified atom.

        Requires the `numpy` library.

        Wrapper function for `rotate_molecule_around_pivot_atom()`

        |  |  |
        | --- | --- |
        | Parameters: | - **pivot\_index** (*int*) – An int, the index of the atom about which the   molecular model will be rotated. - **thetax** (*float*) – A float, the angle to rotate relative to the x axis,   in radians. - **thetay** (*float*) – A float, the angle to rotate relative to the y axis,   in radians. - **thetaz** (*float*) – A float, the angle to rotate relative to the z axis,   in radians. |

    `rotate_molecule_around_pivot_point`(*pivot*, *thetax*, *thetay*, *thetaz*)¶
    :   Rotate the molecular model around a specified atom.

        Requires the `numpy` library.

        Wrapper function for `rotate_molecule_around_pivot_point()`

        |  |  |
        | --- | --- |
        | Parameters: | - **pivot** (*numpy.array*) – A numpy.array, the (x, y, z) coordinate about which   the molecular model will be rotated. - **thetax** (*float*) – A float, the angle to rotate relative to the x axis,   in radians. - **thetay** (*float*) – A float, the angle to rotate relative to the y axis,   in radians. - **thetaz** (*float*) – A float, the angle to rotate relative to the z axis,   in radians. |

    `set_atom_location`(*atom\_index*, *new\_location*)¶
    :   Translates the entire molecular model (without rotating) so that the
        atom with the specified index is located at the specified coordinate.

        Wrapper function for `set_atom_location()`

        |  |  |
        | --- | --- |
        | Parameters: | - **atom\_index** (*int*) – An int, the index of the target atom. - **new\_location** (*numpy.array*) – A numpy.array specifying the new (x, y, z)   coordinate of the specified atom. |
        | Returns: | A numpy.array specifying the (delta\_x, delta\_y, delta\_z) vector by which the pmolecule.Molecule was translated. |

    `set_coordinate_undo_point`()¶
    :   Sets (“saves”) the undo point of the atom coordinates. Any
        subsequent manipulations of atomic coordinates can be “undone” by
        reseting to this configuration via the coordinate\_undo function.

    `translate_molecule`(*delta*)¶
    :   Translate all the atoms of the molecular model by a specified
        vector.

        Wrapper function for `translate_molecule()`

        |  |  |
        | --- | --- |
        | Parameters: | **delta** (*numpy.array*) – A numpy.array (delta\_x, delta\_y, delta\_z) specifying the amount to move each atom along the x, y, and z coordinates. |

### Table Of Contents

- 7. The Manipulation class
  - 7.1. Rationale of the Manipulation functions
  - 7.2. Function Definitions

#### Previous topic

6. The Information class

#### Next topic

8. The OtherMolecules class

### This Page

- Show Source

### Quick search

### Navigation

- index
- modules |
- next |
- previous |
- scoria 2.0 documentation »

© Copyright 2016, Jacob Durrant.
Created using Sphinx 1.4.6.
