# Supplementary material for: Scoria: a Python module for manipulating 3D molecular data
Source: J Cheminform. 2017 Sep 18;9:52. doi: 10.1186/s13321-017-0237-8 (PMC5603467; doi:10.1186/s13321-017-0237-8)
Supplement: Supplementary file 3 — Additional file 3. An archived version of Scoria, derived from the main Scoria branch, that includes MDAnalysis support. [file 13321_2017_237_MOESM3_ESM.zip › scoria-1.0.0_mda/docs/build/html/Quaternion.html]

9. The Quaternion object — scoria 2.0 documentation


### Navigation

- index
- modules |
- next |
- previous |
- scoria 2.0 documentation »

# 9. The Quaternion object¶

## 9.1. Using Quternion object¶

Test. Needs additional Documentation.

## 9.2. Function Definitions¶

*class* `scoria_mda.Quaternion.``Quaternion`(*s*, *x*, *y*, *z*)¶
:   A class supporting quaternion arithmetic

    `add`(*q2*)¶
    :   Adds two quaternions.

        |  |  |
        | --- | --- |
        | Parameters: | **q2** (*scoria\_mda.Quaternion*) – A quaternion, to be added to self |
        | Returns: | A Quaternion, with the values corresponding to self + q2 |

    `copy`()¶
    :   Returns a copy of self

    `invert`()¶
    :   Takes the inverse of the quaternion for “division.”

        |  |  |
        | --- | --- |
        | Returns: | A Quaternion, with the values corresponding to self^-1 |

    `load_from_mat`(*m*)¶
    :   Converts a rotation matrix that is pure orthogonal (det(matrix)=1)
        into a Quaternion. Adapted from http://www.euclideanspace.com/maths/
        geometry/rotations/conversions/matrixToQuaternion/index.htm

        |  |  |
        | --- | --- |
        | Parameters: | **m** (*numpy.array*) – A 2D numpy.array representing a pure orthogonal matrix |

    `minus`(*q2*)¶
    :   Multiplies two quaternions.

        |  |  |
        | --- | --- |
        | Parameters: | **q2** (*scoria\_mda.Quaternion*) – A quaternion, to be subtracted from self |

        Returns:
        :   A Quaternion, with the values corresponding to self - q2

    `multiply`(*q2*)¶
    :   Multiplies two quaternions.

        |  |  |
        | --- | --- |
        | Parameters: | **q2** (*scoria\_mda.Quaternion*) – A quaternion, to be multiplied with self |
        | Returns: | A Quaternion, with the values corresponding to self \* q2 |

    `normalize`()¶
    :   Normalizes the quaternion.

        |  |  |
        | --- | --- |
        | Returns: | A normalized Quaternion |

    `rep_as_44_matrix`()¶
    :   Creates a 4x4 matrix representation of the Quaternion.

        |  |  |
        | --- | --- |
        | Returns: | A 4x4 numpy array |

    `scale`(*scalar*)¶
    :   Scales a quaternion.

        |  |  |
        | --- | --- |
        | Parameters: | **scalar** (*???*) – the value to scale the quaternion by |
        | Returns: | A Quaternion, with the values corresponding to self \* scalar |

    `to_matrix`()¶
    :   Converts to a normalized 3x3 matrix.

        |  |  |
        | --- | --- |
        | Returns: | A 3x3 numpy.array, corresponding to the quaternion |

### Table Of Contents

- 9. The Quaternion object
  - 9.1. Using Quternion object
  - 9.2. Function Definitions

#### Previous topic

8. The OtherMolecules class

#### Next topic

10. The Selections class

### This Page

- Show Source

### Quick search

### Navigation

- index
- modules |
- next |
- previous |
- scoria 2.0 documentation »

© Copyright 2016, Jacob Durrant.
Created using Sphinx 1.4.6.
