# Supplementary material for: Scoria: a Python module for manipulating 3D molecular data
Source: J Cheminform. 2017 Sep 18;9:52. doi: 10.1186/s13321-017-0237-8 (PMC5603467; doi:10.1186/s13321-017-0237-8)
Supplement: Supplementary file 3 — Additional file 3. An archived version of Scoria, derived from the main Scoria branch, that includes MDAnalysis support. [file 13321_2017_237_MOESM3_ESM.zip › scoria-1.0.0_mda/docs/build/html/Selections.html]

10. The Selections class — scoria 2.0 documentation


### Navigation

- index
- modules |
- previous |
- scoria 2.0 documentation »

# 10. The Selections class¶

## 10.1. Using the Selection functions¶

Functions within the Selection module search the molecule given specific
criteria and return a list of indexes that match that criteria.

## 10.2. Function Definitions¶

*class* `scoria_mda.Selections.``Selections`(*parent\_molecule\_object*)¶
:   A class for selecting atoms. Subclass to the
    `scoria_mda.Molecule` class.

    `get_molecule_from_selection`(*selection*, *serial\_reindex=True*, *resseq\_reindex=False*)¶
    :   Creates a scoria\_mda.Molecule from a user-defined atom selection.

        Should be called via the wrapper function `get_molecule_from_selection()`.

        |  |  |
        | --- | --- |
        | Parameters: | - **selection** (*numpy.array*) – A numpy.array containing the indices of the atoms   in the user-defined selection. - **serial\_reindex** (*bool*) – An optional boolean, whether or not to   reindex the atom serial fields. Default is True. - **resseq\_reindex** (*bool*) – An optional boolean, whether or not to   reindex the atom resseq fields. Default is False. |
        | Returns: | A scoria\_mda.Molecule object containing the atoms of the user-defined selection. |

    `invert_selection`(*selection*)¶
    :   Inverts a user-defined selection (i.e., identifies all atoms that
        are not in the seleciton).

        Should be called via the wrapper function
        `invert_selection()`.

        |  |  |
        | --- | --- |
        | Parameters: | **selection** (*numpy.array*) – A numpy.array containing the indices of the user-defined selection. |
        | Returns: | A numpy.array containing the indices of all atoms that are not in the user-defined seleciton. |

    `select_all`()¶
    :   Selects all the atoms in a scoria\_mda.Molecule object.

        Should be called via the wrapper function `select_all()`.

        |  |  |
        | --- | --- |
        | Returns: | A numpy.array containing the indices of all atoms in the scoria\_mda.Molecule object. |

    `select_all_atoms_bound_to_selection`(*selection*)¶
    :   Selects all the atoms that are bound to a user-specified selection.

        Requires the `numpy` library.

        Should be called via the wrapper function
        `select_all_atoms_bound_to_selection()`.

        |  |  |
        | --- | --- |
        | Parameters: | **selection** (*numpy.array*) – A numpy.array containing the indices of the user-specified selection. |
        | Returns: | A numpy.array containing the indices of the atoms that are bound to the user-specified selection. Note that this new selection does not necessarily include the indices of the original user-specified selection. |

    `select_atoms`(*selection\_criteria*)¶
    :   Select a set of atoms based on user-specified criteria.

        Should be called via the wrapper function `select_atoms()`.

        |  |  |
        | --- | --- |
        | Parameters: | **selection\_criteria** (*dict*) – A dictionary, where the keys correspond to keys in the self.\_\_parent\_Information.Information.get\_atom\_information() structured numpy array, and the values are lists of acceptable matches. The selection is a logical “AND” between dictionary entries, but “OR” within the value lists themselves. For example: {‘atom’:[‘CA’, ‘O’], ‘chain’:’A’, ‘resname\_padded’:’PRO’} would select all atoms with the names CA or O that are located in the PRO residues of chain A. |
        | Returns: | A numpy.array containing the indices of the atoms of the selection. |

    `select_atoms_from_same_molecule`(*selection*)¶
    :   Selects all the atoms that belong to the same molecule as a
        user-defined selection, assuming that the scoria\_mda.Molecule object
        actually contains multiple physically distinct molecules that are not
        bound to each other via covalent bonds.

        Requires the `numpy` library.

        Should be called via the wrapper function
        `select_atoms_from_same_molecule()`.

        |  |  |
        | --- | --- |
        | Parameters: | **selection** (*numpy.array*) – A numpy.array containing the indices of the user-defined selection. |
        | Returns: | A numpy.array containing the indices of the atoms belonging to the same molecules as the atoms of the user-defined selection. |

    `select_atoms_in_bounding_box`(*bounding\_box*)¶
    :   Selects all the atoms that are within a bounding box.

        Requires the `numpy` library.

        Should be called via the wrapper function
        `select_atoms_in_bounding_box()`.

        |  |  |
        | --- | --- |
        | Parameters: | **bounding\_box** (*numpy.array*) – A 2x3 numpy.array containing the minimum and maximum points of the bounding box. Example: numpy.array( [[min\_x, min\_y, min\_z], [max\_x, max\_y, max\_z]] ). |
        | Returns: | A numpy.array containing the indices of the atoms that are within the bounding box. |

    `select_atoms_in_same_residue`(*selection*)¶
    :   Selects all atoms that are in the same residue as any of the atoms
        of a user-defined seleciton. Residues are considered unique if they
        have a unique combination of resname, resseq, and chainid fields.

        Should be called via the wrapper function
        `select_atoms_in_same_residue()`.

        |  |  |
        | --- | --- |
        | Parameters: | **selection** (*numpy.array*) – A numpy.array containing the indices of the user-defined selection. |
        | Returns: | A numpy.array containing the indices of all atoms in the same residue as any of the atoms of the user-defined selection. |

    `select_atoms_near_other_selection`(*selection*, *cutoff*)¶
    :   Selects all atoms that are near the atoms of a user-defined
        selection.

        Requires the `numpy` and `scipy` libraries.

        Should be called via the wrapper function
        `select_atoms_near_other_selection()`.

        |  |  |
        | --- | --- |
        | Parameters: | - **selection** (*numpy.array*) – A numpy.array containing the indices of the   user-defined selection. - **cutoff** (*float*) – A float, the distance cutoff (in Angstroms). |
        | Returns: | A numpy.array containing the indices of all atoms near the user-defined selection, not including the atoms of the user-defined selection themselves. |

    `select_branch`(*root\_atom\_index*, *directionality\_atom\_index*)¶
    :   Identify an isolated “branch” of a molecular model. Assumes the
        atoms with indices root\_atom\_index and directionality\_atom\_index are
        bound to one another and that the branch starts at root\_atom\_index one
        and “points” in the direction of directionality\_atom\_index.

        Requires the `numpy` library.

        Should be called via the wrapper function
        `select_branch()`.

        |  |  |
        | --- | --- |
        | Parameters: | - **root\_atom\_index** (*int*) – An int, the index of the first atom in the   branch (the “root”). - **directionality\_atom\_index** (*int*) – An int, the index of the second atom   in the branch, used to establish directionality |
        | Returns: | A numpy array containing the indices of the atoms of the branch. |

    `select_close_atoms_from_different_molecules`(*other\_mol*, *cutoff*, *pairwise\_comparison=True*, *terminate\_early=False*)¶
    :   Effectively detects steric clashes between self and another
        scoria\_mda.Molecule.

        Requires the `numpy` and `scipy` libraries.

        Should be called via the wrapper function
        `select_close_atoms_from_different_molecules()`.

        |  |  |
        | --- | --- |
        | Parameters: | - **other\_mol** (*scoria\_mda.Molecule*) – A scoria\_mda.Molecule object of the other   molecule. - **cutoff** (*float*) – A float, the user-defined distance cutoff in   Angstroms. - **pairwise\_comparison** (*bool*) – An optional boolean, whether or not to   perform a simple pairwise distance comparison (if True) or   to use a more sophisitcated method (if False). True by   default. - **terminate\_early** (*bool*) – An optional boolean, whether or not to stop   looking for steric clashes once one is found. False by   default. |
        | Returns: | A tuple containing two elements. The first is a numpy.array containing the indices of all nearby atoms from this scoria\_mda.Molecule object (self). The second is a numpy.array containing the indices of all nearby atoms from the other molecule. |

    `selections_of_chains`()¶
    :   Identifies the atom selections of each chain.

        Requires the `numpy` library.

        Should be called via the wrapper function `selections_of_chains()`.

        |  |  |
        | --- | --- |
        | Returns: | A dictionary. The keys of the dictionary correspond to the chainids, and the values are numpy.array objects containing the indices of the associated chain atoms. |

    `selections_of_constituent_molecules`()¶
    :   Identifies the indices of atoms belonging to separate molecules,
        assuming that the scoria\_mda.Molecule object actually contains multiple
        physically distinct molecules that are not bound to each other via
        covalent bonds.

        Requires the `numpy` library.

        Should be called via the wrapper function
        `selections_of_constituent_molecules()`.

        |  |  |
        | --- | --- |
        | Returns: | A python list of numpy.array objects containing the indices of the atoms belonging to each molecule of the composite scoria\_mda.Molecule object. |

    `selections_of_residues`()¶
    :   Identifies the atom selections of each residue.

        Requires the `numpy` library.

        Should be called via the wrapper function
        `selections_of_residues()`.

        |  |  |
        | --- | --- |
        | Returns: | A dictionary. The keys of this dictionary correspond to the unique resname-resseq-chainid residue identifiers, and the values are numpy.array objects containing the indices of the associated residue atoms. |

### Table Of Contents

- 10. The Selections class
  - 10.1. Using the Selection functions
  - 10.2. Function Definitions

#### Previous topic

9. The Quaternion object

### This Page

- Show Source

### Quick search

### Navigation

- index
- modules |
- previous |
- scoria 2.0 documentation »

© Copyright 2016, Jacob Durrant.
Created using Sphinx 1.4.6.
