# Supplementary material for: Scoria: a Python module for manipulating 3D molecular data
Source: J Cheminform. 2017 Sep 18;9:52. doi: 10.1186/s13321-017-0237-8 (PMC5603467; doi:10.1186/s13321-017-0237-8)
Supplement: Supplementary file 3 — Additional file 3. An archived version of Scoria, derived from the main Scoria branch, that includes MDAnalysis support. [file 13321_2017_237_MOESM3_ESM.zip › scoria-1.0.0_mda/docs/docs/html/genindex.html]

Index — scoria 2.0 documentation


### Navigation

- index
- modules |
- scoria 2.0 documentation »

# Index

**A**
| **B**
| **C**
| **D**
| **F**
| **G**
| **I**
| **L**
| **M**
| **N**
| **O**
| **P**
| **Q**
| **R**
| **S**
| **T**

## A

|  |  |
| --- | --- |
| add() (scoria\_mda.Quaternion.Quaternion method)  add\_atom() (scoria\_mda.AtomsAndBonds.AtomsAndBonds method)  (scoria\_mda.Molecule.Molecule method)  add\_bond() (scoria\_mda.AtomsAndBonds.AtomsAndBonds method)  (scoria\_mda.Molecule.Molecule method) | assign\_elements\_from\_atom\_names() (scoria\_mda.Information.Information method)  (scoria\_mda.Molecule.Molecule method)  assign\_masses() (scoria\_mda.Information.Information method)  (scoria\_mda.Molecule.Molecule method)  AtomsAndBonds (class in scoria\_mda.AtomsAndBonds) |

## B

|  |  |
| --- | --- |
| belongs\_to\_dna() (scoria\_mda.Information.Information method)  (scoria\_mda.Molecule.Molecule method)  belongs\_to\_protein() (scoria\_mda.Information.Information method)  (scoria\_mda.Molecule.Molecule method) | belongs\_to\_rna() (scoria\_mda.Information.Information method)  (scoria\_mda.Molecule.Molecule method) |

## C

|  |  |
| --- | --- |
| coordinate\_undo() (scoria\_mda.Manipulation.Manipulation method)  (scoria\_mda.Molecule.Molecule method)  copy() (scoria\_mda.Molecule.Molecule method)  (scoria\_mda.Quaternion.Quaternion method) | create\_bonds\_by\_distance() (scoria\_mda.AtomsAndBonds.AtomsAndBonds method)  (scoria\_mda.Molecule.Molecule method) |

## D

|  |  |
| --- | --- |
| define\_molecule\_chain\_residue\_spherical\_boundaries() (scoria\_mda.Information.Information method)  (scoria\_mda.Molecule.Molecule method)  delete\_atom() (scoria\_mda.AtomsAndBonds.AtomsAndBonds method)  (scoria\_mda.Molecule.Molecule method) | delete\_bond() (scoria\_mda.AtomsAndBonds.AtomsAndBonds method)  (scoria\_mda.Molecule.Molecule method)  delete\_trajectory\_frame() (scoria\_mda.Information.Information method)  (scoria\_mda.Molecule.Molecule method) |

## F

|  |  |
| --- | --- |
| FileIO (class in scoria\_mda.FileIO) | foo() |

## G

|  |  |
| --- | --- |
| Geometry (class in scoria\_mda.Geometry)  get\_angle\_between\_three\_points() (scoria\_mda.Geometry.Geometry method)  (scoria\_mda.Molecule.Molecule method)  get\_atom\_information() (scoria\_mda.Information.Information method)  (scoria\_mda.Molecule.Molecule method)  get\_bonds() (scoria\_mda.Information.Information method)  (scoria\_mda.Molecule.Molecule method)  get\_bounding\_box() (scoria\_mda.Information.Information method)  (scoria\_mda.Molecule.Molecule method)  get\_bounding\_sphere() (scoria\_mda.Information.Information method)  (scoria\_mda.Molecule.Molecule method)  get\_center\_of\_mass() (scoria\_mda.Information.Information method)  (scoria\_mda.Molecule.Molecule method)  get\_constants() (scoria\_mda.Information.Information method)  (scoria\_mda.Molecule.Molecule method)  get\_coordinates() (scoria\_mda.Information.Information method)  (scoria\_mda.Molecule.Molecule method)  get\_coordinates\_undo\_point() (scoria\_mda.Information.Information method)  (scoria\_mda.Molecule.Molecule method)  get\_default\_trajectory\_frame() (scoria\_mda.Information.Information method)  (scoria\_mda.Molecule.Molecule method)  get\_dihedral\_angle() (scoria\_mda.Geometry.Geometry method)  (scoria\_mda.Molecule.Molecule method)  get\_distance\_to\_another\_molecule() (scoria\_mda.Molecule.Molecule method)  (scoria\_mda.OtherMolecules.OtherMolecules method)  get\_filename() (scoria\_mda.Information.Information method)  (scoria\_mda.Molecule.Molecule method)  get\_geometric\_center() (scoria\_mda.Information.Information method)  (scoria\_mda.Molecule.Molecule method) | get\_hierarchy() (scoria\_mda.Information.Information method)  (scoria\_mda.Molecule.Molecule method)  get\_index\_of\_first\_bond\_partner\_of\_element() (scoria\_mda.AtomsAndBonds.AtomsAndBonds method)  (scoria\_mda.Molecule.Molecule method)  get\_molecule\_from\_selection() (scoria\_mda.Molecule.Molecule method)  (scoria\_mda.Selections.Selections method)  get\_number\_of\_bond\_partners\_of\_element() (scoria\_mda.AtomsAndBonds.AtomsAndBonds method)  (scoria\_mda.Molecule.Molecule method)  get\_other\_molecule\_aligned\_to\_this() (scoria\_mda.Molecule.Molecule method)  (scoria\_mda.OtherMolecules.OtherMolecules method)  get\_planarity\_deviation() (scoria\_mda.Geometry.Geometry method)  (scoria\_mda.Molecule.Molecule method)  get\_remarks() (pymolecule.Information.Information method)  (pymolecule.Molecule.Molecule method)  get\_rmsd\_equivalent\_atoms\_specified() (pymolecule.Molecule.Molecule method)  (pymolecule.OtherMolecules.OtherMolecules method)  get\_rmsd\_heuristic() (pymolecule.Molecule.Molecule method)  (pymolecule.OtherMolecules.OtherMolecules method)  get\_rmsd\_order\_dependent() (pymolecule.Molecule.Molecule method)  (pymolecule.OtherMolecules.OtherMolecules method)  get\_total\_mass() (pymolecule.Information.Information method)  (pymolecule.Molecule.Molecule method)  get\_total\_number\_of\_atoms() (pymolecule.Information.Information method)  (pymolecule.Molecule.Molecule method)  get\_total\_number\_of\_heavy\_atoms() (pymolecule.Information.Information method)  (pymolecule.Molecule.Molecule method)  get\_trajectory() (pymolecule.Information.Information method)  (pymolecule.Molecule.Molecule method)  get\_trajectory\_frame\_count() (pymolecule.Information.Information method)  (pymolecule.Molecule.Molecule method) |

## I

|  |  |
| --- | --- |
| Information (class in pymolecule.Information)  insert\_trajectory\_frame() (pymolecule.Information.Information method)  (pymolecule.Molecule.Molecule method)  invert() (pymolecule.Quaternion.Quaternion method) | invert\_selection() (pymolecule.Molecule.Molecule method)  (pymolecule.Selections.Selections method)  is\_planar() (pymolecule.Geometry.Geometry method)  (pymolecule.Molecule.Molecule method) |

## L

|  |  |
| --- | --- |
| load\_from\_mat() (pymolecule.Quaternion.Quaternion method)  load\_MDAnalysis\_into() (pymolecule.FileIO.FileIO method)  load\_pdb\_into() (pymolecule.FileIO.FileIO method)  (pymolecule.Molecule.Molecule method)  load\_pdb\_into\_using\_file\_object() (pymolecule.FileIO.FileIO method)  (pymolecule.Molecule.Molecule method)  load\_pdb\_trajectory\_into() (pymolecule.FileIO.FileIO method)  (pymolecule.Molecule.Molecule method)  load\_pdb\_trajectory\_into\_using\_file\_object() (pymolecule.FileIO.FileIO method)  (pymolecule.Molecule.Molecule method) | load\_pdbqt\_into() (pymolecule.FileIO.FileIO method)  (pymolecule.Molecule.Molecule method)  load\_pdbqt\_into\_using\_file\_object() (pymolecule.FileIO.FileIO method)  (pymolecule.Molecule.Molecule method)  load\_pdbqt\_trajectory\_into() (pymolecule.FileIO.FileIO method)  (pymolecule.Molecule.Molecule method)  load\_pdbqt\_trajectory\_into\_using\_file\_object() (pymolecule.FileIO.FileIO method)  (pymolecule.Molecule.Molecule method)  load\_pym\_into() (pymolecule.FileIO.FileIO method)  (pymolecule.Molecule.Molecule method)  load\_via\_MDAnalysis() (pymolecule.FileIO.FileIO method)  (pymolecule.Molecule.Molecule method) |

## M

|  |  |
| --- | --- |
| Manipulation (class in pymolecule.Manipulation)  merge\_with\_another\_molecule() (pymolecule.Molecule.Molecule method)  (pymolecule.OtherMolecules.OtherMolecules method)  minus() (pymolecule.Quaternion.Quaternion method) | Molecule (class in pymolecule.Molecule)  multiply() (pymolecule.Quaternion.Quaternion method) |

## N

|  |  |
| --- | --- |
| normalize() (pymolecule.Quaternion.Quaternion method) | numpy\_structured\_array\_remove\_field() (pymolecule.Molecule.Molecule method) |

## O

|  |
| --- |
| OtherMolecules (class in pymolecule.OtherMolecules) |

## P

|  |  |
| --- | --- |
| pymolecule.AtomsAndBonds (module)  pymolecule.FileIO (module)  pymolecule.Geometry (module)  pymolecule.Information (module)  pymolecule.Manipulation (module) | pymolecule.Molecule (module)  pymolecule.OtherMolecules (module)  pymolecule.Quaternion (module)  pymolecule.Selections (module) |

## Q

|  |
| --- |
| Quaternion (class in pymolecule.Quaternion) |

## R

|  |  |
| --- | --- |
| rep\_as\_44\_matrix() (pymolecule.Quaternion.Quaternion method)  resseq\_reindex() (pymolecule.Information.Information method)  (pymolecule.Molecule.Molecule method)  rotate\_molecule\_around\_a\_line\_between\_atoms() (pymolecule.Manipulation.Manipulation method)  (pymolecule.Molecule.Molecule method) | rotate\_molecule\_around\_a\_line\_between\_points() (pymolecule.Manipulation.Manipulation method)  (pymolecule.Molecule.Molecule method)  rotate\_molecule\_around\_pivot\_atom() (pymolecule.Manipulation.Manipulation method)  (pymolecule.Molecule.Molecule method)  rotate\_molecule\_around\_pivot\_point() (pymolecule.Manipulation.Manipulation method)  (pymolecule.Molecule.Molecule method) |

## S

|  |  |
| --- | --- |
| save\_pdb() (pymolecule.FileIO.FileIO method)  (pymolecule.Molecule.Molecule method)  save\_pym() (pymolecule.FileIO.FileIO method)  (pymolecule.Molecule.Molecule method)  scale() (pymolecule.Quaternion.Quaternion method)  select\_all() (pymolecule.Molecule.Molecule method)  (pymolecule.Selections.Selections method)  select\_all\_atoms\_bound\_to\_selection() (pymolecule.Molecule.Molecule method)  (pymolecule.Selections.Selections method)  select\_atoms() (pymolecule.Molecule.Molecule method)  (pymolecule.Selections.Selections method)  select\_atoms\_from\_same\_molecule() (pymolecule.Molecule.Molecule method)  (pymolecule.Selections.Selections method)  select\_atoms\_in\_bounding\_box() (pymolecule.Molecule.Molecule method)  (pymolecule.Selections.Selections method)  select\_atoms\_in\_same\_residue() (pymolecule.Molecule.Molecule method)  (pymolecule.Selections.Selections method)  select\_atoms\_near\_other\_selection() (pymolecule.Molecule.Molecule method)  (pymolecule.Selections.Selections method)  select\_branch() (pymolecule.Molecule.Molecule method)  (pymolecule.Selections.Selections method)  select\_close\_atoms\_from\_different\_molecules() (pymolecule.Molecule.Molecule method)  (pymolecule.Selections.Selections method)  Selections (class in pymolecule.Selections)  selections\_of\_chains() (pymolecule.Molecule.Molecule method)  (pymolecule.Selections.Selections method)  selections\_of\_constituent\_molecules() (pymolecule.Molecule.Molecule method)  (pymolecule.Selections.Selections method) | selections\_of\_residues() (pymolecule.Molecule.Molecule method)  (pymolecule.Selections.Selections method)  serial\_reindex() (pymolecule.Information.Information method)  (pymolecule.Molecule.Molecule method)  set\_atom\_information() (pymolecule.Information.Information method)  (pymolecule.Molecule.Molecule method)  set\_atom\_location() (pymolecule.Manipulation.Manipulation method)  (pymolecule.Molecule.Molecule method)  set\_bonds() (pymolecule.Information.Information method)  (pymolecule.Molecule.Molecule method)  set\_coordinate\_undo\_point() (pymolecule.Manipulation.Manipulation method)  set\_coordinates() (pymolecule.Information.Information method)  (pymolecule.Molecule.Molecule method)  set\_coordinates\_undo\_point() (pymolecule.Information.Information method)  (pymolecule.Molecule.Molecule method)  set\_default\_trajectory\_frame() (pymolecule.Information.Information method)  (pymolecule.Molecule.Molecule method)  set\_filename() (pymolecule.Information.Information method)  (pymolecule.Molecule.Molecule method)  set\_hierarchy() (pymolecule.Information.Information method)  (pymolecule.Molecule.Molecule method)  set\_remarks() (pymolecule.Information.Information method)  (pymolecule.Molecule.Molecule method)  set\_trajectory() (pymolecule.Information.Information method)  (pymolecule.Molecule.Molecule method)  steric\_clash\_with\_another\_molecule() (pymolecule.Molecule.Molecule method)  (pymolecule.OtherMolecules.OtherMolecules method) |

## T

|  |  |
| --- | --- |
| to\_matrix() (pymolecule.Quaternion.Quaternion method) | translate\_molecule() (pymolecule.Manipulation.Manipulation method)  (pymolecule.Molecule.Molecule method) |

### Quick search

### Navigation

- index
- modules |
- PyMolecule 2.0 documentation »

© Copyright 2016, Jacob Durrant.
Created using Sphinx 1.4.6.
