# Supplementary material for: Scoria: a Python module for manipulating 3D molecular data
Source: J Cheminform. 2017 Sep 18;9:52. doi: 10.1186/s13321-017-0237-8 (PMC5603467; doi:10.1186/s13321-017-0237-8)
Supplement: Supplementary file 3 — Additional file 3. An archived version of Scoria, derived from the main Scoria branch, that includes MDAnalysis support. [file 13321_2017_237_MOESM3_ESM.zip › scoria-1.0.0_mda/docs/docs/html/Geometry.html]

4. scoria\_mda.Geometry module — scoria 2.0 documentation


### Navigation

- index
- modules |
- next |
- previous |
- scoria 2.0 documentation »

# 4. scoria\_mda.Geometry module¶

*class* `scoria_mda.Geometry.``Geometry`(*parent\_molecule\_object*)¶
:   A class containing a few geometry functions. Note that numpy should be
    used for most geometry functions.

    `get_angle_between_three_points`(*pt1*, *pt2*, *pt3*)¶
    :   Computes the angle (in radians) formed by three points (numpy.array
        objects).

        Should be called via the wrapper function `get_angle_between_three_points()`

        |  |  |
        | --- | --- |
        | Parameters: | - **pt1** (*numpy.array*) – A numpy.array (x, y, z) representing the first of the   three 3D points. - **pt2** (*numpy.array*) – A numpy.array (x, y, z) representing the second of the   three 3D points. - **pt3** (*numpy.array*) – A numpy.array (x, y, z) representing the third of the   three 3D points. |
        | Returns: | A float containing the angle between the three points, in radians. |

    `get_dihedral_angle`(*pt1*, *pt2*, *pt3*, *pt4*)¶
    :   Calculates the dihedral angle formed by four points (numpy.array
        objects).

        Should be called via the wrapper function `get_dihedral_angle()`

        |  |  |
        | --- | --- |
        | Parameters: | - **pt1** (*numpy.array*) – A numpy.array (x, y, z) representing the first 3D   point. - **pt2** (*numpy.array*) – A numpy.array (x, y, z) representing the second 3D   point. - **pt3** (*numpy.array*) – A numpy.array (x, y, z) representing the third 3D   point. - **pt4** (*numpy.array*) – A numpy.array (x, y, z) representing the fourth 3D   point. |
        | Returns: | A float containing the dihedral angle between the four points, in radians. |

    `get_planarity_deviation`(*pt1*, *pt2*, *pt3*, *pt4*)¶
    :   Determines how close four points (numpy.array objects) come to lying
        in a common plane.

        Should be called via the wrapper function `get_planarity_deviation()`

        |  |  |
        | --- | --- |
        | Parameters: | - **pt1** (*numpy.array*) – A numpy.array (x, y, z) representing a 3D point. - **pt2** (*numpy.array*) – A numpy.array (x, y, z) representing a 3D point. - **pt3** (*numpy.array*) – A numpy.array (x, y, z) representing a 3D point. - **pt4** (*numpy.array*) – A numpy.array (x, y, z) representing a 3D point. |
        | Returns: | A float, the minimum distance between one point and the plane formed by the other three. |

    `is_planar`(*pt1*, *pt2*, *pt3*, *pt4*, *planarity\_cutoff=0.2*)¶
    :   Checks whether four points (numpy.array) lie in a common plane.

        Should be called via the wrapper function `is_planar()`

        |  |  |
        | --- | --- |
        | Parameters: | - **pt1** (*numpy.array*) – A numpy.array (x, y, z) representing a 3D point. - **pt2** (*numpy.array*) – A numpy.array (x, y, z) representing a 3D point. - **pt3** (*numpy.array*) – A numpy.array (x, y, z) representing a 3D point. - **pt4** (*numpy.array*) – A numpy.array (x, y, z) representing a 3D point. - **planarity\_cutoff** (*float*) – An optional float. How much the points can   deviate (in Angstroms) and still be considered planar. The   default is 0.2. |
        | Returns: | A boolean, whether the 4 points can be considered planar. |

#### Previous topic

3. scoria\_mda.FileIO module

#### Next topic

5. scoria\_mda.Information module

### This Page

- Show Source

### Quick search

### Navigation

- index
- modules |
- next |
- previous |
- scoria 2.0 documentation »

© Copyright 2016, Jacob Durrant.
Created using Sphinx 1.4.6.
