# Supplementary material for: Scoria: a Python module for manipulating 3D molecular data
Source: J Cheminform. 2017 Sep 18;9:52. doi: 10.1186/s13321-017-0237-8 (PMC5603467; doi:10.1186/s13321-017-0237-8)
Supplement: Supplementary file 3 — Additional file 3. An archived version of Scoria, derived from the main Scoria branch, that includes MDAnalysis support. [file 13321_2017_237_MOESM3_ESM.zip › scoria-1.0.0_mda/docs/docs/html/index.html]

Welcome to scoria’s documentation! — scoria 2.0 documentation


### Navigation

- index
- modules |
- next |
- scoria 2.0 documentation »

# Welcome to scoria’s documentation!¶

scoria is a lightweight molecular dynamics library

- 1. The Molecule Class
  - 1.1. Initiating and using the object
  - 1.2. Function Definitions
- 2. The AtomsAndBonds Class
  - 2.1. Rationale of the AtomsAndBonds functions
  - 2.2. Function Definitions
- 3. The FileIO Class
  - 3.1. File Types and Formats
  - 3.2. Function Definitions
- 4. scoria\_mda.Geometry module
- 5. scoria\_mda.Information module
- 6. scoria\_mda.Manipulation module
- 7. scoria\_mda.OtherMolecules module
- 8. scoria\_mda.Quaternion module
- 9. scoria\_mda.Selections module
- 10. reST Documentation Guide
  - 10.1. Intro
  - 10.2. Basics
  - 10.3. Advanced

# Indices and tables¶

- Index
- Module Index
- Search Page

### Table Of Contents

- Welcome to scoria’s documentation!
- Indices and tables

#### Next topic

1. The Molecule Class

### This Page

- Show Source

### Quick search

### Navigation

- index
- modules |
- next |
- scoria 2.0 documentation »

© Copyright 2016, Jacob Durrant.
Created using Sphinx 1.4.6.
