# Supplementary material for: Scoria: a Python module for manipulating 3D molecular data
Source: J Cheminform. 2017 Sep 18;9:52. doi: 10.1186/s13321-017-0237-8 (PMC5603467; doi:10.1186/s13321-017-0237-8)
Supplement: Supplementary file 3 — Additional file 3. An archived version of Scoria, derived from the main Scoria branch, that includes MDAnalysis support. [file 13321_2017_237_MOESM3_ESM.zip › scoria-1.0.0_mda/docs/docs/html/Information.html]

5. scoria\_mda.Information module — scoria 2.0 documentation


### Navigation

- index
- modules |
- next |
- previous |
- scoria 2.0 documentation »

# 5. scoria\_mda.Information module¶

*class* `scoria_mda.Information.``Information`(*parent\_molecule\_object*)¶
:   A class for storing and accessing information about the elements of a
    scoria\_mda.Molecule object.

    `assign_elements_from_atom_names`(*selection=None*)¶
    :   Determines the elements of all atoms from the atom names. Note that
        this will overwrite any existing element assignments, including those
        explicitly specified in loaded files. Note that this doesn’t populate
        elements\_stripped.

        Wrapper function for `assign_elements_from_atom_names()`

        |  |  |
        | --- | --- |
        | Parameters: | **selection** (*numpy.array*) – An optional numpy.array containing the indices of the atoms to consider when calculating the center of mass. If ommitted, all atoms of the scoria\_mda.Molecule object will be considered. |

    `assign_masses`()¶
    :   Assigns masses to the atoms of the scoria\_mda.Molecule object.

        Wrapper function for `assign_masses()`

        **Note**:
        This will autopopulate the masses according to their element
        identification and takes no input.

    `belongs_to_dna`(*atom\_index*)¶
    :   Checks if the atom is part of DNA.

        Wrapper function for `belongs_to_dna()`

        |  |  |
        | --- | --- |
        | Parameters: | **atom\_index** (*int*) – An int, the index of the atom to consider. |
        | Returns: | A boolean. True if part of dna, False if not. |

    `belongs_to_protein`(*atom\_index*)¶
    :   Checks if the atom is part of a protein. Taken primarily from Amber
        residue names.

        Wrapper function for `belongs_to_protein()`

        |  |  |
        | --- | --- |
        | Parameters: | **atom\_index** (*int*) – An int, the index of the atom to consider. |
        | Returns: | A boolean. True if part of protein, False if not. |

    `belongs_to_rna`(*atom\_index*)¶
    :   Checks if the atom is part of RNA.

        Wrapper function for `belongs_to_rna()`

        |  |  |
        | --- | --- |
        | Parameters: | **atom\_index** (*int*) – An int, the index of the atom to consider. |
        | Returns: | A boolean. True if part of rna, False if not. |

    `define_molecule_chain_residue_spherical_boundaries`()¶
    :   Identifies spheres that bound (encompass) the entire molecule, the
        chains, and the residues. This information is stored in
        scoria\_mda.Molecule.Molecule.hierarchy.

        Requires the `numpy` and `scipy` libraries.

        Wrapper function for
        `define_molecule_chain_residue_spherical_boundaries()`

    `delete_trajectory_frame`(*index*)¶
    :   Removes a given frame from the trajectory.

        Wrapper function for `delete_trajectory_frame()`

        |  |  |
        | --- | --- |
        | Parameters: | **index** (*int*) – Integer of the frame to remove. |

    `get_atom_information`()¶
    :   Retreives the atomic information for the molecule.

        Wrapper function for `get_atom_information()`

        |  |  |
        | --- | --- |
        | Returns: | A masked array containing the atom information. |
        | Return type: | `numpy.ma.MaskedArray` |

        The contents of the array are as follows:

        | member name | dtype | Full Type | Description |
        | --- | --- | --- | --- |
        | record\_name | S6 | six char string | What the atom belongs to |
        | serial | <i8 | 64-bit integer | The index of the atom |
        | name | S5 | five char string | The atom name |
        | resname | S5 | five char string | The residue name |
        | chainid | S1 | one char string | The chain identifier |
        | resseq | <i8 | 64-bit integer | The Residue sequence number |
        | occupancy | <f8 | 64-bit float | Occupancy of atom |
        | tempfactor | <f8 | 64-bit float | Tempature Factor |
        | element | S2 | two char string | The element symbol |
        | charge | S3 | three char string | Charge on the atom |
        | name\_stripped | S5 | five char string | Atom name without space |
        | resname\_stripped | S5 | five char string | Residue name without space |
        | chainid\_stripped | S1 | one char string | Chain identifier without space |
        | element\_stripped | S2 | two char string | Element symbol without space |

        An example for printing the elemental symbols of the first five atoms:

        ```
        >>> atom_info = mol.get_atom_information()
        >>> print atom_info['element_stripped'][0:5]
        ['N' 'C' 'C' 'O' 'C']
        ```

    `get_bonds`()¶
    :   Retreives the bonds beteween atoms as a n x n matrix.

        Wrapper function for `get_bonds()`

        |  |  |
        | --- | --- |
        | Returns: | A binary n x n matrix, where bonds are represented by 1. |
        | Return type: | *numpy.array* |

        An example for finding all atoms bonded with atom 153:

        ```
        >>> bonds = mol.get_bonds()
        >>> for i in range(0,len(bonds)):
        ...     if bonds[153][i] == 1:
        ...             print 153,"-",i
        153 - 152
        153 - 154
        153 - 155
        ```

    `get_bounding_box`(*selection=None*, *padding=0.0*, *frame=None*)¶
    :   Calculates a box that bounds (encompasses) a set of atoms.

        Wrapper function for `get_bounding_box()`

        |  |  |
        | --- | --- |
        | Parameters: | - **selection** (*numpy.array*) – An optional numpy.array containing the indices of   the atoms to consider. If ommitted, all atoms of the   scoria\_mda.Molecule object will be considered. - **padding** (*float*) – An optional float. The bounding box will extend this   many angstroms beyond the atoms being considered. - **frame** (*int*) – An integer indicating at which timestep the center of   mass should be calculated. If ommitted, it defaults to the   first frame of the trajectory. |
        | Returns: | A numpy array representing two 3D points, (min\_x, min\_y, min\_z) and (max\_x, max\_y, max\_z), that bound the molecule. |
        | Return type: | *numpy.array* |

    `get_bounding_sphere`(*selection=None*, *padding=0.0*, *frame=None*)¶
    :   Calculates a sphere that bounds (encompasses) a set of atoms.

        Requires the `numpy` and `scipy` libraries.

        Wrapper function for `get_bounding_sphere()`

        |  |  |
        | --- | --- |
        | Parameters: | - **selection** (*numpy.array*) – An optional numpy.array containing the indices of   the atoms to consider. If ommitted, all atoms of the   scoria\_mda.Molecule object will be considered. - **padding** (*float*) – An optional float. The bounding sphere will extend   this many angstroms beyond the atoms being considered. - **frame** (*int*) – An integer indicating at which timestep the center of   mass should be calculated. If ommitted, it defaults to the   first frame of the trajectory. |
        | Returns: | A tuple containing two elements. The first is a numpy.array representing a 3D point, the (x, y, z) center of the sphere. The second is a float, the radius of the sphere. |
        | Return type: | *tuple* (*numpy.array*, *float*) |

    `get_center_of_mass`(*selection=None*, *frame=None*)¶
    :   Determines the center of mass.

        Wrapper function for `get_center_of_mass()`

        |  |  |
        | --- | --- |
        | Parameters: | - **selection** (*numpy.array*) – The indices of   the atoms to consider when calculating the center of mass.   If ommitted, all atoms of the pymolecule.Molecule object   will be considered. - **frame** (*int*) – The timestep at which the center of mass   should be calculated. If ommitted, it defaults to the first   frame of the trajectory. |
        | Returns: | The x, y, and z coordinates of the center of mass. |
        | Return type: | *numpy.ma* |

        ```
        >>> mol = pymolecule.Molecule()
        >>> mol.load_pdb_into("single_frame.pdb")
        >>> print mol.get_center_of_mass()
        [33.0643089093134 19.135747088722564 16.05629867850796]
        ```

    `get_constants`()¶
    :   Returns a dictionary containing the constants assumed for the molecular model.

        Wrapper function for `get_constants()`

        |  |  |
        | --- | --- |
        | Returns: | The constants assumed by the model. |
        | Return type: | *dict* |

        | Dictionary Keys | Value Type | Contains |
        | --- | --- | --- |
        | mass\_dict | dict{str:float} | The mass of elements |
        | rna\_residues | list(str) | RNA residue names |
        | f8\_fields | list(str) | Atom Information floats |
        | vdw\_dict | dict{str:float} | Van der Waals force of elements |
        | i8\_fields | list(str) | Atom Information integers |
        | protein\_residues | list(str) | Protein residue names |
        | bond\_length\_dict | dict{str:float} | Element-pair bond length |
        | element\_names\_with\_two\_letters | list(str) | Element symbols with 2 letters |
        | max\_number\_of\_bonds\_permitted | dict{str:int} | Max bonds per element |
        | dna\_residues | list(str) | DNA reside names |

    `get_coordinates`(*frame=None*)¶
    :   Returns the set of coordinates from the specified frame.

        Wrapper function for `get_coordinates()`

        |  |  |
        | --- | --- |
        | Parameters: | **frame** (*int*) – The timestep from which the coordinates shoule be returned. If ommitted, it defaults to the first frame of the trajectory. |
        | Returns: | The set of coordinates from the specified frame. ``` [[x1, y1, z1], ... [xn, yn, zn]] ``` |
        | Return type: | *numpy.array* |

        ```
        >>> print mol.get_coordinates()
        [[ -30.85199928  -81.45800018  365.05499268]
         [ -31.99500084  -80.69300079  365.66900635]
         [ -32.0530014   -81.13200378  367.18200684]
         ..., 
         [ -27.54199982  -96.25099945  402.83700562]
         [ -23.54199982  -94.7539978   400.41900635]
         [ -22.86100006  -93.72499847  400.55300903]]
         
        >>> print mol.get_coordinates(2)
        [[ -28.88899994  -80.45700073  365.51699829]
         [ -30.20000076  -79.73699951  365.99700928]
         [ -30.90699959  -80.5510025   367.13000488]
         ..., 
         [ -26.0189991   -97.28099823  403.52600098]
         [ -23.2140007   -94.73999786  400.94699097]
         [ -22.52899933  -93.73300171  400.81399536]]
        ```

    `get_coordinates_undo_point`()¶
    :   NEEDS CLARIFICATION.
        Retreives a previously save set of coordinates to revert to.

        Wrapper function for `get_coordinates_undo_point()`

        |  |  |
        | --- | --- |
        | Returns: | A set of coordinates from which to return to. |
        | Return type: | *numpy.array* or *None* |

    `get_default_trajectory_frame`()¶
    :   Retreives the default trajectory frame index.

        |  |  |
        | --- | --- |
        | Returns: | An *int* representing the index of the default trajectory frame. |

    `get_filename`()¶
    :   Returns the filename that the molecule was originally loaded from.

        Wrapper function for `get_filename()`

        |  |  |
        | --- | --- |
        | Returns: | The name of the file. |
        | Return type: | `str` |

        ```
        >>> mol = pymolecule.Molecule()
        >>> mol.load_pdb_into("single_frame.pdb")
        >>> print mol.get_filename()
        single_frame.pdb
        ```

    `get_geometric_center`(*selection=None*, *frame=None*)¶
    :   Determines the geometric center of the molecule.

        Wrapper function for `get_geometric_center()`

        |  |  |
        | --- | --- |
        | Parameters: | - **selection** (*numpy.array*) – The indices of   the atoms to consider when calculating the geometric.   If ommitted, all atoms of the pymolecule.Molecule object   will be considered. - **frame** (*int*) – The timestep at which the geometric center   should be calculated. If ommitted, it defaults to the first   frame of the trajectory. |
        | Returns: | The x, y, and z coordinates of the geometric center. |
        | Return type: | *numpy.array* |

        ```
        >>> mol = pymolecule.Molecule()
        >>> mol.load_pdb_into("single_frame.pdb")
        >>> print mol.get_geometric_center()
        [ 33.09860848  19.1221197   16.0426808 ]
        ```

    `get_hierarchy`()¶
    :   NEEDS CLARIFICATION.

        Wrapper function for `get_hierarchy()`

        |  |  |
        | --- | --- |
        | Returns: | A dictionary? |
        | Return type: | *dict* |

    `get_remarks`()¶
    :   Returns the remarks from the file the molecule was loaded from.

        Wrapper function for `get_remarks()`

        |  |  |
        | --- | --- |
        | Returns: | The remarks from the file an a list of strings. |
        | Return type: | *list* |

        ```
        >>> mol = pymolecule.Molecule()
        >>> mol.load_pdb_into("single_frame.pdb")
        >>> print mol.get_remarks()
        [' This is a remark.']
        ```

    `get_total_mass`(*selection=None*)¶
    :   Returns the total mass of all atoms within the molecule, or of a given
        selection.

        Wrapper function for `get_total_mass()`

        |  |  |
        | --- | --- |
        | Parameters: | **selection** (*numpy.array*) – The indices of the atoms to consider when calculating the geometric. If ommitted, all atoms of the pymolecule.Molecule object will be considered. |
        | Returns: | The total mass of the atom or selection |
        | Return type: | *float* |

        ```
        >>> print mol.get_total_mass()
        5289.1729999999998
        ```

    `get_total_number_of_atoms`(*selection=None*, *frame=None*)¶
    :   Counts the number of atoms.

        Wrapper function for
        `get_total_number_of_atoms()`

        |  |  |
        | --- | --- |
        | Parameters: | - **selection** (*numpy.array*) – An optional numpy.array containing the indices of   the atoms to count. If ommitted, all atoms of the   pymolecule.Molecule object will be considered. - **frame** (*int*) – An integer indicating at which timestep the center of   mass should be calculated. If ommitted, it defaults to the   first frame of the trajectory. |
        | Returns: | The total number of atoms. |
        | Return type: | *int* |

    `get_total_number_of_heavy_atoms`(*selection=None*)¶
    :   Counts the number of heavy atoms (i.e., atoms that are not
        hydrogens).

        Wrapper function for
        `get_total_number_of_heavy_atoms()`

        |  |  |
        | --- | --- |
        | Parameters: | **selection** (*numpy.array*) – An optional numpy.array containing the indices of the atoms to count. If ommitted, all atoms of the pymolecule.Molecule object will be considered. |
        | Returns: | The total number of heavy (non-hydrogen) atoms. |
        | Return type: | *int* |

    `get_trajectory`()¶
    :   Returns the trajectory for the molecule.

        Wrapper function for `get_trajectory()`

        |  |  |
        | --- | --- |
        | Returns: | The set of all coordinates. ``` [[[x11, y11, z11], ... [x1n, y1n, z1n]],  ...,  [[xm1, ym1, zm1], ... [xmn, ymn, zmn]]] ``` |
        | Return type: | *numpy.array* |

        ```
        >>> for coord in mol.get_trajectory():
        >>>     print coord
        >>>     print
        [[ -30.85199928  -81.45800018  365.05499268]
         [ -31.99500084  -80.69300079  365.66900635]
         [ -32.0530014   -81.13200378  367.18200684]
         ..., 
         [ -27.54199982  -96.25099945  402.83700562]
         [ -23.54199982  -94.7539978   400.41900635]
         [ -22.86100006  -93.72499847  400.55300903]]

        [[ -30.6779995   -81.32499695  365.73199463]
         [ -31.88100052  -80.38600159  366.0289917 ]
         [ -32.40399933  -80.62799835  367.45700073]
         ..., 
         [ -27.44400024  -96.71099854  402.64700317]
         [ -23.79199982  -94.58899689  400.63598633]
         [ -23.10700035  -93.56300354  400.79598999]]
         <more>
        ```

    `get_trajectory_frame_count`()¶
    :   Returns the number of frames in \_\_trajectory.

        Wrapper function for `get_trajectory_frame_count()`

        |  |  |
        | --- | --- |
        | Returns: | The number of frames in the trajectory. |
        | Return type: | *int* |

    `insert_trajectory_frame`(*index*, *coordinates*)¶
    :   Inserts a new coordinate frame at the end of the trajectory.

        Wrapper function for `insert_trajectory_frame()`

        |  |  |
        | --- | --- |
        | Parameters: | - **coordinates** (*numpy.array*) – A single frame of coordinates to append. - **index** (*int*) – The location where the frame should be added. |

    `resseq_reindex`()¶
    :   Reindexes the resseq field of the atoms in the molecule, starting
        with 1.

        Wrapper function for `resseq_reindex()`

    `serial_reindex`()¶
    :   Reindexes the serial field of the atoms in the molecule, starting
        with 1.

        Wrapper function for `serial_reindex()`

    `set_atom_information`(*atom\_information*)¶
    :   Sets the \_\_atom\_information variable. See
        `get_atom_information()` for
        information on the numpy.array structure.

        Wrapper function for `set_atom_information()`

        |  |  |
        | --- | --- |
        | Parameters: | **atom\_information** (*numpy.array*) – An array containing details on the constituent atoms. |

    `set_bonds`(*bonds*)¶
    :   Sets the \_\_bonds variable. See
        `get_bonds()` for additional
        information.

        Wrapper function for `set_bonds()`

        |  |  |
        | --- | --- |
        | Parameters: | **bonds** (*numpy.array*) – A binary n x n matrix containing bonding information. |

    `set_coordinates`(*coordinates*, *frame=None*)¶
    :   Sets a specified frame of the \_\_trajectory variable.

        Wrapper function for `set_coordinates()`

        |  |  |
        | --- | --- |
        | Parameters: | - **coordinates** (*numpy.array*) – An array of atomic coordinates. - **frame** (*int*) – An integer represeting the frame of the trajectory to be modified |

    `set_coordinates_undo_point`(*coordinates\_undo\_point*)¶
    :   Sets the \_\_coordinates\_undo\_point variable.

        Wrapper function for `set_coordinates_undo_point()`

        |  |  |
        | --- | --- |
        | Parameters: | **coordinates\_undo\_point** (*numpy.array*) – A coordinate set to revert to after modification. |

    `set_default_trajectory_frame`(*frame*)¶
    :   Se’s the default trajectory frame index for various calculations.

        |  |  |
        | --- | --- |
        | Parameters: | **frame** (*int*) – The default frame for coordinate selection. |

    `set_filename`(*filename*)¶
    :   Sets the \_\_filename variable. Note: this does not reload or modify the
        molecule in anyway.

        Wrapper function for `set_filename()`

        |  |  |
        | --- | --- |
        | Parameters: | **filename** (*str*) – String representation of the filename. |

    `set_hierarchy`(*hierarchy*)¶
    :   Sets the \_\_hierarchy variable.
        DEPRECIATED?

        Wrapper function for `set_hierarchy()`

    `set_remarks`(*remarks*)¶
    :   Sets the \_\_remarks variable.

        Wrapper function for `set_remarks()`

        |  |  |
        | --- | --- |
        | Parameters: | **remarks** (*list(str)*) – List containing remarks. |

    `set_trajectory`(*trajectory*)¶
    :   Sets the \_\_trajectory variable.

        Wrapper function for `set_trajectory()`

        |  |  |
        | --- | --- |
        | Parameters: | **trajectory** (*numpy.array*) – An array of atomic coordinates. |

#### Previous topic

4. pymolecule.Geometry module

#### Next topic

6. pymolecule.Manipulation module

### This Page

- Show Source

### Quick search

### Navigation

- index
- modules |
- next |
- previous |
- PyMolecule 2.0 documentation »

© Copyright 2016, Jacob Durrant.
Created using Sphinx 1.4.6.
