# Supplementary material for: Scoria: a Python module for manipulating 3D molecular data
Source: J Cheminform. 2017 Sep 18;9:52. doi: 10.1186/s13321-017-0237-8 (PMC5603467; doi:10.1186/s13321-017-0237-8)
Supplement: Supplementary file 3 — Additional file 3. An archived version of Scoria, derived from the main Scoria branch, that includes MDAnalysis support. [file 13321_2017_237_MOESM3_ESM.zip › scoria-1.0.0_mda/docs/docs/html/Manipulation.html]

6. scoria\_mda.Manipulation module — scoria 2.0 documentation


### Navigation

- index
- modules |
- next |
- previous |
- scoria 2.0 documentation »

# 6. scoria\_mda.Manipulation module¶

*class* `scoria_mda.Manipulation.``Manipulation`(*parent\_molecule\_object*)¶
:   A class for translating and rotating the atomic coordinates of a
    scoria\_mda.Molecule object

    `coordinate_undo`()¶
    :   Resets the coordinates of all atoms to those saved using the
        set\_coordinate\_undo\_point function.

    `rotate_molecule_around_a_line_between_atoms`(*line\_point1\_index*, *line\_point2\_index*, *rotate*)¶
    :   Rotate the molecular model about a line segment. The end points of
        the line segment are atoms of specified indices.

        Wrapper function for
        `rotate_molecule_around_a_line_between_atoms()`

        |  |  |
        | --- | --- |
        | Parameters: | - **line\_point1\_index** (*int*) – An int, the index of the first atom at one   end of the line segment. - **line\_point2\_index** (*int*) – An int, the index of the second atom at   the other end of the line segment. - **rotate** (*float*) – A float, the angle of rotation, in radians. |

    `rotate_molecule_around_a_line_between_points`(*line\_point1*, *line\_point2*, *rotate*)¶
    :   Rotate the molecular model about a line segment. The end points of
        the line segment are explicitly specified coordinates.

        Wrapper function for
        `rotate_molecule_around_a_line_between_points()`

        |  |  |
        | --- | --- |
        | Parameters: | - **line\_point1** (*numpy.array*) – A numpy.array (x, y, z) corresponding to one end   of the line segment. - **line\_point2** (*numpy.array*) – A numpy.array (x, y, z) corresponding to the   other end of the line segment. - **rotate** (*float*) – A float, the angle of rotation, in radians. |

    `rotate_molecule_around_pivot_atom`(*pivot\_index*, *thetax*, *thetay*, *thetaz*)¶
    :   Rotate the molecular model around a specified atom.

        Requires the `numpy` library.

        Wrapper function for `rotate_molecule_around_pivot_atom()`

        |  |  |
        | --- | --- |
        | Parameters: | - **pivot\_index** (*int*) – An int, the index of the atom about which the   molecular model will be rotated. - **thetax** (*float*) – A float, the angle to rotate relative to the x axis,   in radians. - **thetay** (*float*) – A float, the angle to rotate relative to the y axis,   in radians. - **thetaz** (*float*) – A float, the angle to rotate relative to the z axis,   in radians. |

    `rotate_molecule_around_pivot_point`(*pivot*, *thetax*, *thetay*, *thetaz*)¶
    :   Rotate the molecular model around a specified atom.

        Requires the `numpy` library.

        Wrapper function for `rotate_molecule_around_pivot_point()`

        |  |  |
        | --- | --- |
        | Parameters: | - **pivot** (*numpy.array*) – A numpy.array, the (x, y, z) coordinate about which   the molecular model will be rotated. - **thetax** (*float*) – A float, the angle to rotate relative to the x axis,   in radians. - **thetay** (*float*) – A float, the angle to rotate relative to the y axis,   in radians. - **thetaz** (*float*) – A float, the angle to rotate relative to the z axis,   in radians. |

    `set_atom_location`(*atom\_index*, *new\_location*)¶
    :   Translates the entire molecular model (without rotating) so that the
        atom with the specified index is located at the specified coordinate.

        Wrapper function for `set_atom_location()`

        |  |  |
        | --- | --- |
        | Parameters: | - **atom\_index** (*int*) – An int, the index of the target atom. - **new\_location** (*numpy.array*) – A numpy.array specifying the new (x, y, z)   coordinate of the specified atom. |
        | Returns: | A numpy.array specifying the (delta\_x, delta\_y, delta\_z) vector by which the pmolecule.Molecule was translated. |

    `set_coordinate_undo_point`()¶
    :   Sets (“saves”) the undo point of the atom coordinates. Any
        subsequent manipulations of atomic coordinates can be “undone” by
        reseting to this configuration via the coordinate\_undo function.

    `translate_molecule`(*delta*)¶
    :   Translate all the atoms of the molecular model by a specified
        vector.

        Wrapper function for `translate_molecule()`

        |  |  |
        | --- | --- |
        | Parameters: | **delta** (*numpy.array*) – A numpy.array (delta\_x, delta\_y, delta\_z) specifying the amount to move each atom along the x, y, and z coordinates. |

#### Previous topic

5. scoria\_mda.Information module

#### Next topic

7. scoria\_mda.OtherMolecules module

### This Page

- Show Source

### Quick search

### Navigation

- index
- modules |
- next |
- previous |
- scoria 2.0 documentation »

© Copyright 2016, Jacob Durrant.
Created using Sphinx 1.4.6.
