# Supplementary material for: Scoria: a Python module for manipulating 3D molecular data
Source: J Cheminform. 2017 Sep 18;9:52. doi: 10.1186/s13321-017-0237-8 (PMC5603467; doi:10.1186/s13321-017-0237-8)
Supplement: Supplementary file 3 — Additional file 3. An archived version of Scoria, derived from the main Scoria branch, that includes MDAnalysis support. [file 13321_2017_237_MOESM3_ESM.zip › scoria-1.0.0_mda/docs/docs/html/py-modindex.html]

Python Module Index — scoria 2.0 documentation


### Navigation

- index
- modules |
- scoria 2.0 documentation »

# Python Module Index

**p**

|  |  |  |
| --- | --- | --- |
|  |  |  |
|  | **p** |  |
|  | `scoria` |  |
|  | `scoria_mda.AtomsAndBonds` |  |
|  | `scoria_mda.FileIO` |  |
|  | `scoria_mda.Geometry` |  |
|  | `scoria_mda.Information` |  |
|  | `scoria_mda.Manipulation` |  |
|  | `scoria_mda.Molecule` |  |
|  | `scoria_mda.OtherMolecules` |  |
|  | `scoria_mda.Quaternion` |  |
|  | `scoria_mda.Selections` |  |

### Quick search

### Navigation

- index
- modules |
- scoria 2.0 documentation »

© Copyright 2016, Jacob Durrant.
Created using Sphinx 1.4.6.
