# Supplementary material for: Scoria: a Python module for manipulating 3D molecular data
Source: J Cheminform. 2017 Sep 18;9:52. doi: 10.1186/s13321-017-0237-8 (PMC5603467; doi:10.1186/s13321-017-0237-8)
Supplement: Supplementary file 3 — Additional file 3. An archived version of Scoria, derived from the main Scoria branch, that includes MDAnalysis support. [file 13321_2017_237_MOESM3_ESM.zip › scoria-1.0.0_mda/docs/docs/html/pymolecule.dumbpy.html]

scoria\_mda.dumbpy package — scoria 2.0 documentation


### Navigation

- index
- modules |
- scoria 2.0 documentation »

# scoria\_mda.dumbpy package¶

## Submodules¶

## scoria\_mda.dumbpy.Array module¶

Note that this code is NOT a replacement for numpy. It imitates numpy just
well enough to run some of the scoria functions that couldn’t run
otherwise. Installing numpy/scipy is strongly recommended.

*class* `scoria_mda.dumbpy.Array.``Array1D`(*lst*, *dtype=''*)¶
:   Bases: `scoria_mda.dumbpy.Array.ArrayParent`

    A 1D Array.

    `astype`(*dtype*)¶
    :   Casts this array as a given type.

        Args:
        :   dtype – The type to cast.

        Returns:
        :   Self, in case chaining is required.

    `set_shape`()¶
    :   Sets the shape of this array.

    `type` *= '1D'*¶

*class* `scoria_mda.dumbpy.Array.``Array2D`(*lst*, *dtype='float'*)¶
:   Bases: `scoria_mda.dumbpy.Array.ArrayParent`

    A 2D Array.

    `T`¶
    :   Returns the transpose of this array.

        Returns:
        :   An Array2D, the transpose.

    `set_shape`()¶
    :   Sets the shape of this array.

    `type` *= '2D'*¶

*class* `scoria_mda.dumbpy.Array.``ArrayParent`¶
:   The parent of all Array classes.

    `copy`()¶

    `lst` *= []*¶

    `shape` *= ()*¶

    `type` *= ''*¶

*class* `scoria_mda.dumbpy.Array.``RecArray`(*dict*, *dtypes=None*)¶
:   A record array.

    `astype`(*dtype*)¶
    :   Cast this array as a certain type.

        Returns:
        :   This array, in case chaining is required.

    `clone`()¶
    :   Makes a clone of this array.

        Returns:
        :   A RecArray, the clone.

    `copy`()¶
    :   Makes a copy of this array.

        Returns:
        :   A RecArray, the copy.

    `dict` *= {}*¶

    `dtype` *= <scoria\_mda.dumbpy.DType.dtype instance>*¶

    `ndim` *= 1*¶

    `type` *= 'Rec'*¶

`scoria_mda.dumbpy.Array.``array`(*lst*, *dtype=''*)¶
:   Determines whether or not a 1D or 2D array should be used.

    Args:
    :   lst – A list to convert to an array.

    Returns:
    :   An Array2D or Array1D object, as required.

## scoria\_mda.dumbpy.DType module¶

*class* `scoria_mda.dumbpy.DType.``dtype`(*descr*)¶
:   A class that stores variable-type information.

    `clone`()¶
    :   Make a clone of this dtype.

        Returns:
        :   A DType, the clone.

    *static* `convert`(*tp*, *val*)¶
    :   Convert a variable given a variable type.

        Args:
        :   tp – The variable type.
            val – The variable to convert.

        Returns:
        :   The converted variable.

    `descr` *= []*¶

    `names`¶
    :   Get the names of the variable types.

        Returns:
        :   A list of strings, the variable types.

    `names_ordered` *= []*¶

## scoria\_mda.dumbpy.Support module¶

`scoria_mda.dumbpy.Support.``to_list`(*arr*)¶
:   Convert an array to a list.

    Args:
    :   arr – A 1D or 2D array.

    Returns:
    :   The list.

`scoria_mda.dumbpy.Support.``var_type`(*var*)¶
:   A helper function to identify a variable’s type.

    Args:
    :   var – The variable.

    Returns:
    :   A string, the variable type.

## scoria\_mda.dumbpy.Utils module¶

`scoria_mda.dumbpy.Utils.``all_same_num`(*dims*, *num*, *dtype='float'*)¶
:   Make a square array filled with the same values.

    Args:
    :   dims – An int, the dimension of the square array.
        num – The number to fill.
        dtype – A string, the variable type.

    Returns:
    :   An array.

`pymolecule.dumbpy.Utils.``append`(*arr1*, *to\_append*)¶
:   Add a value to the end of an array.

    Args:
    :   arr – The source array.
        to\_append – The value to append.

    Returns:
    :   An array, with the value appended.

`pymolecule.dumbpy.Utils.``append_fields`(*arr*, *field\_name*, *data*)¶
:   Append columns to a rec array.

    Args:
    :   arr – The rec array.
        field\_name – A string, the new field name.
        data – The data to add under that field name.

    Returns:
    :   A rec array.

`pymolecule.dumbpy.Utils.``arange`(*start*, *stop*, *step*, *dtype='f8'*)¶
:   An array with values that begin at start and end at stop, spaced step
    apart.

    > Args:
    > :   start – The starting value.
    >     stop – The stopping value.
    >     step – The distance between values.
    >     dtype – The variable type (string).
    >
    > Returns:
    > :   An array, with the specified equidistant values.

`pymolecule.dumbpy.Utils.``defchararray_add`(*arr*, *addit*)¶
:   Adds a string to each element in a strig array.

    Args:
    :   arr – The string array.
        addit – The string to add.

    Returns:
    :   A string array.

`pymolecule.dumbpy.Utils.``defchararray_lstrip`(*arr, chars=[' ', '\t']*)¶
:   Strips left spaces from the strings in a string array.

    Args:
    :   arr – The string array.
        chars – A list of the characters to consider white space.

    Returns:
    :   A string array.

`pymolecule.dumbpy.Utils.``defchararray_rjust`(*arr*, *width*)¶
:   Right justifies the strings in a string array.

    Args:
    :   arr – The string array.
        width – The width of the new elements.

    Returns:
    :   A string array.

`pymolecule.dumbpy.Utils.``defchararray_split`(*arr*, *num=-1*)¶
:   Splits the strings in a string array.

    Args:
    :   arr – The string array.
        num – The number of splits to make. Defaults to -1 (all).

    Returns:
    :   An array of string arrays.

`pymolecule.dumbpy.Utils.``defchararray_strip`(*arr*)¶
:   Strips spaces from the strings in a string array.

    Args:
    :   arr – The string array.

    Returns:
    :   A string array.

`pymolecule.dumbpy.Utils.``defchararray_upper`(*arr*)¶
:   Make the strings in a string array uppercase.

    Args:
    :   arr – The string array.

    Returns:
    :   A string array, all upper case.

`pymolecule.dumbpy.Utils.``delete`(*arr*, *indx\_to\_delete*)¶
:   Delete the values in an array.

    Args:
    :   arr – The source array.
        axis – The indecies to delete.

    Returns:
    :   The new array, with elements removed.

`pymolecule.dumbpy.Utils.``empty`(*dims*, *dtype='float'*)¶
:   Make a square array filled with zeros.

    Args:
    :   dims – An int, the dimension of the square array.
        dtype – A string, the variable type.

    Returns:
    :   An array.

`pymolecule.dumbpy.Utils.``extrema`(*func*, *arr*, *axis=0*)¶
:   Calculates the extrema (max or min) of an array.

    Args:
    :   func – The function to apply, max or min.
        arr – The array.
        axis – The axis (0 by default).

    Returns:
    :   The extrema value across the axis.

`pymolecule.dumbpy.Utils.``fabs`(*num*)¶
:   The aboslute value function.

    Args:
    :   num – A number.

    Returns:
    :   fabs(num)

`pymolecule.dumbpy.Utils.``genfromtxt`(*fname*, *dtype=''*, *names=[]*, *delimiter=[]*)¶
:   Generates an array from a text file.

    Args:
    :   fname – A string, the file name.
        dtype – The variable types.
        names – A list of string, the names of the variables.
        delimiter – A list of numbers, the legths of each field.

    Returns:
    :   A RecArray object.

`pymolecule.dumbpy.Utils.``get_col`(*lst*, *num*)¶
:   Return the column of a 2D array.

    Args:
    :   lst – The array.
        num – The column index.

    Returns:
    :   The specified column.

`pymolecule.dumbpy.Utils.``identity`(*dimen*)¶
:   A 2D identity array.

    Args:
    :   dimen – The dimension of the square array.

    Returns:
    :   An identity array.

`pymolecule.dumbpy.Utils.``insert`(*arr*, *indx*, *val*)¶
:   Insert a value into an array.

    Args:
    :   arr – The source array.
        indx – The index at which to insert the value.
        val – The value to insert.

    Returns:
    :   An array, with the value inserted.

`pymolecule.dumbpy.Utils.``logical_and`(*arr1*, *arr2*)¶
:   Applies the logical and element wise.

    Args:
    :   arr1 – The first array.
        arr2 – The second array.

    Returns:
    :   An boolean array.

`pymolecule.dumbpy.Utils.``logical_not`(*arr*)¶
:   Applies the logical not element wise.

    Args:
    :   arr – The first array.

    Returns:
    :   An boolean array.

`pymolecule.dumbpy.Utils.``logical_or`(*arr1*, *arr2*)¶
:   Applies the logical or element wise.

    Args:
    :   arr1 – The first array.
        arr2 – The second array.

    Returns:
    :   An boolean array.

`pymolecule.dumbpy.Utils.``mean`(*arr*, *axis=0*)¶
:   Calculates the mean of an array along an axis.

    Args:
    :   arr – The source array.
        axis – The axis. Defaults to 0.

    Returns:
    :   The mean along the axis.

`pymolecule.dumbpy.Utils.``nonzero`(*arr*)¶
:   Identifies which entries in an array are not zero.

    Args:
    :   arr – The array.

    Returns:
    :   A list of lists (the indecies along each dimension).

`pymolecule.dumbpy.Utils.``norm`(*arr*)¶
:   The length of a 1D vector.

    Args:
    :   arr – The 1D vector.

    Returns:
    :   A number, the vector’s length.

`pymolecule.dumbpy.Utils.``ones`(*dims*, *dtype='float'*)¶
:   Make a square array filled with ones.

    Args:
    :   dims – An int, the dimension of the square array.
        dtype – A string, the variable type.

    Returns:
    :   An array.

`pymolecule.dumbpy.Utils.``power`(*num*, *p*)¶
:   Calculate the power of a number.

    Args:
    :   num – The number.
        p – The exponent.

    Returns:
    :   The number num ^ p.

`pymolecule.dumbpy.Utils.``setdiff1d`(*arr1*, *arr2*)¶
:   Get the difference between two arrays.

    Args:
    :   arr1 – The first array.
        arr2 – The second array.

    Returns:
    :   The difference between the arrays.

`pymolecule.dumbpy.Utils.``sqrt`(*num*)¶
:   The square root function.

    Args:
    :   num – A number.

    Returns:
    :   sqrt(num)

`pymolecule.dumbpy.Utils.``stack_arrays`(*arr\_list*, *usemask=False*)¶
:   Like vstack, but for rec arrays.

    Args:
    :   arr\_list – A list of rec arrays.
        usermask – Not sure. Always false, not used.

    Returns:
    :   A rec array.

`pymolecule.dumbpy.Utils.``sum`(*arr*, *axis=0*)¶
:   Sum the values of an array along an axis.

    Args:
    :   arr – The source array.
        axis – The axis. Defaults to 0.

    Returns:
    :   The sum along the axis.

`pymolecule.dumbpy.Utils.``unique`(*arr*)¶
:   Return the unique values in an array.

    Args:
    :   arr – The source array.

    Returns:
    :   An array, with unique values.

`pymolecule.dumbpy.Utils.``vstack`(*arrays*)¶
:   Stacks arrays.

    Args:
    :   arrays – A list of arrays.

    Returns:
    :   A string array.

`pymolecule.dumbpy.Utils.``zeros`(*dims*, *dtype='float'*)¶
:   Make a square array filled with zeros.

    Args:
    :   dims – An int, the dimension of the square array.
        dtype – A string, the variable type.

    Returns:
    :   An array.

## Module contents¶

Loads external modules (numpy, scipy) if available. Otherwise, uses cheap
imitations.

`pymolecule.dumbpy.``class_dependency`(*action*, *dependency*, *error\_flag=True*)¶
:   Determines whether or not a given dependency is available.

    Args:
    :   action – A string describing the action you’d like to
        :   perform.

        dependency – A string, the dependency required for that action.

    Returns:
    :   A boolean, true if the dependency is available. Prints a message
        :   otherwise.

`pymolecule.dumbpy.``get_col`(*arr*, *num*)¶

### Table Of Contents

- pymolecule.dumbpy package
  - Submodules
  - pymolecule.dumbpy.Array module
  - pymolecule.dumbpy.DType module
  - pymolecule.dumbpy.Support module
  - pymolecule.dumbpy.Utils module
  - Module contents

### This Page

- Show Source

### Quick search

### Navigation

- index
- modules |
- PyMolecule 2.0 documentation »

© Copyright 2016, Jacob Durrant.
Created using Sphinx 1.4.6.
